# Decline in infection-related morbidities following drug-mediated reductions in the intensity of *Schistosoma* infection: A systematic review and meta-analysis

**DOI:** 10.1371/journal.pntd.0005372

**Published:** 2017-02-17

**Authors:** Gisele Andrade, David J. Bertsch, Andrea Gazzinelli, Charles H. King

**Affiliations:** 1 Escola de Enfermagem, Universidade Federal de Minas Gerais, Belo Horizonte, MG, Brazil; 2 Center for Global Health and Diseases, Case Western Reserve University, Cleveland, Ohio, United States of America; 3 Department of Biology, Case Western Reserve University, Cleveland, Ohio, United States of America; 4 Instituto Nacional de Ciência e Tecnologia em Doenças Tropicais (INCT-DT), Belo Horizonte, MG, Brazil; 5 Schistosomiasis Consortium for Operational Research and Evaluation, University of Georgia, Athens, Georgia, United States of America; University of California Berkeley, UNITED STATES

## Abstract

**Background:**

Since 1984, WHO has endorsed drug treatment to reduce *Schistosoma* infection and its consequent morbidity. Cross-sectional studies suggest pre-treatment correlation between infection intensity and risk for *Schistosoma*-related pathology. However, evidence also suggests that post-treatment reduction in intensity may not reverse morbidity because some morbidities occur at all levels of infection, and some reflect permanent tissue damage. The aim of this project was to systematically review evidence on drug-based control of schistosomiasis and to develop a quantitative estimate of the impact of post-treatment reductions in infection intensity on prevalence of infection-associated morbidity.

**Methodology/Principal findings:**

This review was registered at inception with PROSPERO (CRD42015026080). Studies that evaluated morbidity before and after treatment were identified by online searches and searches of private archives. Post-treatment odds ratios or standardized mean differences were calculated for each outcome, and these were correlated to treatment-related egg count reduction ratios (ERRs) by meta-regression. A greater ERR correlated with greater reduction in odds of most morbidities. Random effects meta-analysis was used to derive summary estimates: after treatment of *S*. *mansoni* and *S*. *japonicum*, left-sided hepatomegaly was reduced by 54%, right-sided hepatomegaly by 47%, splenomegaly by 37%, periportal fibrosis by 52%, diarrhea by 53%, and blood in stools by 75%. For *S*. *haematobium*, hematuria was reduced by 92%, proteinuria by 90%, bladder lesions by 86%, and upper urinary tract lesions by 72%. There were no consistent changes in portal dilation or hemoglobin levels. In sub-group analysis, age, infection status, region, parasite species, and interval to follow-up were associated with meaningful differences in outcome.

**Conclusion/Significance:**

While there are challenges to implementing therapy for schistosomiasis, and praziquantel therapy is not fully curative, reductions in egg output are significantly correlated with decreased morbidity and can be used to project diminution in disease burden when contemplating more aggressive strategies to minimize infection intensity.

## Author summary

Schistosomiasis is the disease caused by infection with *Schistosoma* parasitic flukes. Depending on the infecting species, chronic *Schistosoma* infection can cause a variety of pathologies including liver and spleen enlargement, fibrosis and hypertension of the portal vein of the liver, or bladder ulceration and deformities and kidney blockage. Infection can also cause anemia, diarrhea, abdominal pain, and decreased physical fitness. In our study, we quantified the reductions in prevalence of infection-related morbidities among populations with *Schistosoma* infection, as achieved by giving one or more drug treatments. We systematically reviewed 71 available reports of *Schistosoma*-related morbidity reduction and determined, based on a meta-analysis of the primary data, that the odds of persisting morbidity progressively decrease when greater post-treatment reductions in parasite burden are achieved, as reflected by reduced egg counts in standard diagnostic testing. This suggests that repeated or more effective anti-parasite drug treatment will be a valuable tool for greater reduction of *Schistosoma*-related patient morbidities in affected areas.

## Introduction

Schistosomiasis, caused by *Schistosoma* spp. blood flukes, is one of the most prevalent parasitic diseases in the world, with more than 240 million people infected and 800 million at risk of infection [1]. Chronic schistosomiasis is the form of infection that is predominant in endemic areas, which bear the greatest disease impact from long-lived *Schistosoma* infections [2]. Because of pathology caused by parasite eggs deposited into human tissues, schistosomiasis turns into a multi-year inflammatory disease of the intestine, liver, urinary tract, and other critical organs. Adult schistosome worms colonize the human body for years, excreting eggs every day. These eggs provoke granulomatous inflammation in order to achieve translocation from the venous circulation to either the bowel or bladder lumena. If eggs do not succeed in leaving the body in excreta, they remain trapped in nearby tissues, causing persistent chronic inflammation and scarring [3, 4].

For many years, clinical studies of the morbidity related to schistosomiasis have mainly focused on specific forms of advanced organ pathology and focal clinical signs. These include hepatosplenomegaly, periportal fibrosis, portal hypertension, bladder deformity, hydronephrosis, hematuria, abdominal pain and related organ scarring [5–7]. More recent research has also put emphasis on systemic morbidities associated with *Schistosoma* infection such as anemia, growth stunting, impaired cognition, undernutrition, diarrhea, and decreased physical fitness; however, this additional burden of schistosomiasis was not well studied in many older works, and until the 1990s, improvement in these outcomes was not generally appreciated as a potential benefit of morbidity control [8].

Schistosomiasis control is a constant challenge for endemic regions and their public health services, mainly due to difficulties in preventing early infection and frequent reinfection. Several strategies, such as environmental control of the intermediary host, provision of safe water, and medical treatment have been used, singly and in combination [9]. However, since the 1980s, especially with the advent of praziquantel, drug-based control of morbidity related to infection has been the primary WHO strategy for schistosomiasis control, with treatment given mainly through community- and school-based mass treatment [10]. The usual parameters employed to assess the effectiveness of treatment have been its effects on the intensity and prevalence of infection. Although there is an association between intensity of infection and the presence and severity of morbidity [11–14], the correlation is imperfect, and monitoring infection intensity may provide only an indirect means to gauge morbidity risk. Individuals with low intensity infections can express all forms of the disease, and thus we must consider that the morbidity caused by *Schistosoma* infection can also be triggered by just the presence of infection [8, 14–18].

In recent years, millions of people have been treated in different contexts and, in general, prevalence of morbidity has been reduced after treatment [7, 19–22]. Nevertheless, studies of morbidity reduction related to drug treatment have had some conflicting results [23–26], which may be a reflection of differences in follow-up after treatment, methods used to measure morbidities, the *Schistosoma* species, the presence of co-infections (especially malaria), the type of population and the region, the initial prevalence of infection, the incidence of reinfection, and other factors [7, 27]. Despite the potential benefits of treatment, many affected persons have not yet been reached by treatment programs [28].

Given this context, and that one of the main objectives of schistosomiasis control programs has been to achieve reductions in morbidity associated with *Schistosoma* infection [29], there is a need to accurately quantify the reduction of morbidity levels as a result of chemotherapy intervention, so that the specific benefits of more intensive interventions can be identified. To do this, we developed a meta-analysis to evaluate the impact of drug treatment and the reduction of infection intensity on levels of morbidity associated with schistosomiasis. In specific, because a quantitative link can be used in cost-effectiveness analysis comparing different treatments strategies, we aimed to determine the numerical relationship between egg reduction rates (ERR, observed in post-treatment diagnostic testing [30]) and the reduced risk of morbidity after treatment.

## Methods

### Ethics statement

The data used in this project were aggregated, anonymized data from previously published studies; as such, this study does not constitute human subjects research according to U.S. Department of Health and Human Services guidelines (https://www.hhs.gov/ohrp/regulations-and-policy/guidance).

### Protocol registration

This research was developed by the authors and performed according to a protocol in which all the stages of the study were pre-defined. The protocol was recorded and published in the International Prospective Register of Systemic Reviews (PROSPERO) online database, number CRD42015026080, available at http://www.crd.york.ac.uk/PROSPERO/display_record.asp?ID=CRD42015026080. This study is reported in accordance with PRISMA guidelines (see attached checklist document, S1 File).

### Eligibility criteria

Studies that evaluated morbidities related to infection with *Schistosoma* species, before and after specific chemotherapy for schistosomiasis, were included in this review. In our quantitative meta-analysis, which focused on morbidity prevalence before and after chemotherapy, only morbidities reported by more than one study (from which the necessary data could be extracted) were included. No restrictions were placed in terms of location of the study, *Schistosoma* species, or publication date. Publications in English, Portuguese, Spanish, and French were included. We excluded animal studies, case studies, reviews, and studies with individuals selected only from clinics or hospitals. Regarding study design, any prospective, longitudinal studies of treatment impact on morbidity (with or without concurrent control group) were considered eligible for inclusion in the meta-analysis. Studies had to describe the study site, the species of *Schistosoma* parasite, the type of schistosomiasis morbidity evaluated before and after chemotherapy, the diagnostic method used to assess the morbidity, and the characteristics of participating study subject population. In addition, the numbers of subjects evaluated at baseline and at each follow-up were required, along with reporting of morbidity prevalence or mean laboratory values before and after treatment intervention.

### Information sources

The publications analyzed in this review were identified by searching public electronic databases including PubMed, and the Virtual Health Library VHL/BIREME (http://pesquisa.bvsalud.org/portal), which allows access to multiple databases (LILACS, MEDLINE and Cochrane Library), and Google Scholar (https://scholar.google.com/). The searches were conducted in August 2015. In addition, the bibliography reference lists of articles selected for review were evaluated for additional relevant studies, and additional articles were retrieved from personal collections at Case Western Reserve University.

### Search strategy

Published studies were identified in the electronic databases using the PICO strategy (Patient, Intervention, Comparator, and Outcome) to develop the descriptors. The descriptors used to identify patients were ‘Schistosomiasis’ and ‘*Schistosoma’*; for interventions, ‘drug therapy’, ‘treatment outcome’, and ‘therapeutics’; for outcomes, ‘morbidity’, ‘anemia/anaemia’, ‘pain’, ‘diarrhea’, ‘attention’, ‘memory’, ‘underachievement’, ‘growth’, ‘nutritional status’, ‘physical fitness’, ‘hydronephrosis’ ‘hematuria/haematuria’, ‘knowledge’, ‘work capacity evaluation’, ‘body weight’, ‘hepatomegaly’, ‘splenomegaly’, ‘hypertension, portal’, ‘proteinuria’, ‘disability evaluation’, and ‘fibrosis’. These descriptors were taken from the terminology of classification systems for indexing each database, MeSH (Medical Subject Headings) and DeCS (Health Sciences Descriptors). In the VHL/BIREME database the descriptors were considered in three languages, English, Portuguese, and Spanish.

### Study selection

References obtained from each search were exported to reference manager software Mendeley (version 1.14). The selection of studies was carried out in two stages by two independent reviewers (GA and DJB), and in case of disagreement between them, a third reviewer (CHK) was asked to resolve differences. The first stage of selection analyzed the titles and abstracts of the publications. The selected studies had full texts recovered for the second stage of selection. For this step we designed a Microsoft Access database form, using the eligibility criteria as described, in order to assist in the archiving of eligible studies for the systematic review. Duplicate publications and papers reporting reanalysis of previously published data were excluded at this stage.

### Data collection process

The data abstracted from selected publications were curated in the Microsoft Access study database. The reviewers extracted the following information from each text: full citation, year of publication, country and region where the study was conducted, characteristics of subjects (age, sex, and selection criteria), *Schistosoma* species studied, type of treatment offered and dose, follow-up time in months, number of individuals evaluated, type of morbidity evaluated in the study, method used to measure morbidity, prevalence or mean for each morbidity measure, egg counts or reduction rate of eggs, and prevalence of infection in the population. Wherever possible, all of the information listed above was recorded both before and after intervention (see supplemental information in Tables A-K in S1 Text).

Studies that evaluated more than one form of morbidity were included in the meta-analysis for each individual morbidity outcome. Moreover, when a paper reported more than one study with the same morbidity (performed among different subjects), this publication was listed twice. Thus, the number of identified studies was higher than the number of publications, but each study was included in the quantitative analysis no more than once per morbidity.

### Definition of outcomes

The quantitative analysis of the data included: first, an analysis of the impact of treatment *per se* on the odds of having morbidity after therapy; and second, a separate analysis by meta-regression of the specific impact of egg count reductions on the odds of post-treatment disease. The following morbidities were associated in common with infection with either *S*. *mansoni*, *S*. *haematobium*, or *S*. *japonicum*: splenomegaly, hepatomegaly, and mean hemoglobin. For intestinal schistosomiasis caused by *S*. *mansoni* or *S*. *japonicum*, we included periportal fibrosis, diarrhea, blood in the stool, and alteration in the main portal vein. For urogenital infection with *S*. *haematobium*, we included hematuria, proteinuria, abnormalities in the urinary bladder, and lesions of the upper urinary tract. Additional outcomes that could not be evaluated quantitatively due to differences in methods and classification included anthropometric measures, oxygen consumption, tolerance to physical activity, and abdominal pain. [N.B. The meta-analysis of outcomes of cognitive performance and school achievement will be published in a separate paper.]

For hematuria, we only included studies that evaluated the microhematuria detected by reagent strips, whereas studies of hematuria detected by visual inspection were excluded. For morbidity studies that presented ordinal rankings of severity, such as ‘periportal fibrosis grades 1–3’, we classified morbidity as ‘present’ for individuals with any degree of severity. Thus, the decrease in prevalence after chemotherapy in this meta-analysis represent the complete reversal of morbidity. Partial reversal of morbidity, such as a shift from grade 3 to grade 2 as reported in some studies, was not considered.

For studies that evaluated the morbidities more than once after the treatment, the first follow-up after the intervention was selected for inclusion in the pooled analysis and calculation of summary estimates. Other follow-up periods were analyzed later in subgroup analysis. Regardless of the number of segments in the study, the change in morbidity was always assessed against pre-treatment baseline values.

### Quantitative meta-analysis

Quantitative pooled analysis of treatment effects catalogued from the eligible studies was performed using Comprehensive Meta-Analysis software, v.3.3 (CMA, Biostat, Englewood, NJ) which provided calculation of summary estimates of the impact of treatment, along with their confidence intervals. For morbidity reported as dichotomous outcomes, a pooled odds ratio was calculated with 95% confidence interval (CI_95%_) using Der Simonian and Laird random effects modeling. For continuous data, the measure of effect was the calculated standardized mean difference (SMD) and its CI_95%_. The Z-test was used to assess statistical significance at a *P* < 0.05 level. For each morbidity, summary data were presented visually by Forest plots showing the respective odds ratio or SMD and CI_95%_ for the pooled analysis. (Additional data from subgroup analyses are shown in tables of effect size in this paper’s supplemental file, see Tables A-K in S3 Text).

### Meta-regression

For meta-regression of the impact of reduction in infection intensity after treatment, the egg reduction rate (ERR), for eggs detected on standard stool or urine diagnostic testing was calculated by the formula:

$$ERR=\frac{mean\;egg\;count\;at\;baseline-mean\;egg\;count\;at\;follow\;up}{mean\;egg\;count\;at\;baseline}*100$$

The ERR was initially calculated using either the geometric mean (ERR_GM_) or arithmetic mean (ERR_AM_) egg counts, depending on the data provided by the study. Twenty-six studies reported geometric mean data outcomes, while 12 reported arithmetic means. For consistency in our meta-regression of ERR *vs*. logarithmically transformed odds ratios, we converted the ERR_AM_ values to estimated ERR_GM_ values using correlations developed by Olliaro, et al., [30] in their systematic review of treatment effects on individual egg count values. The objective of this meta-regression was to assess the impact of treatment on morbidity according to the intensity reduction across the range of included studies. The percent reductions in log OR that we have projected for a 90% ERR are derived from the correlation coefficients and their CIs. Conversion of the estimated log_10_(OR) at 90% ERR to its corresponding OR by exponentiation yielded a fraction projected as the remaining odds of morbidity at that ERR value.

### Quality assessment and risk of study bias

The perceived quality of individual studies was assessed, but not formally quantified in our analysis because of specific limiting features found in many NTD trials [31]. A summary of study design and quality factors for each included study is presented in supplemental information file S2 Text: ‘Study design and quality features for included studies’. We assessed study quality using the National Heart, Lung, and Blood Institute quality assessment tools for pre-post design studies (https://www.nhlbi.nih.gov/health-pro/guidelines) with one additional criterion about reporting of treatment coverage. Most studies worked with endemic populations living in small clusters and so did not select an entire population or a randomly-selected subsample to follow. In addition, many older studies did not detail their criteria for selection of the population. For our analysis, it was assumed that the included studies represented the best available information for the population and morbidity of interest at the time it was undertaken.

### Publication bias

Assessment for potential publication bias was carried out by visual inspection of funnel plots, and statistically by calculating the Egger test [32].

### Heterogeneity and sensitivity analysis

Heterogeneity among studies in each meta-analysis was assessed using the Cochrane Q test (χ^2^ test) with significance assumed for P < 0.1, and Higgin's and Thompson's I^2^ statistic [33]. To explore heterogeneity and factors that could potentially modify the summary estimates of effect, we performed subgroup analyses stratified by parasite species, the study area, age of the subjects included in the studies, the time to follow-up after treatment, the type of diagnosis, the treatment performed, the number of treatments, and the initial prevalence of infection in the study population [34]. Not all morbidities had such stratifying data for all studies. For the sensitivity analysis, each meta-analysis was retested with the exclusion of one study at a time to assess the possibility of a disproportionate impact of any individual study on summary estimates.

## Results

### Study selection

**Fig 1:**
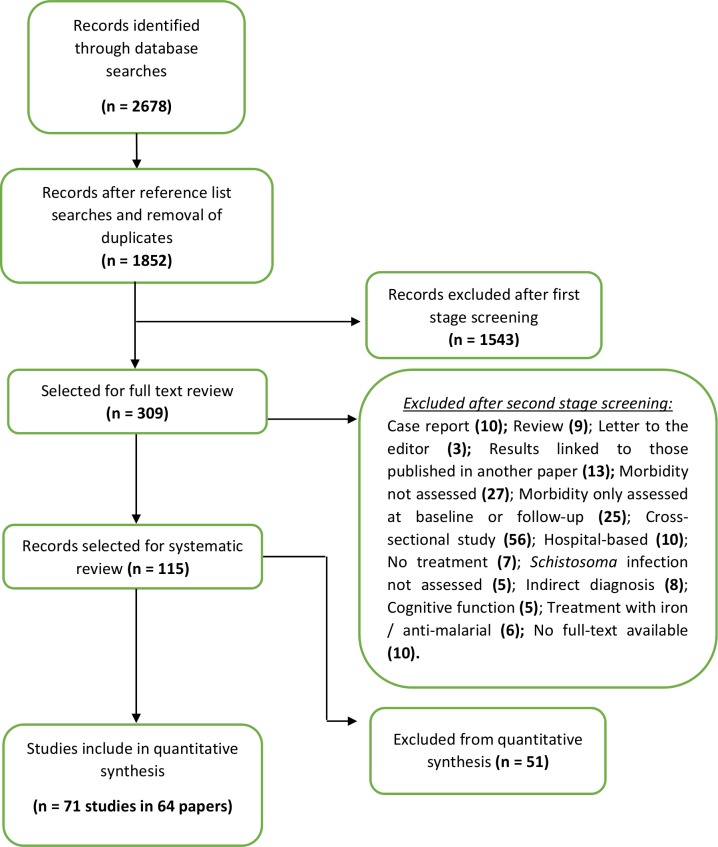
Flow chart of study search and selection strategy. The flow diagram indicates the numbers of titles and studies reviewed in preparation of the current systematic review and meta-analysis of chemotherapy treatment effects on infection-related morbidities in *Schistosoma*-endemic areas.

Using the selected search terms, initial screening of the databases yielded 1852 study reports after removing duplicates. After titles and abstracts were assessed, 309 reports were selected for full review. Publications eliminated in the first stage were excluded because they were animal studies, review studies, case reports, immunological studies, studies of parasitological efficacy and safety only, diagnostic studies, reinfection studies, spatial distribution studies, or evaluations of mass treatment programs, surgical intervention, other diseases, prevalence of coinfection, or studies to estimate prevalence and intensity of infection. As outlined in **Fig 1**, 194 of these study reports were excluded after second stage screening, leaving a total of 115 reports for inclusion in the systematic review. However, 51/115 papers did not have sufficient quantitative data on morbidity or on subject characteristics, or had different data formats, such that a final total of 64 papers (see S1 Text) were ultimately included in the quantitative data synthesis (meta-analysis) presented in this report.

### Study characteristics

Seventy-one eligible studies were abstracted from sixty-four papers. Publication dates ranged from 1977 to 2013 (median year = 1996). Studies were conducted in twenty-one countries. Of the seventy-one studies, 51% were from East Africa (Kenya, Tanzania, Madagascar, Ethiopia, Burundi, Uganda), 18% were from West Africa (Ghana, Niger, Mali, Senegal, Burkina Faso, Côte d’Ivoire), 7% were from Southern Africa (Zambia and Zimbabwe), 8.5% were from South America or the Caribbean (Brazil, Venezuela, and St. Lucia), 5.5% were from China, 5.5% were from Sudan, and there was one study each from Central Africa (Congo), from Indonesia, and the Philippines. The greatest number of subjects evaluated for morbidity outcomes had *S*. *haematobium* infections (52%), followed by *S*. *mansoni* (38%), *S*. *japonicum* (8.5%) and mixed infections (1.5%). Most of the subjects were school-age individuals (45%), but some studies included subjects of all ages (34%), whereas 21% were in studies that selected their subjects according to sex, age, clinical status, or presence of comorbidities such as hookworm. Most studies (82%) used praziquantel as the specific treatment for schistosomiasis, and 70% used a PZQ dose of 40 milligrams per kilogram. 8.5% used metrifonate, 6% used oxamniquine, while 4% used hycanthone. Moreover, 14% of studies used some of combination therapy with mebendazole or albendazole for treatment of intestinal helminths. Overall, the studies enrolled a total of 24,214 subjects at baseline and 22,207 individuals were monitored for morbidity outcomes (considering the first follow-up of each study). We found no evidence of publication bias using unweighted, non-randomized values in the Egger test.

### Impact of treatment on selected morbidities

#### Hepatomegaly

**Fig 2:**
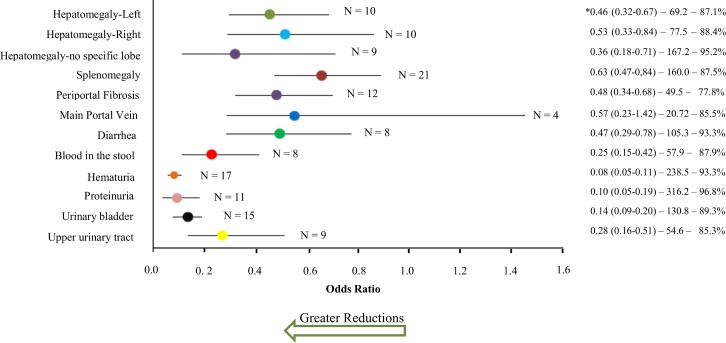
Odds ratios for morbidities related to schistosomiasis after treatment. Color circles indicate summary odds ratios estimated by random effects meta-analysis for morbidity prevalences after treatment, as compared to pre-treatment levels. The (N) by each line indicates the number of studies included in that meta-analysis. *These numbers indicate, respectively, the odds ratio and its 95% confidence interval, the Cochran χ^2^ value (where all χ^2^ values had *P* < 0.001), and Higgin's and Thompson's *I*^2^ statistic for heterogeneity estimation among the studies in each meta-analysis. Corresponding Forest plots of included studies and their summary statistics are included in Supplemental Information file S1 Fig.

Of the studies that assessed the impact of chemotherapy on reducing hepatomegaly, 10 evaluated the reduction of the left hepatic lobe, 10 the reduction of the right hepatic lobe, and 9 studies reported a reduction from the costal margin without specifying the lobe (see Table A in S1 Text). Overall, for each of the three types of outcome, the population surveyed experienced a significant reduction in the odds of hepatomegaly after treatment, compared to pre-treatment levels. In the overall analysis, the odds of having the left hepatic lobe enlarged was reduced significantly (OR 0.47, CI_95%_ 0.33, 0.67) after intervention **(Fig 2)**. Subgroup analysis indicated that studies with follow-ups done greater than 24 months after treatment and studies employing ultrasound for the diagnosis of hepatomegaly did not show statistically significant reductions in hepatomegaly (see Table A in S3 Text).

Across all studies, the aggregate odds of having the right hepatic lobe enlarged was reduced significantly (OR 0.53, CI_95%_ 0.34, 0.84) after treatment **(Fig 2).** However, among population studies that included all age groups, reductions in rates of right-sided hepatomegaly were not statistically significant (Table A in S3 Text). Reductions in right-sided hepatomegaly were also not significant for studies having a follow-up shorter than 12 months, or for individuals with *S*. *japonicum* or *S*. *haematobium* infections, and consequently, in the regions of China and East Africa. Studies in which the initial prevalence of infection was 100% (i.e., selected populations studies) also did not yield significant reductions when pooled.

Overall, there was a significant reduction in the odds of ‘hepatomegaly’ (without the lobe being specified) after treatment (OR 0.37, CI_95%_ 0.19, 0.71) **(Fig 2)** but this effect was not consistent in studies performed in South America or among studies that included subjects of all ages (Table A in S3 Text). Four main factors were associated with greater treatment impact in reducing hepatomegaly: use of school age populations, infection with *S*. *mansoni*, post-treatment follow-up greater than 24 months (except for the left hepatic lobe), and studies in South Africa. There was significant heterogeneity among the studies in the three types of evaluation **(Fig 2)**, that was not significantly reduced by subgroup analyses (see Table A in S3 Text). To test for outliers or the effects of larger influential studies in our analysis, sensitivity analysis by exclusion of one study at a time from the meta-analysis did not affect the outcome performance of the odds ratio, Z, and p-values, and the significance of observed associations did not change (S2–S4 Figs).

#### Splenomegaly

Spleen size was evaluated before and after chemotherapy for schistosomiasis in 21 studies (see Table B in S1 Text). Overall, there was a significant reduction in the odds of splenomegaly after treatment intervention (OR 0.63, CI_95%_ 0.47, 0.85) **(Fig 2)**. Sensitivity analysis by subgroup showed that studies that used ultrasound for diagnosis, studies with only school age individuals, studies having follow-up less than 12 months after treatment, studies of individuals with *S*. *japonicum* and *S*. *haematobium* infection, and studies having only infected subjects, showed no significant treatment impact when grouped. Reduction in splenomegaly was significantly greater among subjects who were followed after the first year of treatment, in the South Africa region, and among individuals selected for the presence of specific morbidities (see Table B in S3 Text). Significant heterogeneity was observed among the included studies **(Fig 2),** which decreased somewhat when grouped among the studies performed in South Africa and in the subgroup with individuals selected for some specific morbidity (Table B in S3 Text). Besides that, when the study of Gryseels, et al., [35] was removed from the subgroup of studies with follow-up greater than 24 months, the significance of the summary OR increased and heterogeneity decreased substantially (OR 0.32, CI_95%_ 0.17, 0.59; I^2^ = 42.4%). Sensitivity analysis by exclusion of one single study at a time from the meta-analysis did not affect the results (S5 Fig). In the sensitivity analysis, the estimated splenomegaly reduction effects ranged from OR 0.59 to 0.70.

#### Periportal fibrosis

The reduction of fibrosis prevalence was measured in 12 studies (see Table C in S1 Text) that found, overall, a significant reduction in the odds of fibrosis after chemotherapy compared to pretreatment levels (OR 0.49, CI_95%_ 0.35, 0.69). Significant heterogeneity was observed among the studies included **(Fig 2)** and changed little following subgroup stratification. In subgroup analysis (Table C in S3 Text), there was no statistically significant treatment-related reductions among individuals infected with *S*. *japonicum* (and thus the studies conducted in China), or among studies from the region of Egypt and Sudan. Three main factors had the greatest association with reductions of periportal fibrosis: studies of individuals selected for having periportal fibrosis or hepatomegaly/splenomegaly at baseline, a follow-up period greater than 24 months, and studies from East Africa or South America (Table C in S3 Text). Sensitivity analysis by exclusion of a single study at a time from the meta-analysis did not affect the results. The sensitivity testing indicated that the reduction in post treatment odds of periportal fibrosis likely varied between OR 0.43 and 0.50 (S6 Fig).

#### Main portal vein

Four studies assessed the prevalence of portal vein dilation before and after chemotherapy for schistosomiasis (see Table D in S1 Text). When all studies were considered together, no significant reduction in post treatment prevalence (compared to pre-treatment prevalence) was found (OR 0.58, CI_95%_ 0.23, 1.42). In subgroup analysis (see Table D in S3 Text), we observed statistical significance when we combined those studies characterized by having two chemotherapeutic interventions performed in the study population, and those having a follow-up time greater than 24 months. A study that followed only school children [21] reported an important reduction in the prevalence of portal vein dilation after treatment (Table D in S1 Text). Among all the studies in this category, significant heterogeneity was observed **(Fig 2),** but this was reduced in the stratification by subgroups (Table D in S3 Text). Sensitivity analysis by exclusion of a single study at a time from the meta-analysis did not affect the results (S7 Fig).

#### Diarrhea

Eight studies evaluated the cessation of episodes of diarrhea after anti-schistosomal chemotherapy (see Table E in S1 Text). When considering all of these studies, there was significant reduction in the odds of having diarrhea after the intervention (OR 0.48, CI_95%_ 0.29, 0.79). In subgroup analysis (see Table E in S3 Text), we chose to redo analysis without the Zhao, et al. study [36], as it was the only study with *S*. *japonicum* infection and presented very discrepant results from studies with *S*. *mansoni*. Greater reductions were observed among individuals of school age and in studies that followed only people with known schistosomiasis at baseline. This was in contrast with the reduction among the studies where the initial prevalence of infection was not 100% (Table E in S3 Text). Significant heterogeneity was observed among the studies included **(Fig 2)** which could be reduced by subgroup stratification according to region, age, and follow-up time (Table E in S3 Text). Sensitivity analysis by exclusion of one study at a time from the meta-analysis did not affect the results (S8 Fig).

#### Blood in the stool

Eight studies evaluated the prevalence of blood in the stool (see Table F in S1 Text) and the meta-analysis summary estimate indicated a significant reduction after chemotherapy for schistosomiasis (OR 0.26, CI_95%_ 0.16, 0.42). Significant heterogeneity was observed among the studies included **(Fig 2)** which could be modified by subgroup stratification according to region, age, and time of follow-up. (see Table F in S3 Text). The reduced odds of blood in the stool post-treatment were similar in different subgroups. Sensitivity analysis by exclusion of one study at a time from the meta-analysis did not affect the results (see S9 Fig). However, the exclusion of one study with *S*. *japonicum* [36] and the study with preschoolers [37] increased the strength of association (S9 Fig).

#### Blood in the urine

The presence of microhematuria was evaluated in 17 studies (see Table G in S1 Text). The reduction in prevalence was highly significant after chemotherapy for *S*. *haematobium* when pooled across all studies (OR 0.08, CI_95%_ 0.05, 0.12). Significant heterogeneity was observed among the studies **(Fig 2)** and the subgroup analysis was performed in order to identify the causes (see Table G in S3 Text). Reduction of heterogeneity was observed, but heterogeneity remained high among studies that included the entire population, studies that included only infected individuals, studies with follow-up greater than 12 months, and studies performed in West Africa. There was greater strength of association when studies included only individuals of school age or when the follow-up was conducted in the first six months after treatment (Table G in S3 Text). Sensitivity analysis by exclusion of a single study at a time from the meta-analysis did not affect the results (S10 Fig).

#### Protein in the urine

The presence of protein in urine was measured in 12 studies (see Table H in S1 Text) and the reduction of its prevalence was highly significant (OR 0.10, CI_95%_ 0.05, 0.20) but slightly smaller than the reduction in odds for hematuria **(Fig 2)**. Significant heterogeneity was observed among the studies, which the subgroup analysis did not change significantly (see Table H in S3 Text). The association between treatment and proteinuria reduction was insignificant only when the studies that grouped egg negative individuals together with egg positive individuals were analyzed. (Table H in S3 Text). Sensitivity analysis by exclusion of a single study at a time from the meta-analysis did not affect the results (S11 Fig).

#### Abnormalities in the urinary bladder detected on ultrasound examination

Reversal of urinary bladder lesions was evaluated in 15 studies (see Table I in S1 Text). Meta-analysis summary estimates indicated a significant reduction after chemotherapy compared to pretreatment levels (OR 0.14, CI_95%_ 0.095, 0.21) **(Fig 2)**. Significant heterogeneity was observed among the studies, which the subgroup analysis did not change (see Table I in S3 Text). Three main factors yielded a larger estimate of the impact of therapy in reducing bladder lesions: i) when only subjects with existing pathology in the urinary tract at baseline were evaluated, ii) when the initial prevalence of infection in the subjects was 100%, and iii) when the follow-up was performed in the first 6 months after the treatment. As the time of follow-up increased, the chances of reversal of lesions decreased (Table I in S3 Text). Sensitivity analysis by exclusion of one study at a time from the meta-analysis did not affect the results (S12 Fig).

#### Abnormalities in the upper urinary tract

Nine studies assessed the reversal of lesions in the upper urinary tract (see Table J in S1 Text). Meta-analysis summary estimates indicated a significant reduction in upper urinary lesions after chemotherapy for schistosomiasis (OR 0.29, CI_95%_ 0.16–0.51). Significant heterogeneity was observed among the studies **(Fig 2)** and subgroup analysis (see Table J in S3 Text) identified reduced heterogeneity among the studies that included an entire population, or studies where the follow-up time was less than six months, and among studies performed in West Africa. The strength of association was higher as the follow-up time increased, unlike the results found in the lower urinary tract (Table J in S3 Text). Sensitivity analysis by exclusion of one study at a time from the meta-analysis did not affect the results (S13 Fig).

#### Hemoglobin

**Fig 3:**
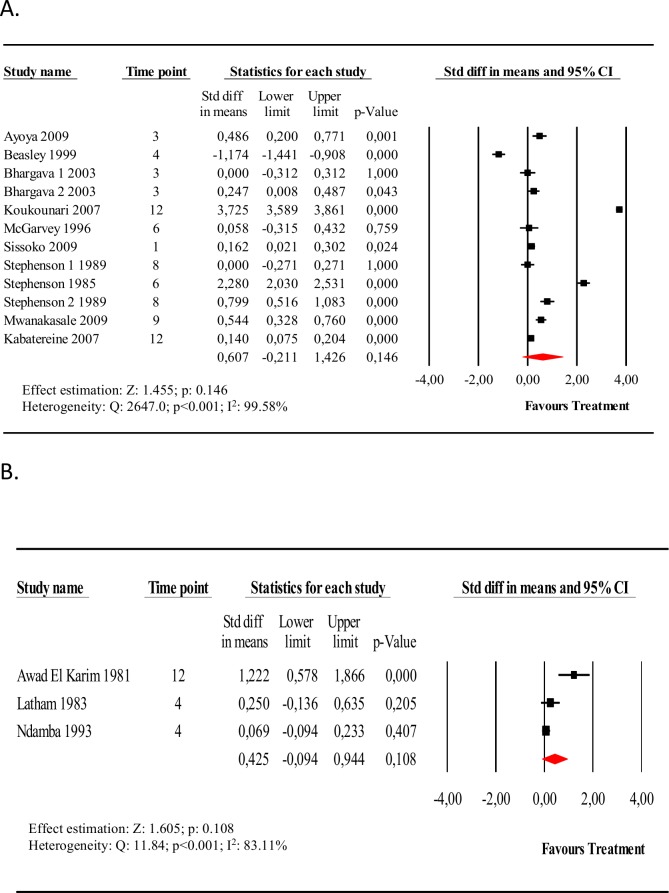
Forest plot of the effect of anti-schistosomal treatment on hemoglobin levels. Panel A, Forest plot and meta-analysis for the difference in mean hemoglobin levels, pre-intervention vs. post-intervention, for school-age subjects. Panel B, Forest plot and meta-analysis for the difference in mean hemoglobin levels, pre-intervention vs. post-intervention, for adult males.

Fifteen studies evaluated circulating blood hemoglobin levels before and after specific chemotherapy for schistosomiasis (see Table K in S1 Text). Thirteen studies evaluated school age children and three evaluated adult males. The hemoglobin level was higher after chemotherapy for schistosomiasis in nearly every study, however, the magnitude of this difference was modest (< 1 gm/dL) and the change was not found to be statistically significant. When considering only school-age subjects, the difference in the mean hemoglobin levels (pre-intervention vs. post-intervention) was 0.60 g/dL (CI_95%_ -0.2, 1.42) **(Fig 3, Panel A)**. Significant heterogeneity was observed among the studies and the subgroup analysis did not change heterogeneity significantly (see Table K in S3 Text). It was only when we pooled three studies with follow-up greater than 12 months after intervention that the mean post-treatment hemoglobin level was found to be significantly higher compared with pretreatment. Studies that combined treatment for schistosomiasis with another anthelmintic (mebendazole or albendazole) and studies using capillary blood for diagnosis found higher mean differences post-treatment, but these also did not reach statistical significance (Table K in S3 Text). Sensitivity analysis by exclusion of a single study at a time from the meta-analysis did not affect the results (S14 Fig). However, the exclusion of two studies with *S*. *haematobium* [15, 38] reduced the difference of the overall means and deleting the Beasley, et al. study [39] that evaluated children co-infected with hookworm increased the estimates of impact on mean hemoglobin post treatment. The meta-analysis of the three studies that included only adult males **(Fig 3, Panel B)** also found an increase in average hemoglobin from before to after the intervention, but this difference was not statistically significant, SMD 0.42 g/dL (CI_95%_ -0.09, 0.94).

### Associations between egg reduction rate and treatment-related reductions in morbidity level

To examine the hypothesis that post-treatment intensity of *Schistosoma* infection remains a correlate of morbidity risk after therapy, we performed meta-regression of the odds of having infection-related morbidities post-treatment as a function of post-treatment ERR achieved in an individual study population. The ERR was measured as reductions in population mean intensity of infection from before to after treatment. The impact on morbidity was measured as the corresponding change in morbidity prevalence, comparing the study population’s odds of disease before and after treatment.

**Fig 4:**
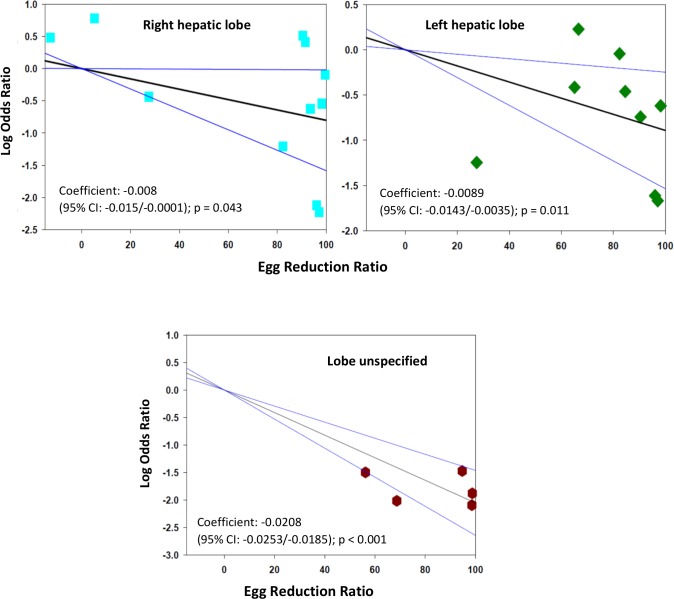
Log odds ratio for post-treatment hepatomegaly, according to egg reduction rates achieved, by hepatic lobe. The figure panels show the log10 of the post-treatment/pre-treatment odds ratio of morbidity, according to each study’s treatment mediated egg reduction rate (ERR), as related to hepatomegaly in the right lobe (upper left panel), n = 11, in the left lobe (upper right panel), n = 9, or where the lobe was not specified (lower panel), n = 5. **N.B**. the very low ERRs (< 0 to 20%) reflect observed post-treatment intensity outcomes seen among placebo-treated subgroups.

**Fig 5:**
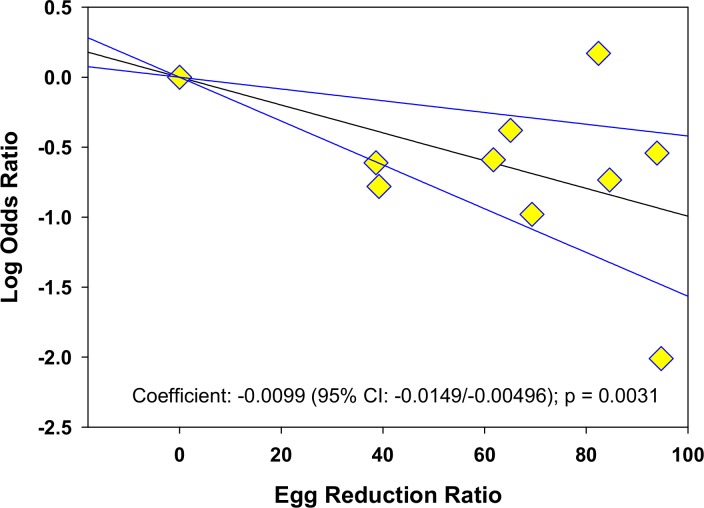
Log odds ratio of periportal fibrosis according to post-treatment egg reduction rate. The graph shows the log10 of the post-treatment/pre-treatment odds ratio for periportal fibrosis according to each study’s post-treatment egg reduction rate (ERR), n = 10.

**Fig 6:**
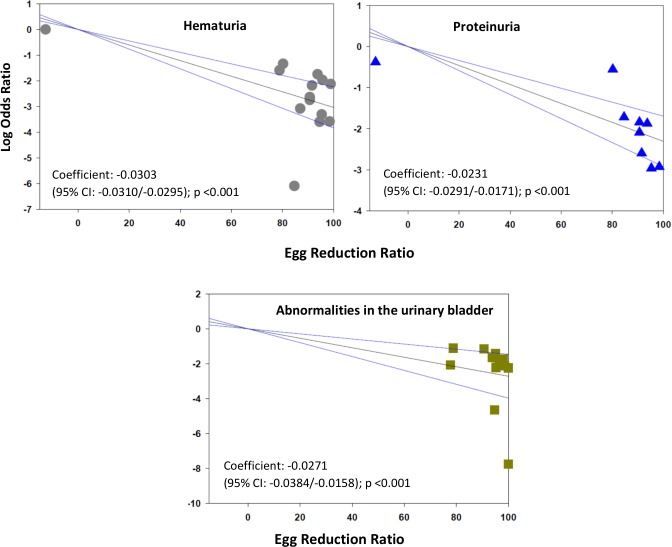
Log odds ratio of hematuria, proteinuria, and bladder abnormalities according to post-treatment egg reduction rate. The panels show the log10 of the post-treatment/pre-treatment odds ratio of morbidity according to post-treatment egg reduction rate (ERR) as related to hematuria (upper left panel), n = 14, proteinuria (upper right panel), n = 9, or urinary bladder abnormalities detected on ultrasound (lower panel), n = 12.

The meta-regression analysis suggested that there is a significantly greater reduction in the prevalence of some morbidities if greater egg reduction effects can be achieved. For hepatomegaly, a practical target of 90% egg reduction was projected to yield an estimated 84% (CI_95%_ 52%, 95%) reduction in the odds of left lobe enlargement. The corresponding projected reduction in the odds of right lobe enlargement was 81% (CI_95%_ 2%, 96%) while for unspecified lobar enlargement, the projected reduction in odds was 99% (CI_95%_ 98%, 99.4%) **(Fig 4)**. With respect to periportal fibrosis, a ninety-point ERR was predicted to reduce odds of this form of disease by 87% (CI_95%_ 64%, 95%) **(Fig 5)**. Greater ERR impact was projected for all morbidities related to urogenital schistosomiasis: a ninety-point egg reduction was predicted to yield reductions in the odds of hematuria by 99.8% (CI_95%_ 99.7%, 99.9%), in the odds of proteinuria by 99.2% (CI_95%_ 97%, 99.8%), and in the odds of ultrasound abnormalities in the urinary tract by 99.2% (CI_95%_ 96%, 99,9%) **(Fig 6)**.

Nevertheless, it was observed that even near-total reduction in egg counts by drug treatment was unlikely to lead to complete reduction of all morbidity risk. Not shown, the meta-regression showed only non-significant correlation of ERRs with reductions in the study cohort prevalence of splenomegaly or with post-treatment increases in hemoglobin levels.

## Discussion

Quantification of the net changes in *Schistosoma* infection-associated morbidity prevalence, from before to after treatment, is one way to critically value the impact of drug-based control of schistosomiasis, which is the strategy currently recommended by WHO and other agencies [10]. Our systematic review and meta-analysis sought to summarize many decades of research on disease-related benefits of treatment for schistosomiasis. To do this, we catalogued treatment impact on eleven key morbidities linked to infection by any of the three major *Schistosoma* parasites of humans, *S*. *haematobium*, *S*. *mansoni*, and *S*. *japonicum*. Overall, our results suggest that drug treatment significantly reduces but does not eliminate these common pathologic consequences of *Schistosoma* infection, and that the odds of improvement are linked to the magnitude of treatment-related reductions in adult worm burden of parasitic infection.

Chances of post-treatment morbidity reductions were higher for morbidities related to urogenital schistosomiasis than for morbidities caused by intestinal schistosomiasis. For the included urogenital morbidities associated with infection by *S*. *haematobium*, the greatest reduction after treatment was for odds of having hematuria; the lowest reduction was for the odds of having upper urinary tract lesions detected by ultrasound, primarily characterized by hydronephrosis. The presence of blood in the urine is a well-accepted marker of *S*. *haematobium* infection and its presence is used as a mapping and screening tool for urogenital schistosomiasis in Africa [40, 41]. Although hematuria, proteinuria, and bladder abnormalities appear to respond quickly to anti-schistosomal therapy, in step with the ERRs achieved, the relatively smaller improvements in prevalence of hydronephrosis suggest that this form of morbidity is a more slowly resolving and sometimes irreversible form of urinary tract schistosomiasis. This phenomenon thus limits the overall impact of drug treatment in communities at high risk for *S*. *haematobium* infection [42–44]. Post-treatment reductions in the odds of morbidities related to intestinal infections (*S*. *mansoni* and *S*. *japonicum*) ranged from 37–74%. The least impact was for splenomegaly, whereas the largest observed decrease was for blood in the stool. Regarding measured impact on splenomegaly, it was not uncommon that the studies selected for meta-analysis involved subjects who were co-infected with other chronic pathogens, especially malaria, which could explain a lesser effect of anti-schistosomal therapy on splenomegaly after treatment [22, 36]. Other reviews of *Schistosoma*-related ultrasound morbidities have suggested that the regression of splenomegaly, while sometimes observed after anti-schistosomal therapy, is not specific enough to be used as an indicator for the regression of *Schistosoma*-associated disease. In their analysis, malaria was the main co-factor contributing to this effect [45]. In addition, like hydronephrosis in urogenital schistosomiasis, splenic enlargement in intestinal schistosomiasis is likely a marker of more severe and more prolonged chronic intestinal schistosomiasis, and it may be more difficult to achieve regression with late treatment [46, 47].

In the pooled analysis of treatment effects, the 74% reduction in odds of blood in the stool was the best result among morbidities associated with intestinal infection. Large -scale questionnaire surveys of blood in the stool, trialed as rapid assessment tools for identifying high-risk communities in sub-Saharan Africa, have shown that this symptom can be a valuable indicator for the diagnosis of *S*. *mansoni* in endemic areas, having low to moderate sensitivity and medium to high specificity [41]. For *S*. *japonicum* in China, a separate study has estimated that the highest risk indicator of infection-associated morbidity is a history of bloody stools [5]. Although regression in the odds of bloody stools was a quick indicator of anti-schistosomal treatment effect, the reductions were lower than for *S*. *haematobium*-associated hematuria, suggesting that this manifestation is less likely to be specific for intestinal schistosomiasis in the context of many other circulating enteropathogens.

In our subgroup analyses, some study features were clearly linked to either better or more limited reductions in morbidity prevalence after treatment. In many cases, more significant treatment effects were observed when studies were performed on school age children or on subpopulations selected for existing pathology at baseline. This was seen for the outcomes of hepatomegaly, diarrhea, periportal fibrosis, and abnormalities of the urinary bladder. In endemic regions, it is believed that age is an important proxy of cumulative exposure to the parasite and the related tissue damage that it causes. As the process of infection progresses from acute injury to a more chronic forms of fibrotic scarring, it becomes proportionately more difficult to reverse *Schistosoma*-associated pathology [44, 48]. In addition, the meta-analysis also suggests differences between *S*. *mansoni* and *S*. *japonicum* infections in their likelihood of morbidity reduction in response to therapy. Post-treatment odds of splenomegaly and periportal fibrosis were not significantly reduced for infection with *S*. *japonicum*, although studies of *S*. *mansoni* treatment effects were able to demonstrate significant impact for these two morbidity markers. These findings were consonant with two earlier reviews that have highlighted the persistence of abnormalities caused by *S*. *japonicum* [7, 45].

The chances of observing reductions in hepatomegaly, diarrhea, proteinuria, and bladder abnormalities were higher when the studies were performed on subjects who were definitely infected, *i*.*e*., all egg-positive. However, this was not the case for splenomegaly reduction or for periportal fibrosis. Eggs are most consistently detected in stool or urine with heavier infections, and persons with light intensity infection may have morbidity but have egg-negative status on the day of survey testing. Those studies that included these egg-negative infections may have shown a greater impact on morbidities because of there being a proportionately greater impact of treatment on resident worm burden (with possible complete parasitological cure) in light infection.

Also in our analysis, follow-up interval was an important factor in gauging the impact of therapy. For those morbidities related to intestinal schistosomiasis, *i*.*e*. hepatomegaly, splenomegaly, and periportal fibrosis, a longer follow-up period, especially > 24 months, was associated with greater reductions after treatment. The exception was left hepatic lobe enlargement, which had the best reductions in the first year after treatment, but decreased benefit over longer time periods. Of note, the reductions in morbidities associated with urogenital schistosomiasis, with the exception of injuries to the upper urinary tract, were more likely to be significant if evaluated in the first six months after treatment.

In our summary estimates, only two morbidities showed no consistent or significant change between pre- and post-treatment surveys. These were the prevalence of portal vein dilation and change in mean hemoglobin level. Only studies delivering two chemotherapeutic interventions and those having a follow-up time greater than 24 months were associated with significant reductions in the diameter of the portal vein. In clinical studies, portal vein diameter is an indicator that correlates with portal vein pressure and risk for hemorrhage [47]. This finding likely reflects a more advanced stage of disease with a smaller chance of a beneficial chemotherapy effect from a single dose. With respect to hemoglobin levels, it was only possible to identify statistically significant changes when the follow-up was performed at an interval greater than twelve months after treatment. Prior analysis has indicated that the benefit in terms of gains in hemoglobin levels is greatest among those who have anemia at baseline, or those who have greater levels of microhematuria or infection intensity [15, 20]. Studies of *S*. *japonicum* have found that the peak elevation of post-treatment hemoglobin levels occurs at 15 months [16]. Of importance to public health, it appears that monitoring of schistosomiasis-associated anemia impact should be planned for a period at one year or more after treatment.

The relative intensity of infection is an important correlate of morbidity, because the formation of the disease is related to the daily deposition of parasite eggs into host tissues [17, 48–50]. While immediate granulomatous inflammation is the cause of some of the morbidities included in our review (hematuria, proteinuria, bladder irregularities for *S*. *haematobium*; bloody stool, diarrhea, and hepatic enlargement for *S*. *mansoni* and *S*. *japonicum*; and anemia of inflammation for all three species), cumulative damage over decades of infection is linked to advanced fibrotic complications of infection such as hydronephrosis, portal fibrosis, and portal dilation. Our meta-regression profiles indicate that acute reductions in worm burden, as reflected by the ERRs achieved after drug therapy, are associated with reversal of most of the acute pathologies of infection. However, the more advanced chronic forms of disease were less responsive to single rounds of treatment, even with adequate ERRs, and our stratified analysis suggests that multiple rounds of treatment are necessary to improve (or hopefully prevent) these outcomes.

As study limitations, there is moderate risk of bias in this study’s estimates. The data analyzed in this study may have been influenced by confounders such as uneven sex distributions, the presence of co-infections, and variation in local reinfection rates that could not be controlled for in the meta-analysis. Moreover, the evidence may be limited in terms of generalizability because of the limitations in the design of included studies, and because the diverse populations selected for analysis yielded a high degree of heterogeneity across studies. To help minimize these effects, we have used random effects modeling in the meta-analysis and have performed sensitivity analysis to look for possible skewing of estimates by results from single influential studies [34].

Our meta-analysis identified that significant gaps exist in the available literature on post-treatment reduction of morbidities. In our study’s quality assessment, the study factors that most frequently could not be evaluated were: subject inclusion/exclusion criteria, the power analysis of the selected study sample size, and the use of blinding for assessment of study outcomes. Loss to follow-up was > 20% from baseline in many studies, and the potential biasing effect of this phenomenon was often not considered. Meta-analysis and meta-regression are observational research that depends on the quality of the studies that are included. As previously noted by others [31], the level of evidence for many NTD clinical studies has to be categorized as only “very low, low, or moderate quality”. That said, is has been the chronic underfunding of clinical trials (performed in resource limited settings) that has been an important part of the ‘neglect’ of NTDs. In order to strengthen the evidence base for *Schistosoma* morbidity control, there is a clear need to perform additional cohort trials that are both well-designed and well-reported.

The main findings of this meta-analysis are: i) post-treatment reduction in morbidity varies according to *Schistosoma* species; ii) for most pathologies, the odds of persisting morbidity progressively decrease with greater reductions in post-treatment egg counts (ERR); iii) however, not all morbidities respond in parallel with egg reduction. The population studied, their ages and infection status, and the interval for follow-up all influenced the magnitude of morbidity reductions noted in a given study cohort. Our findings illuminate and help to quantify the magnitude of improvements after treatment of *Schistosoma*-associated morbidities. These new estimates may prove useful in cost-effectiveness estimations for program planning, and can provide direction for future operational research on treatment implementation strategies.

## Supporting information

S1 File PRISMA checklist. (DOC)

S2 File PROSPERO protocol CRD42015026080 used for this study. (PDF)

S1 Text Tables A-K indicating the main characteristics of included studies evaluating the impact of chemotherapy on different *Schistosoma* infection-related morbidities.
(PDF)

S2 Text A table describing study design and quality features for included studies. (PDF)

S3 Text Tables A-K indicating the results of subgroup analysis of pre- and post-treatment morbidity prevalence.
(PDF)

S1 Fig Forest plots of post-treatment odds ratios of *Schistosoma*-associated morbidities included in this paper. Individual plots indicate, by morbidity, the results for each individual study included for analysis, and the summary OR and confidence interval estimated across all included studies. ORs and their confidence intervals for individual studies are shown numerically in the statistics columns, and graphically by the corresponding black boxes and black lines. The summary OR and confidence interval is indicated by the red diamond at the bottom of each plot.
(PDF)

S2 Fig Sensitivity analysis Forest Plot of the impact of therapy on left lobe hepatomegaly prevalence. Forest plot showing sensitivity analysis, performed by removing one study at a time, for the effect of treatment on prevalence of left hepatic lobe hepatomegaly.
(PDF)

S3 Fig Sensitivity analysis Forest Plot of the impact of therapy on right lobe hepatomegaly prevalence. Forest plot showing sensitivity analysis, performed by removing one study at a time, for the effect of treatment on prevalence of right hepatic lobe hepatomegaly.
(PDF)

S4 Fig Sensitivity analysis Forest Plot of the impact of therapy on hepatomegaly prevalence, lobe unspecified. Forest plot showing sensitivity analysis, performed by removing one study at a time, for the effect of treatment on prevalence of hepatomegaly (lobe not specified).
(PDF)

S5 Fig Sensitivity analysis Forest Plot of the impact of therapy on splenomegaly prevalence. Forest plot showing sensitivity analysis, performed by removing one study at a time, for the effect of treatment on prevalence of splenomegaly.
(PDF)

S6 Fig Sensitivity analysis Forest Plot of the impact of therapy on periportal fibrosis prevalence. Forest plot showing sensitivity analysis, performed by removing one study at a time, for the effect of treatment on prevalence of periportal fibrosis
(PDF)

S7 Fig Sensitivity analysis Forest Plot of the impact of therapy on portal vein dilation prevalence. Forest plot showing sensitivity analysis, performed by removing one study at a time, for the effect of treatment on prevalence of portal vein dilation.
(PDF)

S8 Fig Sensitivity analysis Forest Plot of the impact of therapy on diarrhea prevalence. Forest plot showing sensitivity analysis, performed by removing one study at a time, for the effect of treatment on prevalence of diarrhea after treatment.
(PDF)

S9 Fig Sensitivity analysis Forest Plot of the impact of therapy on blood in stool prevalence. Forest plot showing sensitivity analysis, performed by removing one study at a time, for the effect of treatment on prevalence of blood in stool.
(PDF)

S10 Fig Sensitivity analysis Forest Plot of the impact of therapy on hematuria prevalence. Forest plot showing sensitivity analysis, performed by removing one study at a time, for the effect of treatment on prevalence of blood in urine.
(PDF)

S11 Fig Sensitivity analysis Forest Plot of the impact of therapy on proteinuria prevalence. Forest plot showing sensitivity analysis, performed by removing one study at a time, for the effect of treatment on prevalence of protein in urine.
(PDF)

S12 Fig Sensitivity analysis Forest Plot of the impact of therapy on urinary bladder abnormalities prevalence. Forest plot showing sensitivity analysis, performed by removing one study at a time, for the effect of treatment on prevalence of ultrasound abnormalities of the urinary bladder.
(PDF)

S13 Fig Sensitivity analysis Forest Plot of the impact of therapy on urinary tract abnormality prevalence. Forest plot showing sensitivity analysis, performed by removing one study at a time, for the effect of treatment on prevalence of ultrasound abnormalities in the upper urinary tract.
(PDF)

S14 Fig Sensitivity analysis Forest Plot of the impact of therapy on blood hemoglobin levels. Forest plot showing sensitivity analysis, performed by removing one study at a time, for the effect of treatment on blood hemoglobin.
(PDF)
