# Supplementary material for: Decline in infection-related morbidities following drug-mediated reductions in the intensity of Schistosoma infection: A systematic review and meta-analysis
Source: PLoS Negl Trop Dis. 2017 Feb 17;11(2):e0005372. doi: 10.1371/journal.pntd.0005372 (PMC5333910; doi:10.1371/journal.pntd.0005372)
Supplement: S2 File — (PDF) [file pntd.0005372.s002.pdf]

PROSPERO International prospective register of systematic reviews

Review title and timescale

1 Review title

Give the working title of the review. This must be in English. Ideally it should state succinctly the interventions or exposures being reviewed and the associated health or social problem being addressed in the review.

Reduction in the intensity of Schistosoma infection after treatment and its effect on morbidity related to infection (schistosomiasis): a meta-analysis

2 Original language title

For reviews in languages other than English, this field should be used to enter the title in the language of the review. This will be displayed together with the English language title.

3 Anticipated or actual start date

Give the date when the systematic review commenced, or is expected to commence.

25/08/2015

4 Anticipated completion date

Give the date by which the review is expected to be completed.

01/07/2016

5 Stage of review at time of this submission

Indicate the stage of progress of the review by ticking the relevant boxes. Reviews that have progressed beyond the point of completing data extraction at the time of initial registration are not eligible for inclusion in PROSPERO. This field should be updated when any amendments are made to a published record.

The review has not yet started ×

Review stage	Started	Completed
Preliminary searches	Yes	Yes
Piloting of the study selection process	Yes	Yes
Formal screening of search results against eligibility criteria	Yes	Yes
Data extraction	Yes	Yes
Risk of bias (quality) assessment	Yes	Yes
Data analysis	Yes	Yes

Provide any other relevant information about the stage of the review here.

Review team details

6 Named contact

The named contact acts as the guarantor for the accuracy of the information presented in the register record.

Gisele Andrade

7 Named contact email

Enter the electronic mail address of the named contact.

giseleunifal@hotmail.com

8 Named contact address

Enter the full postal address for the named contact.

Escola de Enfermagem da Universidade Federal de Minas Gerais Av. Alfredo Balena 190 sala 418 - Santa Efigenia 30130-100

9 Named contact phone number

Enter the telephone number for the named contact, including international dialing code.

+55 31 991664570

10 Organisational affiliation of the review

Full title of the organisational affiliations for this review, and website address if available. This field may be completed

as 'None' if the review is not affiliated to any organisation.
Nursing School - Federal University of Minas Gerais

Website address:

11 Review team members and their organisational affiliations

Give the title, first name and last name of all members of the team working directly on the review. Give the organisational affiliations of each member of the review team.

Title	First name	Last name	Affiliation
	Gisele	Andrade	Nursing School - Federal University of Minas Gerais
Dr	Andrea	Gazzinelli	Nursing School - Federal University of Minas Gerais
Dr	Charlie	King	Case Western Reserve University
	Dave	Bertsch	Case Western Reserve University

12 Funding sources/sponsors

Give details of the individuals, organizations, groups or other legal entities who take responsibility for initiating, managing, sponsoring and/or financing the review. Any unique identification numbers assigned to the review by the individuals or bodies listed should be included.

This study was supported by the Tropical Medicine Research Center, National Institutes of Health–TMRC-NIH (Grant Number P50AI098507), Fundação de Amparo à Pesquisa de Minas Gerais-FAPEMIG, Conselho Nacional de Desenvolvimento Científico e Tecnológico-CNPq, Coordenação de Aperfeiçoamento de Pessoal de Nível Superior-CAPES (RCO and ROP).

13 Conflicts of interest

List any conditions that could lead to actual or perceived undue influence on judgements concerning the main topic investigated in the review.

Are there any actual or potential conflicts of interest?

None known

14 Collaborators

Give the name, affiliation and role of any individuals or organisations who are working on the review but who are not listed as review team members.

Title	First name	Last name	Organisation details

Review methods

15 Review question(s)

State the question(s) to be addressed / review objectives. Please complete a separate box for each question.

How does egg reduction after treatment for Schistosoma infection translate into schistosomiasis morbidity reduction?

16 Searches

Give details of the sources to be searched, and any restrictions (e.g. language or publication period). The full search strategy is not required, but may be supplied as a link or attachment.

The selection of publications will be performed systematically at the electronic databases PubMed, Google Scholar, Web of Science, Scielo, African Journals Online Searching and BVS (Biblioteca Virtual em Saúde) in August and September of 2015. The search will be performed using combinations of the terms: “schistosomiasis”, “Drug Therapy”, “Treatment Outcome”, “disability”, “morbidity”, “anaemia”, “attention”, “development”, “cognition”, “memory”, school performance”, “work capacity”, “growth”, “weight”, “height”, “nutritional status”, “physical fitness”, “hepatosplenomegaly”, “periportal fibrosis”, “portal hypertension” “bladder deformity”, “haematuria”, “hydronephrosis”. The search strategy in Bireme (BVS) will consider these descriptors in English, Portuguese, and Spanish. The review will be expanded with the analysis of the reference lists of selected articles, by contact with researchers, and and review of congress publications, where possible. All studies published in English, Spanish, and Portuguese in which the morbidity and egg count was assessed before and after treatment will be included. There will be no date limit.

17 URL to search strategy

If you have one, give the link to your search strategy here. Alternatively you can e-mail this to PROSPERO and we will store and link to it.

I give permission for this file to be made publicly available
Yes

18 Condition or domain being studied

Give a short description of the disease, condition or healthcare domain being studied. This could include health and wellbeing outcomes.

Schistosomiasis is an infectious disease that affects more than 230 million people worldwide. Three main species of schistosomes infect human: Schistosoma haematobium, S. mansoni, and S. japonicum, occuring in Africa and the Middle East, in South America, and Asia. Adult schistosome worms can colonise the human body for years while excreting a hundreds to thousands of eggs each day. These eggs provoke granulomatous inflammation in order to achieve translocation from the venous circulation to either the bowel or bladder lumena. If they do not succeed in leaving the body in excreta, they remain trapped in nearby tissues, causing chronic inflammation and scarring. Evidence indicates that it is eggs that induce the bulk of morbidity caused by Schistosoma infection, and not the adult worms. Past studies of the morbidity related to schistosomiasis have focused on more advanced forms of organ pathology that, currently, are not very common. These include hepatosplenism, periportal fibrosis, bladder deformity, hydronephrosis, and bowel and bladder scarring. However, systemic morbidities such as anaemia, growth stunting, impaired cognition, undernutrition, diarrhoea, and decreased physical fitness are also related to Schistosoma infection. Given the number of people infected with Schistosoma blood flukes, these systemic morbidities may represent the more prevalent public health burden in endemic areas, and has being increasingly appreciated in recent times. From 1984, the World Health Organization has endorsed drug treatment (chemotherapy) as the main strategy to control morbidity caused by schistosomiasis. In 2006, it further advocated ‘preventive chemotherapy’ programs for school age children via mass treatments in endemic areas. These campaigns aim to reduce infectious burden and the morbidity related to Schistosoma infection, especially the more advanced forms of schistosomiasis. One way to reduce morbidity due to schistosomiasis is to reduce the intensity of infection, expressed in terms of a reduction in the number of eggs expelled per person (or the community average), through drug treatment intervention. Early cross-sectional community studies suggested a correlation between individual infection intensity and risk for schistosomiasis-related pathology. However, there is some evidence that just a post-treatment reduction in the intensity of the disease is not sufficient to reverse morbidity, given the fact that some morbidities can be found with all levels of infection intensity. In that regard, it is not clear whether controlling the intensity of infection alone is an effective way to control the morbidity related to schistosomiasis. The aim of this project is to critically review the available evidence on drug-based control of morbidity related to schistosomiasis, and to develop an evidence-based estimate of the impact of treatment and its consequent reduction in the intensity of infection in controlling morbidity related to schistosomiasis.

19 Participants/population

Give summary criteria for the participants or populations being studied by the review. The preferred format includes details of both inclusion and exclusion criteria.

The review will be conducted with papers that incorporate individuals infected with Schistosoma mansoni, S. Haematobium, or S. japonica who received treatment and had one or more of the designated forms of morbidity evaluated before and after treatment. Studies with animals will not be included.

20 Intervention(s), exposure(s)

Give full and clear descriptions of the nature of the interventions or the exposures to be reviewed

The exposure for effect that will be considered in this review will be the egg count reduction of the affected community after drug treatment. Minimum data should include Schistosoma species, type of drug treatment, treatment dose, measurement of morbidity/disability before and after treatment, and the treated population’s egg counts before and after treatment, or egg reduction rate.

21 Comparator(s)/control

Where relevant, give details of the alternatives against which the main subject/topic of the review will be compared (e.g. another intervention or a non-exposed control group).

1) Measurements of morbidity/disability and egg counts before treatment will be compared with those obtained after treatment; 2) Measurement of morbidity/disability and egg counts of people who received treatment will be compared with data of people who did not receive treatment and, therefore, had no substantial reduction in egg counts; 3) Subgroups with different characteristics such as initial infection intensity, age, gender, geographical location, treatment type, and dose.

22 Types of study to be included

Give details of the study designs to be included in the review. If there are no restrictions on the types of study design eligible for inclusion, this should be stated.

Both observational and clinical trial studies will be included in the review. No restrictions in terms of year. Studies will be evaluated studies in English, Spanish and Portuguese. Reviews and case studies will not be considered.

23 Context

Give summary details of the setting and other relevant characteristics which help define the inclusion or exclusion criteria.

24 Primary outcome(s)

Give the most important outcomes.

Rate of reduction of morbidity prevalence as related to the Schistosoma egg reduction rate (ERR) after treatment.

Give information on timing and effect measures, as appropriate.

25 Secondary outcomes

List any additional outcomes that will be addressed. If there are no secondary outcomes enter None.

Overall treatment program impacts on prevalences of morbidities associated with schistosomiasis (related and unrelated to ERR); Rate of reduction of morbidities by gender, sex, geographical location, initial infection intensity and treatment characteristics.

Give information on timing and effect measures, as appropriate.

26 Data extraction (selection and coding)

Give the procedure for selecting studies for the review and extracting data, including the number of researchers involved and how discrepancies will be resolved. List the data to be extracted.

After a systematic search, the papers identified will have their titles and abstracts reviewed by two trained reviewers, who will search for the data that point to the inclusion or exclusion of the article in this first step. A standardized form will be used as a guide for data capture and logging of entries. Data will be extracted on the egg counts before and after treatment, the morbidity that was evaluated in the study, also before and after treatment, other treatment details, study population characteristics, and characteristics of the study.

27 Risk of bias (quality) assessment

State whether and how risk of bias will be assessed, how the quality of individual studies will be assessed, and whether and how this will influence the planned synthesis.

As far as possible, the study quality and risk of bias will be evaluated. To likelihood of publication bias will be assessed using funnel plots and Egger's correlation statistic. To minimize bias in data collection, the present study will employ two independent reviewers and a standardized form to collect data. In case of disagreement a third reviewer will be consulted to resolve the issue. To evaluate the quality of the studies, we will use the CONSORT Checklist – Consolidated Satandards Reporting for Clinical Trials and the STROBE – Strengthening the Reporting of Observational Studies in Epidemiology. For each included study, we will have the items in the relevant checklists classified as yes or no, and the proportion of criteria satisfied will be enumerated for each paper.

28 Strategy for data synthesis

Give the planned general approach to be used, for example whether the data to be used will be aggregate or at the level of individual participants, and whether a quantitative or narrative (descriptive) synthesis is planned. Where appropriate a brief outline of analytic approach should be given.

The papers selected for the meta-analysis will have their data aggregated by morbidity outcome. We will calculate the mean difference, or frequency, or odds ratio before and after treatment for each morbidity, as related to egg reduction rate. After descriptive and narrative analysis of the data, results of the meta-analysis and comparison among studies will be presented using Forest plots with summary estimates of treatment effects. Heterogeneity analysis between the studies will also be performed.

29 Analysis of subgroups or subsets

Give any planned exploration of subgroups or subsets within the review. 'None planned' is a valid response if no subgroup analyses are planned.

Subgroup assessment will be used to explore causes for heterogeneity. Analysis of subgroups of age, gender, geographical location, initial infection intensity, treatment characteristics and follow-up will be performed.

Review general information

30 Type and method of review

Select the type of review and the review method from the drop down list.

Meta-analysis, Systematic review

Tropical Medicine

31 Language

Select the language(s) in which the review is being written and will be made available, from the drop down list. Use the control key to select more than one language.

English

Will a summary/abstract be made available in English?

Yes

32 Country

Select the country in which the review is being carried out from the drop down list. For multi-national collaborations select all the countries involved. Use the control key to select more than one country.

Brazil, United States of America

33 Other registration details

Give the name of any organisation where the systematic review title or protocol is registered together with any unique identification number assigned. If extracted data will be stored and made available through a repository such as the Systematic Review Data Repository (SRDR), details and a link should be included here.

34 Reference and/or URL for published protocol

Give the citation for the published protocol, if there is one.

Give the link to the published protocol, if there is one. This may be to an external site or to a protocol deposited with CRD in pdf format.

I give permission for this file to be made publicly available

Yes

35 Dissemination plans

Give brief details of plans for communicating essential messages from the review to the appropriate audiences.

Do you intend to publish the review on completion?

Yes

36 Keywords

Give words or phrases that best describe the review. (One word per box, create a new box for each term)

Schistosomiasis

morbidity

disability

egg reduction rate

37 Details of any existing review of the same topic by the same authors

Give details of earlier versions of the systematic review if an update of an existing review is being registered, including full bibliographic reference if possible.

38 Current review status

Review status should be updated when the review is completed and when it is published.

Completed but not published

01/02/2017

39 Any additional information

Provide any further information the review team consider relevant to the registration of the review.

40 Details of final report/publication(s)

This field should be left empty until details of the completed review are available.
Give the full citation for the final report or publication of the systematic review.
Give the URL where available.
