# Supplementary material for: Decline in infection-related morbidities following drug-mediated reductions in the intensity of Schistosoma infection: A systematic review and meta-analysis
Source: PLoS Negl Trop Dis. 2017 Feb 17;11(2):e0005372. doi: 10.1371/journal.pntd.0005372 (PMC5333910; doi:10.1371/journal.pntd.0005372)
Supplement: S1 Text — Tables A-K indicating the main characteristics of included studies evaluating the impact of chemotherapy on different Schistosoma infection-related morbidities. (PDF) [file pntd.0005372.s003.pdf]

S1 Table A. Main characteristics of included studies evaluating the impact of chemotherapy on prevalence of hepatomegaly

Publication	Country	Species*	Time follow-up (months)	Age	Participants (Baseline/ follow-up)	Treatment**	Method for assessing morbidity***	Results****
Butterworth AE *et al.* 1991	Kenya	S.m	12/24/36	7-15	OX 733/586/499/ 431<br>PZQ 728/594/477/ 499	OX 30<br>PZQ 40	CE: liver surpassed the costal margin	OX ⬇ from 17,2% to 3,1%, 4% and 4,9%.<br>PZQ ⬇from 15,4% to 2,2%, 5% and 2,1%
DeStigter KV *et al.* 1989	Kenya	S.m	84	All ages	412/247	HY 1,5	CE: liver surpassed the costal margin	⬇ from 18% to 6%.
Doehring-Schwerdtfeger E *et al.* 1992	Sudan	S.m	7/23	School Age	322/322	PZQ 20 and 40	US: Left lobe, height-adjusted	⬇ from 10.9% to 6.8% and 7.1%
Gryseels B *et al.* 1994	Burundi	S.m	12/24/36	All ages (Cross-section community)	Community 1 706/679/649/ 634<br>Community 2 732/714/665/ 594	PZQ 40	CE: liver surpassed the costal margin	Community 1<br>⬆ from 27,1% to 34,2%, 37,3% and 34,2%.<br>Community 2<br>⬆ from 25,3% to 30%, 33,4% and 33,5%
Homeida MA *et al.* 1991	Sudan	S.m	36	Selected (Symmer´s fibrosis)	87/68	PZQ 40	CE: liver surpassed the costal margin	⬇ from 36,7% to 11,7%
Kabatereine NB *et al.* 2007	Uganda	S.m	12/24	6-14	180/180	PZQ 40 + ALB 400	US: PSL, height-adjusted	⬆ from 54,4% to 60% and ⬇ to 52,8%
Ruiz-Guevara R *et al.* 2007	Venezuela	S.m	60	8-54	78/69	PZQ 40	CE: liver surpassed the costal margin	⬇ from 55,1% to 49,3%
Sleigh AC *et al.* 1986	Brazil	S.m	12/24/96	All ages (Cross-section community)	186/145/ 122/136	OX 20 or 15	CE: liver surpassed the costal margin	⬇from 90% to 68%, 56% and 31%
Sukwa TY*et al.* 1987<br>Sukwa TY*et al.* 1988	Zambia	S.m	1987:16<br>1988:36	3-60	1987: 470/470<br>1988: 244/244	PZQ 40	CE: MSL and MCL (liver surpassed the costal margin)	1987: MSL: ⬇from 77,4% to 64,8%<br>MCL: ⬇ from 69,4% to 56,8%<br>1988: MSL: ⬇from 81,5% to 26,6%<br>MCL: ⬇ from 68% to 27,5%

Sukwa TY 1993	Zambia	S.m	12	7-19	Group A (2x treatment): 176/176<br>Group B (1x treatment): 167/167	PZQ 40	CE: MSL and MCL (liver surpassed the costal margin)	A: MSL:⬇from 34,7% to 9%<br>MCL: ⬇ from 9,7% to 1,1%<br>B: MSL ⬇from 37,7% to 10,8%<br>MCL: ⬇from 4,8% to 0,6%
Carlton EJ *et al.* 2010	China	S.j	24/60	4-60	462/440/317	PZQ	US: left and right hepatic lobe, height-adjusted	Left lobe: = from 16% to 15% and ⬆ to 21%<br>Right lobe:⬇ from 24% to 9% and 13%.
Hadidjaja P *et al.* 1985	Indonesia	S.j	8	All ages	159/159	PZQ 30	CE: liver surpassed the costal margin	⬇from 67,9% to 50,9%
Li YS *et al.* 2000<br>Li YS *et al.* 2002	China	S.j	2000:24<br>2002:60	9-65	2000: 193/193<br>2002: 120/120	PZQ 40	US: left hepatic lobe<br>(Cairo protocol)	2000: ⬇from 26,4% to 19,2%<br>2002: ⬇from 24,2% to 11,7%
Wiest PM *et al.* 1994	China	S.j	12/24	All ages (Cross-section community)	825/592/592	PZQ 50	US: MCL>=2cm MSL>=3cm	MCL: ⬇ from 34% to 25% and 18%.<br>MSL: ⬇from 89%, to 70% and 66%
Zhao G *et al.* 1995	China	S.j	12	3-60	592/592	PZQ 60	CE: MSL and MCL (liver surpassed the costal margin)	MSL: ⬇from 62,8% to 44,6%<br>MCL: ⬆ from 15% to 22,8%
Stephenson LS *et al.* 1989	Kenya	S.h	8	6-17	PZQ:105/105<br>MET:103/103<br>PLB:104/104	PZQ 40<br>MET 10<br>PLB	CE: >=1cm below right costal margin	PZQ: ⬇ from 11,4% to 10,5%<br>MET:⬆ from 8,7% to 12,6%<br>PLB: ⬆ from 7,7% to 15,4%
Stephenson LS *et al.* 1985	Kenya	S.h	6	6-15	MET:202/202<br>PLB:198/198	MET 20<br>PLB	CE: >=1cm below right costal margin	MET-⬇ from 14% to 8%,<br>PLB-⬆from 19% to 28%.
Koukounari A *et al.* 2010	Mali	S.m/S.h	12	7-14	853/853	PZQ	US: left hepatic lobe<br>CE: MCL >2cm	⬇ from 1,4% to 0,2%<br>MCL:⬇ from 4,45% to 0,7%

* Sm: *Schistosoma mansoni,* Sj: *Schistosoma japonicum*, Sh: *Schistosoma haematobium*

** PZQ: Praziquantel, OX: Oxaminiquine, MET: Metrifonate, HY: Hycanthone, PLB: Placebo, ALB: Albendazole. Following number indicates dosage/kg of one therapy course.

***CE: Clinical examination; US: ultrasonography; MCL: midclavicular line; MSL: midsternal line; PSL: parasternal line

****Decreased:⬇, Increased: ⬆, Unchanged: =

S1 Table B. Main characteristics of included studies evaluating the impact of chemotherapy on prevalence of splenomegaly

Publication	Country	Species*	Time follow-up (months)	Age	Participants (Baseline/ follow-up)	Treatment **	Method for assessing morbidity***	Results****
Cook JA *et al.* 1977	St. Lucia	S.m	6	School age	HY: 16<br>PLB: 16	HY 2,5	CE: HG > 0	HY: = from 12,5% to 12,5%<br>PLB: = from 31,25% to 31,25%
Cota GF *et al.* 2006	Brazil	S.m	48	15-43<br>Selected (all hepatosplenomegaly)	42/42	OX 15 or 20	US: cranio-caudal extension, height adjusted	⬇ from 40% to 21,4%
DeStigter KV *et al.* 1989	Kenya	S.m	84	All ages	412/247	HY 1,5	CE: HG > 1	⬇ from 3% to 0,8%.
Doehring-Schwerdtfeger E *et al*. 1992	Sudan	S.m	7/23	School Age	322/322	PZQ 20 and 40	US: cranio-caudal extension, height adjusted	⬆ from 36,1% to 39,8% and 40,1%
Gryseels B *et al*. 1994	Burundi	S.m	12/24/36	All ages<br>(Cross-section community)	Community 1<br>706/679/649/634<br>Community 2<br>732/714/665/594	PZQ 40	CE: HG > 1	Community 1<br>⬆ from 20,8% to 22,1% and to ⬇17,6% and 13,6%.<br>Community 2<br>⬆ from 33,7% to 36,7%, 37,3% and 38,9%
Homeida MA *et al.* 1991	Sudan	S.m	36	Selected (all with Symmer´s fibrosis)	87/68	PZQ 40	CE	⬇ from 47,1% to 30,9%
Ruiz-Guevara R *et al*. 2007	Venezuela	S.m	60	8-54	78/70	PZQ 40	CE: spleen surpassed the costal margin	⬇ From 3,8% to 0%
Sleigh AC *et al.* 1986	Brazil	S.m	12/24/96	All ages<br>(Cross-section community)	186/145/122/136	OX 20 or 15	CE: spleen surpassed the costal margin	⬇ from 18% to 12%, 7% and 3%
Sukwa TY *et al.* 1987<br>Sukwa TY*et al.* 1988	Zambia	S.m	16<br>36	3-60	1987: 470/470<br>1988: 244/244	PZQ 40	CE: HG > 0	1987:⬇ from 80,2% to 69,8%<br>1988:⬇ from 77,9% to 51,2%
Sukwa TY 1993	Zambia	S.m	12	7-19	Group A (2x treatment): 176/176<br>Group B (1x treatment): 167/167	PZQ 40	CE: HG > 0	A:⬇ from 16,5% to 5,1%<br>B:⬇ from 15,6% to 6%

Stephenson LS *et al.* 1989	Kenya	S.h	8	6-17	PZQ:105/105<br>MET:103/103<br>PLB:104/104	PZQ 40<br>MET 10<br>PLB	CE: below costal margin<br>Hackett grade > 0	PZQ:⬆from 46,6% to 63,8%<br>MET:⬆from 54,4% to 61,2%<br>PLB:⬆from 52% to 88%
Stephenson LS *et al.* 1985	Kenya	S.h	6	6-15	MET:202/202<br>PLB:198/198	MET 20<br>PLB	CE: below costal margin<br>HG > 0	MET-⬇from 62% to 47%,<br>PLB-⬆from 58% to 72%.
Carlton EJ *et al.* 2010	China	S.j	24/60	4-60	460/439/316	PZQ	US: from the hilum to the opposite section, height adjusted	⬇ from 4% 3% and 2%.
Hadidjaja P *et al.* 1985	Indonesia	S.j	8	All ages	159/159	PZQ 30	CE: HG > 1	⬆from 45,9% to 47,2%
Li YS *et al.* 2000<br>Li YS *et al.* 2002	China	S.j	2000: 24<br>2002: 60	9-65	2000: 193/193<br>2002: 120/120	PZQ 40	US: Cairo protocol.	2000:⬇ from 4,1% to 1,6%<br>2002:⬇ from 5,8% to 4,2%
Wiest PM *et al.* 1994	China	S.j	12/24	All ages (Cross-section community)	825/592/592	PZQ 50	US: HG > 1	⬇ from 22% to 5% and 2%.
Zhao G *et al.* 1995	China	S.j	12	3-60	592/592	PZQ 60	CE: HG > 0	⬇ from 22,12% to 20,1%
Koukounari A *et al.* 2010	Mali	S.m/S.h	12	7-14	853/853	PZQ	CE: MCL > 2cm	⬇from 16,1% to 4,8%

* Sm: *Schistosoma mansoni,* Sj: *Schistosoma japonicum*, Sh: *Schistosoma haematobium*

** PZQ: Praziquantel, OX: Oxaminiquine, MET: Metrifonate, HY: Hycanthone; PLB: Placebo, ALB: Albendazole. Following number indicates dosage/kg of one therapy course.

***CE: Clinical examination; US: ultrasonography; HG: Hackett grade

****Decreased:⬇ , Increased:⬆ , Unchanged: =

S1 Table C. Main characteristics of included studies evaluating the impact of chemotherapy on prevalence of periportal fibrosis

Publication	Country	Species*	Time follow-up (months)	Age	Participants (Baseline/ follow-up)	Treatment**	Method for assessing morbidity***	Results****
Berhe N *et al.* 2008	Ethiopia	S.m	26	22-26 (Sellected; all with Periportal fibrosis)	199/199	PZQ 40	WHO-Niamey classification	⬇ from 100% to 65,3%
Cota GF *et al.* 2006	Brazil	S.m	48	15-43 (all hepatosplenic)	42/42	OX 15 or 20	Two degrees of severity	⬇ from 45,2% to 30,9%
Doehring-Schwerdtfeger E *et al.* 1992	Sudan	S.m	7/23	School Age	322/322	PZQ 20 and 40	Three degrees of severity	⬇ from 36,6% to 34,8% and 21,7%
Frenzel K *et al.* 1999	Uganda	S.m	Group A: 12/31 Group B: 31	All ages	A: 460/460 B: 192/192	PZQ 40	Three degrees of severity. Managil classification.	A:⬇ from 46% to 32% and 35% B:⬇ from 51% to 28%
Homeida MA *et al.* 1991	Sudan	S.m	12/24/36	Selected (all with Symmer´s fibrosis)	48/48/48	PZQ 40	Three degrees of severity	⬇ from 100% to 97%, 91% and 88%
Martins-Leite P *et al.* 2008	Brazil	S.m	12	14-85	91/91	PZQ 50	Three degrees of severity.	⬇ from 49,4% to 36,3%
Rahound S *et al.* 2010	Sudan	S.m	39	All ages (all with Periportal fibrosis)	177/177	PZQ 40	Three degrees of severity. Cairo Work group classification	⬇ from 100% to 72,3%
Ruiz-Guevara R *et al.* 2007	Venezuela	S.m	60	8-54	78/78	PZQ 40	N-BH classification (degrees of fibrosis)	⬇ From 29,5% to 5,1%
Carlton EJ *et al.* 2010	China	S.j	24/60	4-60	578/444/321	PZQ	Three degrees of severity. Cairo Work group classification	⬆ from 3,63% to 4,3% and ⬇ to 0,93%.
Li YS *et al.* 2000 Li YS *et al.* 2002	China	S.j	24 60	9-65	2000: 193/193 2002: 120/120	PZQ 40	Three degrees of severity. Cairo Work group classification	2000: ⬇ from 48,7% to 39,4% 2002:⬆ from 20,8% to 25,8%
Wiest PM *et al.* 1994	China	S.j	12/24	All ages (Community base)	631/542/507	PZQ 50	Three degrees of severity	⬇ from 46% to 28% and 38%.

* *Schistosoma mansoni,* Sj: *Schistosoma japonicum*

** PZQ: Praziquantel, OX: Oxaminiquine. Following number indicates dosage/kg of one therapy course.

***N-BH: Niamey-Belo Horizonte

****Decreased:⬇, Increased:⬆

S1 Table D. Main characteristics of included studies evaluating the impact of chemotherapy on prevalence of dilated portal vein

Publication	Country	Species*	Time follow-up (months)	Age	Participants (Baseline/ follow-up)	Treatment**	Method for assessing morbidity***	Results****
Carlton EJ *et al.* 2010	China	S.j	24	4-60	462/440	PZQ	Measurements and classifications were according to WHO Guidelines.	⬆ from 10% to 13%
Li YS *et al.* 2000<br>Li YS *et al.* 2002	China	S.j	2000: 24<br>2002: 60	9-65	2000:193/193<br>2002:120/120	PZQ 40	Measurements and classifications were according to the CAIRO protocol.	2000: ⬇ from 16,06% to 11,4%<br>2002: ⬇ from 19,1% to 10,8%
Vennervald BJ *et al.* 2005	Kenya	S.m	12/24/36	7-18 all with the left lobe liver enlargement	67/67/67/67	PZQ 40	Measurements and classifications were according to WHO Guidelines.	⬇ from 16,4% to 13,4%, 11,9% and 2,3%
Kabatereine NB *et al.* 2007	Uganda	S.m	12/24	6-14	180/180/180	PZQ 40 + ALB 400	Measurements and classifications were according to the WHO protocol (N-BH)	⬇ from 17,7% to 2,2% and 3,3%

* Sm: *Schistosoma mansoni,* Sj: *Schistosoma japonicum*

** PZQ: Praziquantel, ALB: Albendazole. Following number indicates dosage/kg of one therapy course.

**** WHO Guidelines: Richter J, Hatz C, Campagne G, Bergquist NR, Jenkins JM (2000). Ultrasound in Schistosomiasis: A practical guide to the standard use of ultrasonography for the assessment of schistosomiasis-related morbidity. Geneva: WHO; N-BH: Niamey-Belo Horizonte.

***Decreased:⬇ , Increased: ⬆

S1 Table E. Main characteristics of included studies evaluating the impact of chemotherapy on prevalence of diarrhea

Publication	Country	Species*	Time follow-up (months)	Age	Participants (Baseline/ follow-up)	Treatment**	Method for assessing morbidity	Results***
Betson M *et al.* 2012	Uganda	S.m	6/12	1-5	377/372/369	PZQ 40 + ALB	Questionnaire	⬇ from 36,6% to 28,2% and ⬆ to 52,6%
Cook JA *et al.* 1977	St. Lucia	S.m	6	School Age	HY: 16/16<br>PLB: 16/16	HY 2,5	Questionnaire-previous 6 months	HY:⬇ from 56,2% to 12,5<br>PLB:⬇ from 25% to 12,5%
Gryseels B *et al.* 1994	Burundi	S.m	6/24/36	All ages (2 Cross-section community)	A: 706/693/ 649/634<br>B: 732/751/ 665/594	PZQ 40	Questionnaire-previous 3 months	A:⬇ from 26,2% to 21,1%, 13,6% and 10,2%<br>B:⬇from 19,1% to 13,4%, ⬆ 15% and ⬇ to 10,1%
Kongs A *et al.* 1996	Senegal	S.m	12	All ages	279/279	PZQ 30	Questionnaire-previous 15 days	⬇ from 54,8% to 29,03%
Peixinho E *et al.* 1986	Brazil	S.m	2	9-18	51/51	OX + MEB	Questionnaire	⬇ from 74,5% to 13,72%
Sukwa TY *et al.* 1987	Zambia	S.m	16	All ages	523/523	PZQ 40	Questionnaire-previous 15 days	⬇ from 31,35% to 14,53%
Zhao G *et al.* 1995	China	S.j	12	All ages	592/592	PZQ 60	Questionnaire-previous 15 days	⬇ from 15% to 25%

* Sm: *Schistosoma mansoni,* Sj: *Schistosoma japonicum*

** PZQ: Praziquantel, MEB: Mebendazole, OX: Oxaminiquine, HY: Hycanthone, PLB: Placebo. Following number indicates dosage/kg of one therapy course.

***Decreased: ⬇, Increased: ⬆

S1 Table F Main characteristics of included studies evaluating the impact of chemotherapy on prevalence of blood in stool

Publication	Country	Species*	Time follow-up (months)	Age	Participants (Baseline/ follow-up)	Treatment**	Method for assessing morbidity	Results***
Betson M *et al.* 2012	Uganda	S.m	6/12	1-5	377/372/369	PZQ 40 + ALB	Questionnaire	⬇ from 11,9% to 6,2% and ⬆ to 11,1%
Boisier P *et al.* 1998	Madagascar	S.m	24/36	All ages	289/289/289	PZQ 40	Questionnaire	⬇ from 24,9% to 4,6% and 8,4%
Kongs A *et al.* 1996	Senegal	S.m	12	All ages	279/279	PZQ 30	Questionnaire-previous 15 days	⬇ from 44,1% to 11,1%
Peixinho E *et al.* 1986	Brazil	S.m	2	9-18	51/51	OX + MEB	Questionnaire	⬇ from 74,5% to 13,72%
Sukwa TY *et al.* 1987	Zambia	S.m	16	All ages	523/523	PZQ 40	Questionnaire-previous 15 days	⬇ from 12,42% to 1,91%
Sukwa TY *et al.* 1993	Zambia	S.m	6	7-19	A: 190/185<br>B: 187/180	PZQ 40	Questionnaire-previous 15 days	A:⬇ from 52,6% to 23,24%<br>B:⬇ from 47,5% to 24,4%
Zhao G *et al.* 1995	China	S.j	12	All ages	592/592	PZQ 60	Questionnaire-previous 15 days	⬇ from 26,68% to 19,93%

* Sm: *Schistosoma mansoni,* Sj: *Schistosoma japonicum*

** PZQ: Praziquantel, MEB: Mebendazole, OX: Oxaminiquine, ALB: Albendazole. Following number indicates dosage/kg of one therapy course.

***Decreased: ⬇, Increased: ⬆

S1 Table G. Main characteristics of included studies evaluating the impact of chemotherapy on prevalence of blood in urine

Publication	Country	Species*	Time follow-up (months)	Age	Participants (Baseline/ follow-up)	Treatment**	Method for assessing morbidity***	Results****
Campagne G *et al.* 2001	Niger	S.h	5/34	7-15	114/105/87	PZQ 40 + ALB	Nephur-7-test	⬇from 87,7% to 24,7% and 25,2%
Delegue P *et al.* 1998	Senegal	S.h	4	All ages	203/182	PZQ 40	Hemastix Bayer	⬇ from 34,9% to 9,8%
Kahama AI *et al.* 1999	Kenya	S.h	6/18	6-15	117/117/117	PZQ 40	Hemastix Bayer	⬇ from 92,3% to 24,7% and ⬆ to 63,8%
Kiliku FM *et al.* 1991	Kenya	S.h	3	All ages	426/426	PZQ 40	Uro-Labstix III	⬇ from 40,6% to 15,2%
King C *et al.* 1990	Kenya	S.h	12	4-21	MET: 896/705<br>PZQ: 877/695	MET 10 (3x)<br>PZQ 40	Fisher Scientific	MET:⬇ from 74% to 17%<br>PZQ:⬇ from 76% to 17%
Kitange HM *et al.* 1993	Tanzania	S.h	12	7-19	253/253	PZQ 40 + ALB	BM TEST 5L	⬇ from 38,3% to 27,6%
Koukounari A *et al.* 2007	Burkina Faso	S.h	12	6-14	1.124/1.124	PZQ 40 + ALB	Hemastix Bayer	⬇ from 49,4% to 10,5%
Mekonnen A *et al.* 2013	Ethiopia	S.h	2	All ages Selected (all with hematuria)	152/152	PZQ 40	URS-11	⬇ from 100% to 40,7%
Mott KE *et al.* 1985	Ghana	S.h	6	All ages	230/230	PZQ 40	Neostix-3	⬇ from 76,5% to 26,9%
Rasendramino MH *et al.* 1998	Madagascar	S.h	12	All ages	435/435	PZQ 40	Néphur 7 test	⬇ from 72,4% to 31,4%
Sarda RK *et al.* 1987	Tanzania	S.h	6	School Age	PZQ: 67/67<br>PLB: 30/30	PZQ 40 + ALB	Combur Test	PZQ:⬇ from 85% to 2,9%<br>PLB:⬆from 86,6% to 96,6%
Sissoko MS *et al.* 2009	Mali	S.h	1	6-15	387/397	PZQ 40 + ALB 400	Hemastix Bayer	⬇ from 87,5% to 49,8%
Stephenson LS *et al.* 1984	Kenya	S.h	6	6-16	MET:244/244<br>PLB:202/202	MET 7,5 (3x)	Ames N-Multistix	MET:⬇ from 91,8% to 29%<br>PLB: = 90%

Stete K *et al.* 2012	Côte d'Ivoire	S.h	2	School Age	90/90	PZQ 40	Combur-7-TestR	⬇ from 87,7% to 16,6%
Tohon ZB *et al.* 2008	Niger	S.h	12	7-11	1.412/1.412	PZQ 40 + ALB	Hemastix Bayer	⬇ from 53,4% to 6%
Wagatsuma Y *et al.* 1999	Ghana	S.h	6/18	All ages	1.202/660/595	PZQ 40 + ALB	-	⬇ from 77,5% to 14,4% and ⬆ to 29,9%

* Sh: *Schistosoma haematobium*

** PZQ: Praziquantel, MET: Metrifonate, ALB: Albendazole, PLB: Placebo. Following number indicates dosage/kg of one therapy course.

*** All: semi-quantitatively using reagent strips.

****Decreased: ⬇ , Increased: ⬆ , Unchanged: =

S1 Table H. Main characteristics of included studies evaluating the impact of chemotherapy on prevalence of protein in urine

Publication	Country	Species*	Time follow-up (months)	Age	Participants (Baseline/ follow-up)	Treatment**	Method for assessing morbidity***	Results****
Kiliku FM *et al*. 1991	Kenya	S.h	3	All ages	426/426	PZQ 40	Uro-Labstix III	⬇ from 62% to 48,3%
King C *et al.* 1990	Kenya	S.h	12	4-21	MET: 896/705<br>PZQ: 877/695	MET 10 (3x)<br>PZQ 40	Fisher Scientific	MET: ⬇ from 72% to 29%<br>PZQ: ⬇ from 75% to 27%
Kitange HM *et al.* 1993	Tanzania	S.h	12	7-19	253/253	PZQ 40 + ALB	BM TEST 5L	⬆ from 12,2% to 15,8%
Koukounari A *et al.* 2007	Burkina Faso	S.h	12	6-14	1.124/1.124	PZQ 40 + ALB	Hemastix Bayer	⬇ from 49,4% to 10,5%
Mekonnen A *et al.* 2013	Ethiopia	S.h	2	All ages, Selected (all with hematuria)	152/152	PZQ 40	URS-11	⬇ from 94,07% to 48,7%
Mott KE *et al.* 1985	Ghana	S.h	6	All ages	230/230	PZQ 40	Neostix-3	⬇ from 90,8% to 42,6%
Rasendramino MH *et al.* 1998	Madagascar	S.h	12	All ages	435/435	PZQ 40	Néphur 7 test	⬇ from 62,3% to 20,2%
Sarda RK *et al.* 1987	Tanzania	S.h	6	School Age	PZQ: 67/67<br>PLB: 30/30	PZQ 40 + ALB	Combur Test	PZQ: ⬇ from 79,1% to 5,8%<br>PLB: ⬆ from 70% to 90%
Stephenson LS *et al.* 1984	Kenya	S.h	6	6-16	MET:244/244<br>PLB:202/202	MET 7,5 (3x)	Ames N-Multistix	MET: ⬇ from 76% to 14%<br>PLB: ⬇ from 63% to 54%
Stete K *et al.* 2012	Côte d'Ivoire	S.h	2	School Age	90/90	PZQ 40	Combur-7-TestR	⬇ from 70% to 11,6%
Wagatsuma Y *et al.* 1999	Ghana	S.h	6/18	All ages	1.202/660/595	PZQ 40 + ALB	-	⬇ from 74,5% to 1,3% and ⬆ to 8,6%

* Sh: *Schistosoma haematobium*

** PZQ: Praziquantel, MET: Metrifonate, ALB: Albendazole, PLB: Placebo. Following number indicates dosage/kg of one therapy course.

*** All: semi-quantitatively using reagent strips.

****Decreased: ⬇, Increased: ⬆

S1 Table I. Main characteristics of included studies evaluating the impact of chemotherapy on prevalence of ultrasound abnormalities in the urinary bladder

Publication	Country	Species*	Time follow-up (months)	Age	Participants (Baseline/ follow-up)	Treatment**	Method for assessing morbidity***	Results****
Campagne G *et al.* 2001	Niger	Sh	2/34	7-15	114/112/87	PZQ 40	BL: Shape and/or TW and/or BW Irreg. and/or Mass and/or PP	⬇ from 89,5% to 48,2% and 72,4%
Delegue P *et al.* 1998	Senegal	Sh	4	All ages	203/182	PZQ 40	BL: TW	⬇ from 22,6% to 8,8%
Devidas A *et al.* 1989	Niger	Sh	10	All ages	149/149	PZQ 40	BL: TW and/or BW Irreg. and/or Hypertrophy.	⬇ from 79,8 to 32,2%
Doehring E *et al.* 1986	Congo	Sh	12	All ages Selected- Pathological ultrasonographical findings of the urinary tract	103/103	PZQ 40	BL: TW and/or PP and/or Calcification	⬇ from 100% to 7,8%
Kahama AI *et al.* 1999	Kenya	Sh	4/12/18	6-15	117/117/117	PZQ 40	BL: TW and/or Mass and/or PP and/or Dilatation of the Ureter	⬇ from 58,12% to 17,9% and ⬆ to 51,2%
King CH *et al.* 1988	Kenya	Sh	12	4-21	363/363	PZQ 40 or MET 30	BL: TW	⬇ from 19% to 6,8%
King CH *et al.* 2002	Kenya	Sh	9	4-23	1: 99/99<br>2: 101/101	1: PZQ 20<br>2: PZQ 40	BL: TW and/or BW Irreg.	1: ⬇ from 21,2% to 6,1%<br>2: ⬇ from 18,8% to 3,9%
Ramarakoto CE *et al.* 2008	Madagascar	Sh	6	Selected (no children) 17-48	130/130	PZQ 40	BL: Shape and/or TW and/or BW Irreg. and/or mass and/or PP	⬇ from 68,5% to 21,5%
Rasendramino MH *et al.* 1998	Madagascar	Sh	12	All ages	472/472	PZQ 40	BL: Shape and/or TW and/or BW	⬇ from 50% to 16,1%

							Irreg. and/or Mass	
Reimert CM *et al.* 2000	Tanzania	Sh	4/12/18	7-17	514/384/423/ 422	PZQ 40	BL: TW and/or BW Irreg. and/or Mass and/or PP	⬇ from 62,2% to 15,6% and ⬆ to 19,6% and 31,7%
Tohon ZB *et al.* 2008	Niger	Sh	12	7-11	1.409/1.409	PZQ 40 + ALB 400	BL: Niamey protocol	⬇from 41,6% to 14,7%
Traore M *et al.* 1998	Mali	Sh	12	All ages	648/648	PZQ 40	BL: BW Irreg.	⬇ from 25,5% to 14,9%
Hatz C *et al.* 1990	Tanzania	Sh	6	7-20, Selected- Pathological ultrasonographical findings of the urinary tract	1: 72/72<br>2: 52/52	1: PZQ 40<br>2: PZQ 20	BL: TW and/or BW Irreg. and/or Mass and/or PP.	1: ⬇ from 86,1% to 5,5%<br>2: ⬇ from 80,7% to 1,9%

* Sh: *Schistosoma haematobium*

** PZQ: Praziquantel, MET: Metrifonate, ALB: Albendazole. Following number indicates dosage/kg of one therapy course.

***BL: bladder lesion, TW: thickened wall, Irreg.: Bladder Wall Irregularity, PP: pseudo-polyp.

****Decreased:⬇, Increased:⬆

S1 Table J. Main characteristics of included studies evaluating the impact of chemotherapy on prevalence of ultrasound abnormalities in the upper urinary tract

Publication	Country	Species*	Time follow-up (months)	Age	Participants (Baseline/ follow-up)	Treatment**	Method for assessing morbidity	Results***
Campagne G *et al.* 2001	Niger	Sh	34	7-15	114/87	PZQ 40	Hydronephosis	⬇ from 27,19% to 4,6%
Delegue P *et al.* 1998	Senegal	Sh	4	All ages	203/182	PZQ 40	Hydronephosis	⬇ from 2,5% to 0%
Devidas A *et al.* 1989	Niger	Sh	10	All ages	149/149	PZQ 40	Hydronephosis	⬇ from 48,9% to 17,4%
King CH *et al.* 1988	Kenya	Sh	12	4-21	363/363	PZQ 40 or MET 30	Hydronephosis	⬆ from 14,05% to 16%
King CH *et al.* 2002	Kenya	Sh	9	4-23	1: 99/99<br>2: 101/101	1: PZQ 20<br>2: PZQ 40	Hydronephosis	⬇ 1: from 35,4% to 21,2%<br>⬇ 2: from 34,6% to 19,8%
Rasendramino MH *et al.* 1998	Madagascar	Sh	12	All ages	472/472	PZQ 40	Hydronephosis	⬇ from 8,7% to 2,3%
Tohon ZB *et al.* 2008	Niger	Sh	12	7-11	1.409/1.409	PZQ 40 + ALB 400	Hydronephosis	⬇ from 4% to 0,3%
Traore M *et al.* 1998	Mali	Sh	12	All ages	648/648	PZQ 40	Ureteral dilatation	⬇ from 17% to 5%

* Sh: *Schistosoma haematobium*

** PZQ: Praziquantel, MET: Metrifonate, ALB: Albendazole. Following number indicates dosage/kg of one therapy course.

***Decreased:⬇, Increased:⬆

S1 Table K. Main characteristics of included studies evaluating the impact of chemotherapy on blood hemoglobin

Publication	Country	Species*	Time follow-up (months)	Age	Participants	Treatment**	Method for assessing morbidity	Results*** Mean g/dL (SD)
Awad El Karim MA *et al.* 1981	Sudan	S.m	12	18-45, Selected (males/workers)	HY:22<br>PLB:19	HY 3	Venous blood	**HY** B: 14,1(0,9)<br>F: 15,2 (0,9)<br>**PLB** B: 14,8 (1,3)<br>F: 15,3 (1,1)
Kabatereine NB *et al.* 2007	Uganda	S.m	12/24	6-14	1.852	PZQ 40 + ALB 400	capillary blood<br>Finger prick method	B: 11,4 (2,15)<br>F (12): 11,7 (2,15)<br>F (24): 12 (2,15)
Ndamba J *et al.* 1993	Zimbabwe	S.m	4	20-54 Selected (males/workers)	PZQ:287<br>CON:210	PZQ 40	Venous blood	**PZQ** B: 14,1 (2,9)<br>F: 14,3 (2,8)<br>**CON** B: 14,5 (1,7)<br>F: 14,8 (2,7)
Ayoya M *et al.* 2009	Mali	S.h	3	7-12 Selected - All anemic Hb:>7 and <12 g/dL	97	PZQ 40	Venous blood	B: 10,37 (1,0)<br>F: 10,81 (0,8)
Beasley NMR *et al.* 1999	Tanzania	S.h	4	7-12 Selected - infected with both S.h and at least one species of geohelminth	PZQ:127<br>PLB:123	PZQ 40 + ALB 400	Venous blood	**PZQ** B: 11 (0,09)<br>F: 10,9 (0,08)<br>**PLB** B: 11 (0,009)<br>F: 10,7 (0,1)
Bhargava A *et al.* 2003	Tanzania	S.h	3/15	9-15 Hb:>8g/dL	PZQ: 79<br>PZQ/ALB: 135	PZQ 40<br>PZQ 40 + ALB 400	Venous blood	**PZQ**<br>B: 11,4 (1,3)<br>F (2): 11,4 (1,4)<br>F (15): 11,9 (1,3)<br>**PZQ + ALB**<br>B: 11,21 (1,5)<br>F (3): 11,56 (1,3)<br>F (15):12,09 (1,1)
Koukounari A *et al.* 2007	Burkina Faso	S.h	12	5-15	1.131	PZQ 40 + ALB 400	capillary blood<br>Finger prick method	B: 10,97 (0,08)<br>F: 11,25 (0,07)
Latham MC *et al.* 1983	Kenya	S.h	4	Mean: 31,4 Selected (males/workers)	MET:52<br>CON: 91	MET 20	capillary blood<br>Finger prick method	**MET** B: 13,2 (1,7)<br>F: 13,6 (1,5)<br>**CON** B: 12,7 (2)

								F: 13,3 (2)
Mwanakasale V *et al.* 2009	Zambia	S.h	9	9-15	153/153	PZQ	Venous blood	B: 11,7 (1,5)<br>F: 12,6 (1,8)
Sissoko MS *et al.* 2009	Mali	S.h	1	6-15	389	PZQ 40 + ALB 400	Venous blood	B: 11,1 (1,5)<br>F: 13,3 (0,9)
Stephenson LS *et al.* 1989	Kenya	S.h	8	6-17<br>Hb: >8g/dL	PZQ: 105<br>MET: 103<br>PLB: 104	PZQ 40<br>MET 10	capillary blood<br>Finger prick method	**PZQ** B:11,2 (0,11)<br>F: 11,2 (0,13)<br>**MET** B:11,5(0,13)<br>F:11,6 (0,12)<br>**PLB** B: 11,5(0,11)<br>F: 11,3 (0,13)
Stephenson LS *et al.* 1985	Kenya	S.h	6	6-15	MET: 202<br>PLB: 198	MET 7,5 (3x)	capillary blood<br>Finger prick method	**MET** B:11,2(0,10)<br>F: 12,5 (0,8)<br>**PLB** B: 11,3 (0,1)<br>F: 12,3 (0,8)
McGarvey ST *et al.* 1996	Philippines	S.j	6	4-20	PZQ: 55<br>PLB: 61	PZQ 50	Capillary or venous blood	**PZQ** B: 11,1 (1,9)<br>F: 11,2 (1,5)<br>**PLB** B: 11,4 (2,2)<br>F: 10,3 (2,2)

* Sm: *Schistosoma mansoni,* Sj: *Schistosoma japonicum*, Sh: *Schistosoma haematobuim*

** PZQ: Praziquantel, ALB: Albendazole, HY: Hycanthone, MET: Metrifonate, CON: Control. Following number indicates dosage/kg of one therapy course.

*** B: Baseline, F: Follow-up

Alphabetized list of full citations for papers included in S1 Tables A-K

1. Awad El Karim MA, Collins KJ, Sukkar MY, Omer AHS, Amin MA, Doré C. An assessment of anti-schistosomal treatment on physical work capacity. J Trop Med Hyg. 1981; 84:67-72.
2. Ayoya MA, Spiekermann-Brouwer GM, Traore AK, Stoltzfus RJ, Habicht JP, Garza C. Multiple micronutrients including iron are not more effective than iron alone for improving hemoglobin and iron status of Malian school children. J Nutr. 2009;139(10):1972–9.
3. Beasley NMR, Tomkins AM, Hall A, Kihamia CM, Lorri W, Nduma B, et al. The impact of population level deworming on the haemoglobin levels of schoolchildren in Tanga, Tanzania. Trop Med Int Heal. 1999;4(11):744–50.
4. Berhe N, Myrvang B, Gundersen SG. Reversibility of schistosomal periportal thickening/fibrosis after praziquantel therapy: a twenty-six month follow-up study in Ethiopia. Am J Trop Med Hyg. 2008;78(2):228–34.
5. Betson M, Sousa-Figueiredo JC, Kabatereine NB, Stothard JR. Use of fecal occult blood tests as epidemiologic indicators of morbidity associated with intestinal schistosomiasis during preventive chemotherapy in young children. Am J Trop Med Hyg. 2012;87(4):694–700.
6. Bhargava A, Jukes M, Lambo J, Kihamia CM, Lorri W, Nokes C, et al. Anthelmintic treatment improves the hemoglobin and serum ferritin concentrations of Tanzanian schoolchildren. Food Nutr Bull. 2003;24(4):332–42.
7. Boisier P, Ramarokoto CE, Ravaoalimalala VE, Rabarijaona L, Serieye J, Roux J, Esterre P. Reversibility of Schistosoma mansoni-associated morbidity after yearly mass praziquantel therapy: ultrasonographic assessment. Trans R Soc Trop Med Hyg. 1998;92(4):451–3.
8. Butterworth AE, Sturrock RF, Ouma JH, Mbugua GG, Fulford AJC, Kariuki HC, et al. Comparison of different chemotherapy strategies against schistosoma-mansoni in Machakos district, Kenya - effects on human infection and morbidity. Parasitology. 1991;103(3):339–55.
9. Campagne G, Garba a, Barkiré H, Vera C, Sidiki a, Chippaux JP. Continued ultrasonic follow-up of children infected with Schistosoma haematobium after treatment with praziquantel. Trop Med Int Health. 2001;6(1):24–30.
10. Carlton EJ, Hsiang M, Zhang Y, Johnson S, Hubbard A, Spear RC. The impact of Schistosoma japonicum infection and treatment on ultrasound-detectable morbidity: a five-year cohort study in Southwest China. PLoS Negl Trop Dis. 2010;4(5):e685.
11. Cook JA, Jordan P, Woodstock L, Pilgrim V. A controlled trial of hycanthone and placebo in schistosomiasis mansoni in St. Lucia. Ann Trop Med Parasitol. 1977;71(2):197-203.
12. Cota GF, Pinto-Silva RA, Antunes CMF, Lambertucci JR. Ultrasound and clinical investigation of hepatosplenic schistosomiasis: Evaluation of splenomegaly and liver fibrosis four years after mass chemotherapy with oxamniquine. Am J Trop Med Hyg. 2006;74(1):103–7.
13. Delegue P, Picquet M, DJ S, Vercruysse J, Sambou B, Ly A. Morbidity induced by Schistosoma haematobium infections, as assessed by ultrasound before and after treatment with praziquantel, in a recently expanded focus (Senegal River basin). Ann Trop Med Parasitol. 1998;92(7):775–83.
14. DeStigter KV, King CH, Keating CE, Ouma JH, Siongok TK, Mahmoud AF. Effects of targeted mass treatment on intensity of infection and morbidity in scistosomiasis mansoni: seven-year follow-up of a community in machakos, kenya. Trans Assoc Am Physicians. 1989;102:209–12.
15. Devidas A, Lamothe F, Develoux M, Mouchet F, Sellin B. Ultrasonographic assessment of the regression of bladder and renal lesions due to Schistosoma haematobium after treatment with praziquantel. Ann Soc Belg Med Trop. 1989;69(1):57–65.
16. Doehring E, Ehrich JH, Bremer HJ. Reversibility of urinary tract abnormalities due to Schistosoma haematobium infection. Kidney Int. 1986;30(4):582–5.

17. Doehring-Schwerdtfeger E, Abdel-Rahim IM, Kardorff R, Kaiser C, Franke D, Schlake J, et al. Ultrasonographical investigation of periportal fibrosis in children with Schistosoma mansoni infection: Reversibility of morbidity twenty-three months after treatment with praziquantel. Am J Trop Med Hyg. 1992;46(4):409–15.
18. Frenzel K, Grigull L, Odongo-Aginya E, Ndugwa CM, Loroni-Lakwo T, Schweigmann U, et al. Evidence for a long-term effect of a single dose of praziquantel on Schistosoma mansoni-induced hepatosplenic lesions in northern Uganda. Am J Trop Med Hyg. 1999;60(6):927–31.
19. Gryseels B, Nkulikyinka L, Engels D. Impact of repeated community-based selective chemotherapy on morbidity due to schistosomiasis mansoni. Am J Trop Med Hyg. 1994;51(5):634–41.
20. Hadidjaja P, Syamsuddin N, Ismid IS, Sudomo M, Campbell J, Putrali J. The impact of schistosomiasis mass treatment on hepato-splenomegaly in Napu Valley, Central Sulawesi, Indonesia. Southeast Asian J Trop Med Public Health. 1985;16(3):401–4.
21. Hatz C, Mayombana C, Savigny D, MacPherson CNL, Koella JC, Degrémont A, Tanner M. Ultrasound scanning for detecting morbidity due to Schistosoma haematobium and its resolution following treatment with different doses of praziquantel. Trans R Soc Trop Med Hyg. 1990;84:84-88.
22. Homeida MA, El Tom I, Nash T, Bennett JL. Association of the therapeutic activity of praziquantel with the reversal of Symmers' fibrosis induced by Schistosoma mansoni. Am J Trop Med Hyg. 1991;45(3):360–5.
23. Kabatereine NB, Brooker S, Koukounari A, Kazibwe F, Tukahebwa EM, Fleming FM, et al. Impact of a national helminth control programme on infection and morbidity in Ugandan schoolchildren. Bull World Health Organ. 2007;85(2):91–9.
24. Kahama AI, Odek AE, Kihara RW, Vennervald BJ, Kombe Y, Nkulila T, et al. Urine circulating soluble egg antigen in relation to egg counts, hematuria, and urinary tract pathology before and after treatment in children infected with Schistosoma haematobium in Kenya. Am J Trop Med Hyg. 1999;61(2):215–9.
25. Kiliku FM, Kimura E, Muhoho N, Migwi DK, Katsumata T. The usefulness of urinalysis reagent strips in selecting Schistosoma haematobium egg positives before and after treatment with praziquantel. J Trop Med Hyg. 1991;94(6):401–6.
26. King CH, Lombardi G, Lombardi C, Greenblatt R, Hodder S, Kinyanjui H, et al. Chemotherapy-based control of schistosomiasis haematobia. II. Metrifonate vs. praziquantel in control of infection-associated morbidity. Am J Trop Med Hyg. 1990;42(6):587–95.
27. King CH, Lombardi G, Lombardi C, Greenblatt R, Hodder S, Kinyanjui H, et al. Chemotherapy-based control of schistosomiasis haematobia. I. Metrifonate versus praziquantel in control of intensity and prevalence of infection. Am J Trop Med Hyg. 1988;39(3):295–305.
28. King CH, Muchiri EM, Mungai P, Ouma JH, Kadzo H, Magak P, et al. Randomized comparison of low-dose versus standard-dose praziquantel therapy in treatment of urinary tract morbidity due to Schistosoma haema tobium infection. Am J Trop Med Hyg. 2002;66(6):725–30.
29. Kitange HM, Swai ABM, McLarty DG, Alberti KGMM. Schistosomiasis prevalence after administration of praziquantel to school children in Melela village, Morogoro region, Tanzania. East Afr Med J. 1993;70(12):782–6.
30. Kongs A, Verle P, Dieng A, Talla I, Rouquet P. Clinical investigation of a population recently infected with Schistosoma mansoni (Richard-Toll, Senegal). Trop Med Int Heal. 1996;1(2):191–8.
31. Koukounari A, Donnelly CA, Sacko M, Keita AD, Landouré A, Dembelé R, et al. The impact of single versus mixed schistosome species infections on liver, spleen and bladder morbidity within Malian children pre- and post-praziquantel treatment. BMC Infect Dis. 2010;10:227.
32. Koukounari A, Gabrielli AF, Toure S, Bosque-Oliva E, Zhang Y, Sellin B, et al. Schistosoma haematobium infection and morbidity before and after large-scale administration of praziquantel in Burkina Faso. J Infect Dis. 2007;196(5):659–69.
33. Latham MC, Stephenson LS, Hall A, Wolgemuth JC, Elliot TC, Crompton DET. Parasitic infections, anaemia and nutritional status: a study of their interrelationships and the effect of prophylaxis and treatment on workers in Kwale District, Kenya. Trans R Soc Trop Med Hyg. 1983;77(1):411-48.

34. Li YS, Sleigh a C, Li Y, Tanner M, Dessein a, Williams GM, et al. Five-year impact of repeated praziquantel treatment on subclinical morbidity due to Schistosoma japonicum in China. Trans R Soc Trop Med Hyg. 2002;96(4):438–43.
35. Li YS, Sleigh AC, Ross AG, Li Y, Williams GM, Tanner M, et al. Two-year impact of praziquantel treatment for Schistosoma japonicum infection in China: re-infection, subclinical disease and fibrosis marker measurements. Trans R Soc Trop Med Hyg. 2000;94(2):191–7.
36. Martins-Leite P, Gazzinelli G, Alves-Oliveira LF, Gazzinelli A, Malaquias LCC, Correa-Oliveira R, et al. Effect of chemotherapy with praziquantel on the production of cytokines and morbidity associated with schistosomiasis mansoni. Antimicrob Agents Chemother. 2008;52(8):2780–6.
37. McGarvey ST, Aligui G, Graham KK, Peters P, Olds GR, Olveda R. Schistosomiasis japonica and childhood nutritional status in northeastern Leyte, the Philippines: a randomized trial of praziquantel versus placebo. Am J Trop Med Hyg. 1996;54(5):498–502.
38. Mekonnen A, Legesse M, Belay M, Tadesse K, Torben W, Teklemariam Z, et al. Efficacy of Praziquantel against Schistosoma haematobium in Dulshatalo village, western Ethiopia. BMC Res Notes. 2013;6(1):392.
39. Mott KE, Dixon H, Osei-Tutu E, England EC, Davis A. Effect of praziquantel on hematuria and proteinuria in urinary schistosomiasis. Am Soc Trop Med Hyg. 1985;34(6):1119–26.
40. Mwanakasale V, Siziya S, Mwansa J, Koukounari A, Fenwick A. Impact of iron supplementation on schistosomiasis control in Zambian school children in a highly endemic area. Malawi Med J. 2009;21(1):12–8.
41. Ndamba J, Makaza N, Munjoma M, Gomo E, Kaondera KC. The physical fitness and work performance of agricultural workers infected with Scgistosoma mansoni in Zimbabwe. Ann Trop Med Parasitol. 1993;87(6):553-561.
42. Peixinho EL, André B, Bina JC. Sintomatologia intestinal na fase crônica da esquistossomose mansoni. Rev Soc Bras Med Trop. 1986;19(1):27–30.
43. Rahoud SA, Mergani A, Khamis AH, Saeed OK, Mohamed-Ali Q, Dessein AJ, et al. Factors controlling the effect of praziquantel on liver fibrosis in Schistosoma mansoni-infected patients. FEMS Immunol Med Microbiol. 2010;58(1):106–12.
44. Ramarakoto CE, Leutscher PDC, Van Dam G, Christensen NO. Ultrasonographical findings in the urogenital organs in women and men infected with Schistosoma haematobium in northern Madagascar. Trans R Soc Trop Med Hyg. 2008;102(8):767–73.
45. Rasendramino MH, Rajaona HR, Ramarakoto VE, Leutscher P, Cordonnier D, Esterre P. Effect du praziquantel sur les retentissements uro-néphrologiques de la bilharziose urinaire. Nephrologie. 1998;19(6):347–51.
46. Reimert CM, Mshinda HM, Hatz CF, Kombe Y, Nkulila T, Poulsen LK, et al. Quantitative assessment of eosinophiluria in Schistosoma haematobium infections: A new marker of infection and bladder morbidity. Am J Trop Med Hyg. 2000;62(1):19–28.
47. Ruiz-Guevara R, de Noya BA, Valero SK, Lecuna P, Garassini M, Noya O. Clinical and ultrasound findings before and after praziquantel treatment among Venezuelan schistosomiasis patients. Rev Soc Bras Med Trop. 2007;40(5):505–11.
48. Sarda RK, Kihamia CM, Minjas JN, Mahikwano LF. Haematuria and proteinuria in urinary schistosomiasis: response to therapy with praziquantel in Tanzanian children. Trop Med Parasitol. 1987;38(1):31–3.
49. Sissoko MS, Dabo A, Traore H, Diallo M, Traore B, Konate D, et al. Efficacy of artesunate + sulfamethoxypyrazine/pyrimethamine versus praziquantel in the treatment of Schistosoma haematobium in children. PLoS One. United States; 2009;4(10):e6732.
50. Sleigh AC, Mott KE, Hoff R, Maguire JH, da França Silva JT. Manson’s schistosomiasis in Brazil: 11-year evaluation of successful disease control with oxamniquine. Lancet. 1986;1:635–7.
51. Stephenson LS, Kurz KM, Kinoti SN, Oduori ML, Crompton DW. Relationships of Schistosoma hematobium, hookworm and malarial infections and metrifonate treatment to hemoglobin level in Kenyan school children. Am J Trop Med Hyg. 1985;34(3):519–28.

52. Stephenson LS, Kinoti SN, Latham MC, Kurz KM, Kyobe J. Single dose metrifonate or praziquantel treatment in Kenyan children. I. Effects on Schistosoma haematobium, hookworm, hemoglobin levels, splenomegaly, and hepatomegaly. Am-J-Trop-Med-Hyg. 1989;41(4):436–44.
53. Stephenson LS, Latham MC, Kinoti SN, Oduori ML. Regression of splenomegaly and hepatomegaly in children treated for Schistosoma haematobium infection. Am J Trop Med Hyg. 1985;34(1):119–23.
54. Stephenson LS, Latham MC, Kinoti SN, Oduori ML. Sensitivity and specificity of reagent strips in screening of Kenyan children for Schistosoma haematobium infection. Am J Trop Med Hyg. 1984;33(5):862–71.
55. Stete K, Krauth SJ, Coulibaly JT, Knopp S, Hattendorf J, Müller I, et al. Dynamics of Schistosoma haematobium egg output and associated infection parameters following treatment with praziquantel in school-aged children. Parasit Vectors. 2012;5(1):298.
56. Sukwa TY, Bulsara MK, Wurapa FK. Reduction in prevalence, intensity of infection and morbidity due to schistosoma mansoni infection in a community following treatment with praziquantel. J Trop Med Hyg. 1987;90(4):205-11.
57. Sukwa TY. A community-based randomized trial of praziquantel to control schistosomiasis morbidity in schoolchildren in Zambia. Ann Trop Med Parasitol. 1993;87(2):185–94.
58. Sukwa TY, Boatin BA, Wurapa FK. A three year follow-up of chemotherapy with praziquantel in a rural Zambian community endemic for schistosomiasis mansoni. Trans R Soc Trop Med Hyg. 1988;82(2):258–60.
59. Tohon ZB, Mainassara HB, Garba A, Mahamane AE, Bosqué-Oliva E, Ibrahim ML, et al. Controlling schistosomiasis: Significant decrease of anaemia prevalence one year after a single dose of praziquantel in Nigerien schoolchildren. PLoS Negl Trop Dis. 2008;2(5):e241.
60. Traore M, Traore HA, Kardorff R, Diarra A, Landoure A, Vester U, et al. The public health significance of urinary schistosomiasis as a cause of morbidity in two districts in Mali. Am J Trop Med Hyg. 1998;59(3):407–13.
61. Vennervald BJ, Booth M, Butterworth AE, Kariuki HC, Kadzo H, Ireri E, et al. Regression of hepatosplenomegaly in Kenyan school-aged children after praziquantel treatment and three years of greatly reduced exposure to Schistosoma mansoni. Trans R Soc Trop Med Hyg. 2005;99(2):150–60.
62. Wagatsuma Y, Aryeetey ME, Sack DA, Morrow RH, Hatz C, Kojima S. Resolution and resurgence of schistosoma haematobium-induced pathology after community-based chemotherapy in Ghana, as detected by ultrasound. J Infect Dis. 1999;179(6):1515–22.
63. Wiest PM, Wu G, Zhong S, McGarvey ST, Yuan J, Olveda RM, et al. Impact of annual screening and chemotherapy with praziquantel on schistosomiasis japonica on Jishan Island, People's Republic of China. Am J Trop Med Hyg. 1994;51(2):162–9.
64. Zhao G, Jiang Q, Wasley A, Zhang S, Wu Z, Liu Z, Yuan H. Changes in prevalence, intensity of infection and morbidity due to Schistosoma japonicum infection in a community following a single treatment with praziquantel. Trop Geogr Med. 1995;47(6):289-292.
