# Supplementary material for: Decline in infection-related morbidities following drug-mediated reductions in the intensity of Schistosoma infection: A systematic review and meta-analysis
Source: PLoS Negl Trop Dis. 2017 Feb 17;11(2):e0005372. doi: 10.1371/journal.pntd.0005372 (PMC5333910; doi:10.1371/journal.pntd.0005372)
Supplement: S2 Text — (PDF) [file pntd.0005372.s004.pdf]

S2 Table. Study design and quality assessment of the studies included in meta-analysis of *Schistosoma*-associated morbidity outcomes after drug therapy

Publication	Study Design*	1	2	3	4	5	6	7	8	9	10	11	12	13
Awad El Karim MA *et al.* 1981	1	Y	Y	N	CD	CD	Y	Y	Y	CD	Y	N	NA	Y
Ayoya M *et al.* 2009	1	Y	Y	N	Y	Y	Y	Y	Y	Y	Y	N	NA	Y
Beasley NMR *et al.* 1999	1	Y	Y	N	Y	Y	Y	Y	Y	N	Y	N	NA	Y
Berhe N *et al.* 2008	3	Y	Y	N	Y	CD	Y	Y	NA	CD	Y	N	NA	Y
Betson M *et al.* 2012	3	Y	Y	Y	Y	Y	Y	N	NR	CD	Y	N	NA	Y
Bhargava A *et al.* 2003	1	Y	Y	Y	N	CD	Y	Y	NR	Y	Y	N	NA	Y
Boisier P *et al.* 1998	4	Y	Y	Y	Y	Y	Y	N	NR	N	N	N	NA	Y
Butterworth AE *et al.* 1991	4	Y	Y	Y	Y	Y	Y	Y	NR	N	N	N	NA	Y
Campagne G *et al.* 2001	2	Y	Y	Y	Y	Y	Y	Y	NR	Y	Y	Y	NA	Y
Carlton EJ *et al.* 2010	2	Y	Y	Y	Y	CD	Y	Y	Y	Y	Y	N	NA	Y
Cook JA *et al.* 1977	1	Y	Y	N	CD	CD	Y	Y	NR	CD	Y	N	NA	Y
Cota GF *et al.* 2006	3	Y	Y	N	Y	Y	Y	Y	Y	N	Y	N	NA	Y
Delegue P *et al.* 1998	3	Y	Y	Y	N	CD	Y	Y	Y	Y	Y	N	NA	Y
DeStigter KV *et al.* 1989	3	Y	Y	Y	Y	Y	Y	Y	NR	N	Y	N	NA	Y
Devidas A *et al.* 1989	3	Y	Y	Y	CD	CD	Y	Y	NA	Y	Y	N	NA	Y
Doehring E *et al.* 1986	3	Y	Y	N	Y	Y	Y	Y	NA	N	Y	N	NA	Y
Doehring-Schwerdtfeger E *et al.* 1992	3	Y	Y	Y	Y	Y	Y	Y	Y	N	N	N	NA	Y
Frenzel K *et al.* 1999	2	Y	Y	Y	N	CD	Y	Y	NR	Y	Y	N	NA	Y
Gryseels B *et al.* 1994	4	Y	Y	Y	Y	Y	Y	Y	Y	Y	N	Y	NA	Y
Hadidjaja P *et al.* 1985	2	Y	Y	Y	N	Y	Y	Y	NR	CD	Y	N	NA	Y
Hatz C *et al.* 1990	1	Y	Y	N	Y	Y	Y	Y	NR	N	Y	N	NA	Y
Homeida MA *et al.* 1991	3	Y	Y	N	Y	Y	Y	Y	Y	N	Y	N	NA	Y
Kabatereine NB *et al.* 2007	2	Y	Y	Y	N	Y	Y	Y	NR	N	Y	N	NA	Y
Kahama AI *et al.* 1999	2	Y	Y	Y	Y	CD	Y	Y	NR	N	Y	N	NA	Y
Kiliku FM *et al.* 1991	2	Y	Y	Y	N	Y	Y	Y	NR	N	Y	N	NA	Y
King CH *et al.* 1988	2	Y	Y	Y	Y	Y	Y	Y	Y	N	Y	N	NA	Y
King CH *et al.* 1990	3	Y	Y	Y	Y	Y	Y	Y	Y	Y	Y	N	NA	Y
King CH *et al.* 2002	3	Y	Y	N	Y	Y	Y	Y	NR	N	Y	N	NA	Y

Kitange HM *et al.* 1993	2	Y	Y	Y	CD	CD	Y	Y	Y	CD	Y	N	NA	Y
Kongs A *et al.* 1996	2	Y	Y	Y	Y	Y	Y	Y	NR	N	Y	N	NA	Y
Koukounari A *et al.* 2007	2	Y	Y	Y	Y	Y	Y	Y	NR	N	Y	N	NA	Y
Koukounari A, 2010	3	Y	Y	Y	N	Y	N	Y	Y	N	Y	N	NA	CD
Latham MC *et al.* 1983	3	Y	Y	N	CD	CD	Y	Y	NR	CD	Y	N	NA	Y
Li YS *et al.* 2000<br>Li YS *et al.* 2002	3	Y	Y	N	CD	Y	Y	Y	Y	N	Y	N	NA	Y
Martins-Leite P *et al.* 2008	3	Y	Y	Y	CD	CD	Y	Y	Y	CD	Y	N	NA	Y
McGarvey ST *et al.* 1996	1	Y	Y	N	Y	Y	Y	Y	Y	N	Y	N	NA	Y
Mekonnen A *et al.* 2013	3	Y	Y	N	Y	Y	Y	Y	NR	CD	Y	N	NA	Y
Mott KE *et al.* 1985	3	Y	Y	Y	Y	Y	Y	Y	NR	N	Y	N	NA	Y
Mwanakasale V *et al.* 2009	1	Y	Y	Y	Y	Y	N	Y	NR	N	Y	N	NA	Y
Ndamba J *et al.* 1993	3	Y	Y	N	Y	Y	Y	Y	NR	CD	Y	N	NA	Y
Peixinho E *et al.* 1986	2	Y	Y	Y	Y	Y	Y	Y	NR	Y	Y	N	NA	Y
Rahound S *et al.* 2010	3	Y	Y	N	Y	CD	Y	Y	NR	N	Y	N	NA	Y
Ramarakoto CE *et al.* 2008	3	Y	Y	N	Y	Y	Y	Y	NR	N	Y	N	NA	Y
Rasendramino MH *et al.* 1998	2	Y	Y	Y	Y	Y	Y	Y	NR	CD	Y	N	NA	Y
Reimert CM *et al.* 2000	2	Y	Y	Y	Y	Y	Y	Y	NR	N	Y	N	NA	Y
Ruiz-Guevara R *et al.* 2007	3	Y	Y	Y	Y	Y	Y	Y	NR	N	Y	N	NA	Y
Sarda RK *et al.* 1987	1	Y	Y	Y	Y	Y	Y	Y	NR	Y	N	N	NA	Y
Sissoko MS *et al.* 2009	1	Y	Y	Y	Y	Y	Y	Y	Y	Y	Y	N	NA	Y
Sleigh AC *et al.* 1986	4	Y	Y	Y	Y	Y	Y	Y	NR	N	Y	Y	NA	Y
Stephenson LS *et al.* 1984	1	Y	Y	Y	Y	Y	Y	Y	NR	N	Y	N	NA	Y
Stephenson LS *et al.* 1985	1	Y	Y	Y	Y	Y	Y	Y	Y	CD	Y	N	NA	Y
Stephenson LS *et al.* 1985	1	Y	Y	Y	Y	Y	Y	Y	Y	Y	Y	N	NA	Y
Stephenson LS *et al.* 1989	1	Y	Y	Y	Y	Y	Y	Y	Y	Y	Y	N	NA	Y
Stete K *et al.* 2012	3	Y	Y	Y	Y	Y	Y	Y	NA	Y	Y	N	NA	Y
Sukwa *et al.* 1987<br>Sukwa *et al.* 1988	2	Y	Y	Y	Y	Y	Y	Y	Y	N	N	N	NA	Y
Sukwa *et al.* 1993	1	Y	Y	Y	Y	Y	Y	Y	Y	Y	Y	N	NA	Y
Tohon ZB *et al.* 2008	2	Y	Y	Y	Y	Y	CD	Y	NR	Y	Y	N	NA	CD
Traore M *et al.* 1998	2	Y	Y	Y	Y	Y	Y	Y	Y	N	Y	N	NA	Y

Vennervald BJ *et al.* 2005	3	Y	Y	N	Y	Y	Y	Y	NR	N	Y	N	NA	Y
Wagatsuma Y *et al.* 1999	2	Y	Y	Y	Y	Y	Y	Y	NR	Y	Y	N	NA	Y
Wiest PM *et a.* 1994	4	Y	Y	Y	Y	Y	Y	Y	NR	N	Y	N	NA	Y
Zhao *et al.* 1995	2	Y	Y	Y	Y	Y	Y	Y	Y	N	N	N	NA	Y

Abbreviations: Y= Yes; N=No; NA= Not Applicable; NR= Not reported; CD= Could not determine

*Study Design Codes: 1 = Randomized placebo-controlled trial or drug comparison (RCT); 2 = Interventional trial with cohort longitudinal follow up of the same individuals (Cohort study); 3 = Selected subject intervention trial with follow up of the same individuals (Selected patient cohort study, less generalizable); 4 = Population-based intervention trial with one or more cross-sectional surveys before and after treatment (Before/after design; quasi-experimental)

Question codes:

1: Was the study question or objective clearly stated?

2: Were eligibility/selection criteria for the study population pre-specified and clearly described?

3: Were the participants in the study representative of those who would be eligible for the intervention in the general population of interest? (We scored Yes only for the studies that targeted all school age children or the entire population)

4= Were all eligible participants that met the pre-specified entry criteria enrolled?

5= Was the sample size sufficiently large to provide confidence in the findings? (We scored Yes when the studies presented their sample size calculations or when the study selected all of the eligible population in the recruited area - a common situation in the parasitic studies)

6= Was the intervention clearly described and delivered consistently across the study population?

7= Were the outcome measures pre-specified, clearly defined, valid, reliable, and assessed consistently across all study participants?

8= Were the people assessing the outcomes blinded to the participants' exposures/interventions?

9= Was the loss to follow-up after baseline 20% or less? Were those lost to follow-up accounted for in the analysis? (The first follow-up time was considered for this criterion when more than one follow-up was performed. The same criterion was adopted for the global meta-analysis of each morbidity)

10= Did the statistical methods examine changes in outcome measures from before to after the intervention? Were statistical tests done that provided p values for the pre-to-post changes?

11= Were outcome measures of interest taken multiple times before the intervention and multiple times after the intervention (i.e., did they use an interrupted time-series design)?

12= If the intervention was conducted at a group level (e.g., a whole hospital, a community, etc.) did the statistical analysis take into account the use of individual-level data to determine effects at the group level?

13= The distribution strategy of deworming medication was clearly described and delivered to at least 75% of the target population?
