# Supplementary material for: Decline in infection-related morbidities following drug-mediated reductions in the intensity of Schistosoma infection: A systematic review and meta-analysis
Source: PLoS Negl Trop Dis. 2017 Feb 17;11(2):e0005372. doi: 10.1371/journal.pntd.0005372 (PMC5333910; doi:10.1371/journal.pntd.0005372)
Supplement: S3 Text — Tables A-K indicating the results of subgroup analysis of pre- and post-treatment morbidity prevalence. (PDF) [file pntd.0005372.s005.pdf]

S3 Table A. Analysis of the prevalence of hepatomegaly before and after treatment, stratified by subgroups

**Subgroup**	**Study (N)**	**Association**		**Heterogeneity test**			**Study References (see listing pp. 7-10)**
		**OR (95% CI)**	**P value (Z test)**	**Q**	**P value**	$\mathbf{I^2}$	
**Right hepatic lobe**							
Clinical diagnosis	8	0,54 (0,29-1,00)	0,053	57,01	<0,001	87,72%	[31,52,53,56,57,64]
Ultrasound diagnosis	2	0,44 (0,21-0,95)	**0,036**	10,78	<0,001	90,73%	[10,63]
Subjects of all ages	4	0,66 (0,36-1,22)	0,187	53,21	<0,001	94,36%	[63,10,56,64]
School age subjects	6	0,39 (0,17-0,90)	**0,018**	20,81	0,001	75,98%	[53,57, 31,52]
Endpoint (0-12 months)	8	0,55 (0,30-1,01)	0,054	54,65	<0,001	87,19%	[53,63,64,57,31,52]
Endpoint (13-24 months)	3	0,43 (0,30-0,60)	**<0,001**	7,79	0,020	74,33%	[10,56,63]
Endpoint (>25 months)	2	0,22 (0,13-0,38)	**<0,001**	3,69	0,055	72,93%	[10,58]
S.mansoni	3	0,24 (0,06-0,93)	**0,039**	6,84	0,033	70,77%	[56,57]
S.japonicum	3	0,69 (0,28-1,65)	0,408	50,40	<0,001	96,03%	[10,63,64]
S.haematobium	3	0,84 (0,46-1,54)	0,585	3,48	**0,175**	42,61%	[52,53]
S.m/S.h	1	0,15 (0,06-0,36)	**<0,001**	-	-	-	[31]
All subjects infected (YES)	5	0,51 (0,22-1,16)	0,111	12,46	0,014	69,14%	[53,57,52]
All subjects infected (NO)	5	0,52 (0,29-0,96)	**0,038**	64,97	<0,001	93,84%	[10,31,56,63,64]
China	3	0,69 (0,28-1,65)	0,408	50,40	<0,001	96,03%	[10,63,64]
East Africa	3	0,84 (0,46-1,54)	0,585	3,48	**0,175**	42,61%	[52,53]
West Africa	1	0,15 (0,06-0,36)	**<0,001**	-	-	-	[31]
South Africa	3	0,24 (0,06-0,93)	**0,039**	6,84	0,033	70,77%	[56,57]
**Left hepatic lobe**							
Clinical diagnosis	4	0,33 (0,21-0,53)	**<0,001**	17,05	0,001	82,40%	[56,64,57]
Ultrasound diagnosis	6	0,59 (0,33-1,06)	0,080	47,56	<0,001	89,48%	[17,63,10,35,23,31]
Subjects of all ages	5	0,53 (0,33-0,77)	**0,001**	29,02	<0,001	86,22%	[63,10,35,56,64]
School age subjects	5	0,37 (0,15-0,89)	**0,027**	41,15	<0,001	90,28%	[17,57,23,31]
Endpoint (0-12 months)	7	0,38 (0,23-0,61)	**<0,001**	50,65	<0,001	88,15%	[17,63,64,57,23,31]
Endpoint (13-24 months)	6	0,59 (0,36-0,96)	**0,034**	49,16	<0,001	89,83%	[17,63,10,35,56,23]
Endpoint (>25 months)	3	0,36 (0,05-2,47)	0,303	97,79	<0,001	97,95%	[10,34,58]
S.mansoni	5	0,44 (0,23-0,85)	**0,015**	38,98	<0,001	89,73%	[17,23,56,57]
S.japonicum	4	0,53 (0,32-0,87)	**0,013**	28,55	<0,001	89,49%	[10,35,63,64]
S.m/S.h	1	0,16 (0,03-0,73)	**0,018**	-	-	-	[31]
All subjects infected (YES)	3	0,28 (0,13-0,63)	**0,002**	11,20	0,004	82,14%	[17,57]
All subjects infected (NO)	7	0,57 (0,38-0,85)	**0,006**	48,82	<0,001	87,71%	[63,10,35,56,64,23, 31]
China	4	0,53 (0,32-0,87)	**0,013**	28,55	<0,001	89,49%	[10,35,63,64]
South Africa	3	0,28 (0,13-0,61)	**0,001**	15,58	<0,001	87,16%	[56,57]
East Africa	1	1,25 (0,82-1,90)	0,287	-	-	-	[23]
West Africa	1	0,16 (0,03-0,73)	**0,018**	-	-	-	[31]
Egypt / Sudan	1	0,60 (0,34-1,05)	0,074	-	-	-	[17]
**Lobe not specified**							
Endpoint (0-12 months)	6	0,40 (0,18-0,89)	**0,026**	140	<0,001	96,42%	[8,19,20,50]
Endpoint (13-24 months)	5	0,45 (0,18-1,15)	0,097	142	<0,001	97,19%	[8,19,50]
Endpoint (>25 months)	8	0,32 (0,14-0,75)	**0,009**	209	<0,001	96,65%	[8,14,19,22,47,50]
Subjects of all ages	6	0,55 (0,30-1,02)	0,060	71,09	<0,001	92,96%	[14,19,20,47,50]
School age subjects	2	0,13 (0,09-0,20)	**<0,001**	0,29	**0,585**	0%	[8]
Specific subjects	1	0,22 (0,09-0,54)	**0,001**	-	-	-	[22]

All subjects infected (YES)	4	0,29 (0,12-0,67)	**0,004**	28,6	<0,001	89,5%	[8,20,47]
All subjects infected (NO)	5	0,45 (0,21-0,96)	**0,040**	72,4	<0,001	94,4%	[14,19,22,50]
East da África	5	0,35 (0,13-0,92)	**0,034**	136	<0,001	97,06%	[8,14,19]
South America	2	0,41 (0,12-1,44)	0,167	7,87	0,005	87,3%	[47,50]
Indonesia	1	0,49 (0,31-0,77)	**0,002**	-	-	-	[20]
Egypt / Sudan	1	0,22 (0,09-0,54)	**0,001**	-	-	-	[22]

S3 Table B. Analysis of the prevalence of splenomegaly before and after treatment, stratified by subgroups

**Subgroup**	**Study (N)**	**Association**		**Heterogeneity test**			**Study References (see listing pp. 7-10)**
		**OR (95% CI)**	**P value (Z test)**	**Q**	**OR (95% CI)**	**P value (Z test)**	
Clinical diagnosis	16	0,67 (0,50-0,90)	**0,009**	98,80	<0,001	84,81%	[47,50,52,53,22,14,56,64,57,11, 31,19,20]
Ultrasound diagnosis	5	0,52(0,19-1,38)	0,192	54,73	<0,001	92,69%	[12,17,63,10,35]
Subjects of all ages	11	0,61 (0,43-0,87)	**0,007**	89,97	<0,001	86,66%	[10,14,19,20,35,47,50,56,63,64]
School age subjects	8	0,66 (0,37-1,19)	0,171	67,78	<0,001	89,77%	[11,17,31,52,53,57]
Specific subjects	2	0,53 (0,30-0,92)	**0,025**	0,102	**0,750**	0%	[12,22]
Endpoint (0-12 months)	13	0,78 (0,58-1,06)	0,125	85,83	<0,001	86,01%	[11,17,19,20,31,50,52,53,57,64]
Endpoint (13-24 months)	9	0,47 (0,27-0,81)	**0,007**	137,04	<0,001	94,16%	[10,17,19,35,56, 63]
Endpoint (>25 months)	8	0,46 (0,26-0,79)	**0,006**	40,55	<0,001	82,73%	[10,12,14,19,22,47,50]
S.mansoni	12	0,65 (0,47-0,88)	**0,007**	46,05	<0,001	76,11%	[12,17,47,50,22,14,56,57,11,19]
S.japonicum	5	0,56 (0,26-1,23)	0,152	55,54	<0,001	92,79%	[63,10,35,64,20]
S.haematobium	3	1,13 (0,49-2,50)	0,797	15,93	<0,001	87,45%	[52,53]
S.m/S.h	1	0,26 (0,18-0,37)	**<0,001**	-	-	-	[31]
All subjects infected (YES)	9	0,74 (0,46-1,19)	0,215	35,9	<0,001	77,71%	[12,17,47,52,53,57,11]
All subjects infected (NO)	12	0,57 (0,38-0,83)	**0,004**	121	<0,001	90,94%	[50,63,10,22,35,14,56,64,31,19, 20]
China	4	0,47 (0,17-1,32)	0,156	46,75	<0,001	93,58%	[10,35,63,64]
East Africa	6	0,97 (0,67-1,42)	0,906	25,19	<0,001	80,15%	[14,19,52,53]
Egypt/Sudan	2	0,81 (0,35-1,87)	0,627	5,25	0,022	80,96%	[17,22]
South Africa	3	0,42 (0,26-0,68)	**<0,001**	3,92	**0,140**	49,05%	[56,57]
South America	3	0,61 (0,36-1,03)	0,066	0,88	**0,644**	0%	[12,47,50]
Caribbean	1	1,00 (0,12-8,12)	1,000	-	-	-	[11]
Indonesia	1	1,05 (0,73-1,50)	0,784	-	-	-	[20]

S3 Table C. Analysis of the prevalence of periportal fibrosis before and after treatment, stratified by subgroups

Subgroup	Study (N)	Association		Heterogeneity test			Study References (see listing pp. 7-10)
		OR (95% CI)	P value (Z test)	Q	P value	I^2	
Subjects of all ages	7	0,52 (0,39-0,69)	**<0,001**	17,92	0,006	66,48%	[10,18,35,36,47,63]
School age subjects	1	0,48 (0,33-0,68)	**<0,001**	-	-	-	[23]
Specific subjects	4	0,04 (0,003-0,67)	**0,025**	16,97	0,001	82,32%	[4,12,22,43]
Endpoint (0-12 months)	5	0,59 (0,43-0,81)	**0,001**	12,53	0,014	68,09%	[17,18,22,36,63]
Endpoint (13-24 months)	5	0,66 (0,47-0,93)	**0,017**	10,53	0,032	62,03%	[10,17,22,35,63]
Endpoint (>25 months)	9	0,28 (0,14-0,54)	**<0,001**	44,74	<0,001	82,12%	[4,10,12,18,22,35, 43,47]
S.mansoni	9	0,35 (0,23-0,54)	**<0,001**	27,91	<0,001	71,34%	[4,12,17,18,22,36, 43,47]
S.japonicum	3	0,67 (0,41-1,09)	0,113	8,91	0,012	77,56%	[10,35,63]
All subjects infected (YES)	5	0,52 (0,36-0,75)	**<0,001**	14,15	0,007	71,73%	[12,17,36,43,47]
All subjects infected (NO)	7	0,33 (0,17-0,65)	**0,002**	23,51	0,001	74,48%	[4,10,18,22,35,63]
China	3	0,67 (0,41-1,09)	0,113	8,91	0,012	77,56%	[10,35,63]
East Africa	3	0,32 (0,14-0,74)	**0,008**	12,95	0,002	84,55%	[4,18]
Egypt/Sudan	3	0,10 (0,008-1,32)	0,081	9,10	0,011	78,02%	[17,22,10]
South America	3	0,38 (0,16-0,87)	**0,022**	5,67	**0,059**	64,74%	[12,36,47]

S3 Table D. Analysis of the prevalence of dilated portal vein before and after treatment, stratified by subgroups

Subgroup	Study (N)	Association		Heterogeneity test			Study References (see listing pp. 7-10)
		OR (95% CI)	P value (Z test)	Q	P value	I^2	
Subjects of all ages	2	0,99 (0,49-2,00)	0,986	3,83	0,05	73,94%	[10,35]
School age subjects	1	0,10 (0,03-0,30)	**<0,001**	-	-	-	[23]
Specific subjects	1	0,79 (0,30-2,05)	0,628	-	-	-	[61]
S. mansoni	2	0,29 (0,04-2,10)	0,222	3,83	0,050	73,94%	[23,61]
S. japonicum	2	0,99 (0,49-2,00)	0,986	7,66	0,006	86,95%	[10,35]
Endpoint (0-12 months)	3	0,29 (0,04-2,10)	0,222	7,66	0,006	86,95%	[23,61]
Endpoint (13-24 months)	4	0,59 (0,25-1,40)	0,236	19,31	<0,001	84,46%	[10,35,61,23]
Endpoint (>25 months)	2	0,34 (0,17-1,03)	0,057	1,83	**0,175**	45,58%	[34,61]
Treatment 1x	4	0,57 (0,23-1,42)	0,232	20,72	<0,001	85,53%	[10,35,61,23]
Treatment 2x	3	0,26 (0,10-0,62)	**0,002**	4,61	**0,099**	56,67%	[12,61,23]

S3 Table E. Analysis of the prevalence of diarrhea before and after treatment, stratified by subgroups

Subgroup	Study (N)	Association		Heterogeneity test			Study References (see listing pp. 7-10)
		OR (95% CI)	P value (Z test)	Q	P value	I^2	
Subjects of all ages	4	0,50 (0,34-0,74)	**0,001**	21,42	<0,001	85,9%	[56,30,19]
School age subjects	2	0,06 (0,02-0,15)	**<0,001**	0,46	**0,494**	0%	[11,42]
Specific population	1	0, 69 (0,50-0,93)	**0,018**	-	-	-	[5]
Endpoint (0-12 months)	5	0,44 (0,26-0,73)	**0,002**	28,13	<0,001	85,78%	[5,11,19,42]
Endpoint (13-24 months)	5	0,60 (0,32-1,13)	0,115	86,98	<0,001	95,40%	[5,56,30,19]
Endpoint (>25 months)	2	0,39 (0,26-0,57)	**<0,001**	2,93	**0,087**	65,90%	[19]
All subjects infected (YES)	3	0,13 (0,03-0,53)	**0,004**	12,03	0,002	83,38%	[11,30,42]
All subjects infected (NO)	4	0,60 (0,44-0,81)	**0,001**	13,66	0,003	78,04%	[5,56,19]
East Africa	3	0,70 (0,60-0,82)	**<0,001**	0,51	**0,771**	0%	[5,19]
Caribbean	1	0,11 (0,01-0,65)	**0,016**	-	-	-	[11]
South America	1	0,05 (0,02-0,15)	**<0,001**	-	-	-	[42]
South Africa	1	0,37 (0,27-0,50)	**<0,001**	-	-	-	[56]
West Africa	1	0,33 (0,23-0,47)	**<0,001**	-	-	-	[30]

S3 Table F. Analysis of the prevalence of blood in stool before and after treatment, stratified by subgroups

Subgroup	Study (N)	Association		Heterogeneity test			Study References (see listing pp. 7-10)
		OR (95% CI)	P value (Z test)	Q	P value	I^2	
Subjects of all ages	4	0,21 (0,08-0,57)	**0,002**	51,16	<0,001	94,13%	[7,56,30,64]
School age subjects	3	0,27 (0,17-0,41)	**< 0,001**	3,48	**0,175**	42,64%	[57,42]
Specific population	1	0,48 (0,28-0,82)	**0,007**	-	-	-	[5]
Endpoint (0-12 months)	4	0,31 (0,21-0,46)	**< 0,001**	6,42	**0,093**	53,29%	[5,57,42]
Endpoint (13-24 months)	5	0,29 (0,13-0,67)	**0,004**	64,86	<0,001	93,83%	[5,7,56,30,64]
Endpoint (>25 months)	1	0,27 (1,16-0,44)	**< 0,001**	-	-	-	[7]
All subjects infected (YES)	4	0,22 (0,14-0,35)	**< 0,001**	8,23	0,041	63,55%	[5,7,56,64]
All subjects infected (NO)	4	0,29 (0,12-0,67)	**0,004**	34,39	<0,001	91,27%	[57,30,42]
East Africa	2	0,26 (0,08-0,88)	**0,031**	8,88	0,003	88,74%	[5,7]
South Africa	3	0,25 (0,15-0,40)	**< 0,001**	5,31	**0,070**	62,37%	[56,57]
China	1	0,68 (0,52-0,89)	**0,006**	-	-	-	[64]
South America	1	0,13 (0,05-0,34)	**< 0,001**	-	-	-	[42]
West Africa	1	0,15 (0,10-0,24)	**< 0,001**	-	-	-	[30]

S3 Table G. Analysis of the prevalence of blood in urine before and after treatment, stratified by subgroups

Subgroup	Study (N)	Association		Heterogeneity test			Study References (see listing pp. 7-10)
		**OR (95% CI)**	**P value (Z test)**	**Q**	**P value**	**I^2**	
Subjects of all ages	5	0,16 (0,12-0,22)	**<0,001**	16,73	0,002	76,10%	[39,45,25,13,62]
School age subjects	11	0,07 (0,04-0,11)	**<0,001**	171,93	<0,001	94,18%	[24,26,32,54,55,49, 59,29,48,9]
Specific population	1	0,002 (0,00-0,03)	**<0,001**	-	-	-	[38]
All subjects infected (YES)	10	0,07 (0,04-0,10)	**<0,001**	70,93	<0,001	87,31%	[26,38,39,54,55,49,13, 62,48]
All subjects infected (NO)	7	0,14 (0,07-0,26)	**<0,001**	142,54	<0,001	95,79%	[24,32,45,25,59,29,9]
Endpoint (0-6 months)	11	0,05 (0,03-0,10)	**<0,001**	127,50	<0,001	92,15%	[9,13,24,25,38,39,48, 49,54,55,62]
Endpoint (7-12 months)	6	0,12 (0,06-0,21)	**<0,001**	147,9	<0,001	96,62%	[26,29,32,45,59]
Endpoint (>12 months)	3	0,08 (0,05-0,15)	**<0,001**	6,09	0,048	67,16%	[9,24,62]
East Africa	9	0,08 (0,04-0,16)	**<0,001**	183,87	<0,001	95,64%	[24,26,38,54,45,25, 29,48]
West Africa	8	0,09 (0,06-0,13)	**<0,001**	51,31	<0,001	86,35%	[32,39,55,49,13,59, [62,9]

S3 Table H. Analysis of the prevalence of protein in urine before and after treatment, stratified by subgroups

Subgroup	Study (N)	Association		Heterogeneity test			Study References (see listing pp. 7-10)
		**OR (95% CI)**	**P value (Z test)**	**Q**	**P value**	**I^2**	
Subjects of all ages	4	0,12 (0,03-0,45)	**<0,001**	192,47	0,002	98,45%	[25,39,45,62]
School age subjects	6	0,11 (0,05-0,24)	**<0,001**	115,12	<0,001	95,65%	[26,29,48,54,55]
Specific population	1	0,17 (0,08-0,40)	**<0,001**	-	-	-	[38]
All subjects infected (YES)	8	0,05 (0,02-0,10)	**<0,001**	122,1	<0,001	97,01%	[26,38,39,48,54,55, 62]
All subjects infected (NO)	3	0,48 (0,15-1,52)	0,216	67	<0,001	94,26%	[25,29,45]
Endpoint (0-6 months)	7	0,05 (0,01-0,21)	**<0,001**	242,5	<0,001	97,52%	[25,38,39,48,54,55, 62]
Endpoint (7-12 months)	4	0,24 (0,12-0,48)	**<0,001**	72,83	<0,001	95,88%	[26,29,45]
Endpoint (>12 months)	1	0,03 (0,02-0,04)	**<0,001**	-	-	-	[62]
East Africa	8	0,16 (0,08-0,32)	**<0,001**	185,93	<0,001	96,23%	[25,26,29,38,45,48, 54]
West Africa	3	0,04 (0,02-0,08)	**<0,001**	7,89	<0,001	74,68%	[39,55,62]

S3 Table I. Analysis of the prevalence of ultrasound abnormalities in the urinary bladder before and after treatment, stratified by subgroups

Subgroup	Study (N)	Association		Heterogeneity test			Study References (see listing pp. 7-10)
		OR (95% CI)	P value (Z test)	Q	P value	I²	
Subjects of all ages	4	0,25 (0,13-0,49)	**<0,001**	34,31	0,002	91,25%	[13,15,45,60]
School age subjects	7	0,18 (0,12-0,26)	**<0,001**	25,11	<0,001	76,10%	[9,24,27,28,46,59]
Specific population	4	0,009 (0,001-0,09)	**<0,001**	32,89	<0,001	90,87%	[16,44,21]
All subjects infected (YES)	9	0,07 (0,03-0,16)	**<0,001**	54,19	<0,001	85,23%	[13,15,16,24,28, 44,21]
All subjects infected (NO)	6	0,21 (0,13-0,34)	**<0,001**	60,21	<0,001	91,69%	[9,27,45,46,59,60]
Endpoint (0-6 months)	9	0,10 (0,06-0,18)	**<0,001**	40,38	<0,001	80,19%	[9,13,24,28,44,46, 21]
Endpoint (7-12 months)	8	0,20 (0,13-0,31)	**<0,001**	70,26	<0,001	90,03%	[15,16,24,27,45, 46,59,60]
Endpoint (>12 months)	3	0,39 (0,19-0,80)	**<0,001**	12,27	0,002	83,70%	[9,24,46]
East Africa	9	0,11 (0,07-0,19)	**<0,001**	47,65	<0,001	83,21%	[24,27,28,44,45, 46,21]
West Africa	5	0,23 (0,13-0,39)	**<0,001**	37,90	<0,001	89,44%	[9,13,15,59,60]
Central Africa	1	<0,001 (<0,001-0,008)	**<0,001**	-	-	-	[16]

S3 Table J. Analysis of the prevalence of ultrasound abnormalities in the upper urinary tract before and after treatment, stratified by subgroups

Subgroup	Study (N)	Association		Heterogeneity test			Study References (see listing pp. 7-10)
		OR (95% CI)	P value (Z test)	Q	P value	I²	
Subjects of all ages	4	0,24 (0,18-0,32)	**<0,001**	0,551	**0,908**	0%	[13,15,45,60]
School age subjects	5	0,32 (0,13-0,81)	**0,017**	36,14	<0,001	88,93%	[9,27,28,59]
All subjects infected (YES)	4	0,34 (0,20-0,57)	**<0,001**	5,51	**0,138**	45,58%	[13,15,28]
All subjects infected (NO)	5	0,24 (0,09-0,64)	**0,004**	48,51	<0,001	91,75%	[9,27,45,59,60]
Endpoint (0-6 months)	3	0,46 (0,29-0,72)	**0,001**	1,12	**0,570**	0%	[13,28]
Endpoint (7-12 months)	5	0,27 (0,11-0,64)	**0,003**	48,93	<0,001	91,82%	[15,27,45,59,60]
Endpoint (>12 months)	1	0,12 (0,04-0,38)	**<0,001**	-	-	-	[9]
East Africa	4	0,52 (0,26-1,04)	0,068	17,17	<0,001	82,53%	[27,28,45]
West Africa	5	0,18 (0,11-0,28)	**<0,001**	6,49	**0,165**	38,39%	[9,13,15,59,60]

S3 Table K. Analysis of blood hemoglobin before and after treatment, stratified by subgroups

Subgroup	Study (N)	Effect size		Heterogeneity test			Study References (see listing pp. 7-10)
		SMD (95% CI)	P value (Z test)	Q	P value	I²	
Placebo	4	-0,88 (-3,09/1,31)	0,430	597,1	<0,001	99,49%	[3,37,51,52]
Schist.*	7	0,59 (-0,02/1,22)	0,060	213,8	<0,001	97,19%	[2,6,37,52,51,40]
Schist./Helmin.*	5	0,62 (-0,91/2,15)	0,427	2.431	<0,001	99,83%	[3,6,32,49,23]
Endpoint (0-6 months)	7	0,29 (-0,43/1,02)	0,427	368,4	<0,001	98,3%	[2,3,6,37,49,51]
Endpoint (7-12 months)	5	1,04 (-0,61/2,69)	0,216	2207	<0,001	99,81%	[32,52,40,23]
Endpoint (>12 months)	3	0,42 (0,175/0,666)	**0,001**	8,65	0,013	76,89%	[6,23]
Venous blood	5	1,37 (-0,28/3,03)	0,103	939,2	<0,001	99,57%	[32,37,51,52]
Capilary blood	7	0,06 (-0,22/0,35)	0,672	114,1	<0,001	94,74%	[2,3,6,49,40,23]

Full reference list of papers cited in S3 Tables A-K

1. Awad El Karim MA, Collins KJ, Sukkar MY, Omer AHS, Amin MA, Doré C. An assessment of anti-schistosomal treatment on physical work capacity. J Trop Med Hyg. 1981; 84:67-72.
2. Ayoya MA, Spiekermann-Brouwer GM, Traore AK, Stoltzfus RJ, Habicht JP, Garza C. Multiple micronutrients including iron are not more effective than iron alone for improving hemoglobin and iron status of Malian school children. J Nutr. 2009;139(10):1972–9.
3. Beasley NMR, Tomkins AM, Hall A, Kihamia CM, Lorri W, Nduma B, et al. The impact of population level deworming on the haemoglobin levels of schoolchildren in Tanga, Tanzania. Trop Med Int Heal. 1999;4(11):744–50.
4. Berhe N, Myrvang B, SG G. Reversibility of schistosomal periportal thickening/fibrosis after praziquantel therapy: a twenty-six month follow-up study in Ethiopia. Am J Trop Med Hyg. 2008;78(2):228–34.
5. Betson M, Sousa-Figueiredo JC, Kabatereine NB, Stothard JR. Use of fecal occult blood tests as epidemiologic indicators of morbidity associated with intestinal schistosomiasis during preventive chemotherapy in young children. Am J Trop Med Hyg. 2012;87(4):694–700.
6. Bhargava A, Jukes M, Lambo J, Kihamia CM, Lorri W, Nokes C, et al. Anthelmintic treatment improves the hemoglobin and serum ferritin concentrations of Tanzanian schoolchildren. Food Nutr Bull. 2003;24(4):332–42.
7. Boisier P, CE R, VE R, Rabarijaona L, Serieye J, Roux J, et al. Reversibility of Schistosoma mansoni-associated morbidity after yearly mass praziquantel therapy: ultrasonographic assessment. Trans R Soc Trop Med Hyg. 1998;92(4):451–3.
8. Butterworth AE, Sturrock RF, Ouma JH, Mbugua GG, Fulford AJC, Kariuki HC, et al. Comparison of different chemotherapy strategies against schistosoma-mansoni in Machakos district, Kenya - effects on human infection and morbidity. Parasitology. 1991;103(3):339–55.
9. Campagne G, Garba a, Barkiré H, Vera C, Sidiki a, Chippaux JP. Continued ultrasonic follow-up of children infected with Schistosoma haematobium after treatment with praziquantel. Trop Med Int Health. 2001;6(1):24–30.
10. Carlton EJ, Hsiang M, Zhang Y, Johnson S, Hubbard A, Spear RC. The impact of Schistosoma japonicum infection and treatment on ultrasound-detectable morbidity: a five-year cohort study in Southwest China. PLoS Negl Trop Dis. 2010;4(5):e685.
11. Cook JA, Jordan P, Woodstock L, Pilgrim V. A controlled trial of hycanthone and placebo in schistosomiasis mansoni in St. Lucia. Ann Trop Med Parasitol. 1977;71(2):197-203.
12. Cota GF, Pinto-Silva RA, Antunes CMF, Lambertucci JR. Ultrasound and clinical investigation of hepatosplenic schistosomiasis: Evaluation of splenomegaly and liver fibrosis four years after mass chemotherapy with oxamniquine. Am J Trop Med Hyg. 2006;74(1):103–7.
13. Delegue P, Picquet M, DJ S, Vercruysse J, Sambou B, Ly A. Morbidity induced by Schistosoma haematobium infections, as assessed by ultrasound before and after treatment with praziquantel, in a recently expanded focus (Senegal River basin). Ann Trop Med Parasitol. 1998;92(7):775–83.
14. DeStigter KV, King CH, Keating CE, Ouma JH, Siongok TK, Mahmoud AF. Effects of targeted mass treatment on intensity of infection and morbidity in scistosomiasis mansoni: seven-year follow-up of a community in machakos, kenya. Trans Assoc Am Physicians. 1989;102:209–12.
15. Devidas A, Lamothe F, Develoux M, Mouchet F, Sellin B. Ultrasonographic assessment of the regression of bladder and renal lesions due to Schistosoma haematobium after treatment with praziquantel. Ann Soc Belg Med Trop. 1989;69(1):57–65.
16. Doehring E, Ehrich JH, Bremer HJ. Reversibility of urinary tract abnormalities due to Schistosoma haematobium infection. Kidney Int. 1986;30(4):582–5.
17. Doehring-Schwerdtfeger E, Abdel-Rahim IM, Kardorff R, Kaiser C, Franke D, Schlake J, et al. Ultrasonographical investigation of periportal fibrosis in children with Schistosoma mansoni infection: Reversibility of morbidity twenty-three months after treatment with praziquantel. Am J Trop Med Hyg. 1992;46(4):409–15.
18. Frenzel K, Grigull L, Odongo-Aginya E, Ndugwa CM, Loroni-Lakwo T, Schweigmann U, et al. Evidence for a long-term effect of a single dose of praziquantel on Schistosoma mansoni-induced hepatosplenic lesions in northern Uganda. Am J Trop Med Hyg. 1999;60(6):927–31.

19. Gryseels B, Nkulikyinka L, Engels D. Impact of repeated community-based selective chemotherapy on morbidity due to schistosomiasis mansoni. Am J Trop Med Hyg. 1994;51(5):634–41.
20. Hadidjaja P, Syamsuddin N, Ismid IS, Sudomo M, Campbell J, Putrali J. The impact of schistosomiasis mass treatment on hepato-splenomegaly in Napu Valley, Central Sulawesi, Indonesia. Southeast Asian J Trop Med Public Health. 1985;16(3):401–4.
21. Hatz C, Mayombana C, Savigny D, MacPherson CNL, Koella JC, Degrémont A, Tanner M. Ultrasound scanning for detecting morbidity due to Schistosoma haematobium and its resolution following treatment with different doses of praziquantel. Trans R Soc Trop Med Hyg. 1990;84:84-88.
22. Homeida MA, el Tom I, Nash T, Bennett JL. Association of the therapeutic activity of praziquantel with the reversal of Symmers' fibrosis induced by Schistosoma mansoni. Am J Trop Med Hyg. 1991;45(3):360–5.
23. Kabatereine NB, Brooker S, Koukounari A, Kazibwe F, Tukahebwa EM, Fleming FM, et al. Impact of a national helminth control programme on infection and morbidity in Ugandan schoolchildren. Bull World Health Organ. 2007;85(2):91–9.
24. Kahama AI, Odek AE, Kihara RW, Vennervald BJ, Kombe Y, Nkulila T, et al. Urine circulating soluble egg antigen in relation to egg counts, hematuria, and urinary tract pathology before and after treatment in children infected with Schistosoma haematobium in Kenya. Am J Trop Med Hyg. 1999;61(2):215–9.
25. Kiliku FM, Kimura E, Muhoho N, Migwi DK, Katsumata T. The usefulness of urinalysis reagent strips in selecting Schistosoma haematobium egg positives before and after treatment with praziquantel. J Trop Med Hyg. 1991;94(6):401–6.
26. King CH, Lombardi G, Lombardi C, Greenblatt R, Hodder S, Kinyanjui H, et al. Chemotherapy-based control of schistosomiasis haematobia. II. Metrifonate vs. praziquantel in control of infection-associated morbidity. Am J Trop Med Hyg. 1990;42(6):587–95.
27. King CH, Lombardi G, Lombardi C, Greenblatt R, Hodder S, Kinyanjui H, et al. Chemotherapy-based control of schistosomiasis haematobia. I. Metrifonate versus praziquantel in control of intensity and prevalence of infection. Am J Trop Med Hyg. 1988;39(3):295–305.
28. King CH, Muchiri EM, Mungai P, Ouma JH, Kadzo H, Magak P, et al. Randomized comparison of low-dose versus standard-dose praziquantel therapy in treatment of urinary tract morbidity due to Schistosoma haema tobium infection. Am J Trop Med Hyg. 2002;66(6):725–30.
29. Kitange HM, Swai ABM, McLarty DG, Alberti KGMM. Schistosomiasis prevalence after administration of praziquantel to school children in Melela village, Morogoro region, Tanzania. East Afr Med J. 1993;70(12):782–6.
30. Kongs A, Verle P, Dieng A, Talla I, Rouquet P. Clinical investigation of a population recently infected with Schistosoma mansoni (Richard-Toll, Senegal). Trop Med Int Heal. 1996;1(2):191–8.
31. Koukounari A, Donnelly CA, Sacko M, Keita AD, Landouré A, Dembelé R, et al. The impact of single versus mixed schistosome species infections on liver, spleen and bladder morbidity within Malian children pre- and post-praziquantel treatment. BMC Infect Dis. 2010;10:227.
32. Koukounari A, Gabrielli AF, Toure S, Bosque-Oliva E, Zhang Y, Sellin B, et al. Schistosoma haematobium infection and morbidity before and after large-scale administration of praziquantel in Burkina Faso. J Infect Dis. 2007;196(5):659–69.
33. Latham MC, Stephenson LS, Hall A, Wolgemuth JC, Elliot TC, Crompton DET. Parasitic infections, anaemia and nutritional status: a study of their interrelationships and the effect of prophylaxis and treatment on workers in Kwale District, Kenya. Trans R Soc Trop Med Hyg. 1983;77(1):411-48.
34. Li YS, Sleigh a C, Li Y, Tanner M, Dessein a, Williams GM, et al. Five-year impact of repeated praziquantel treatment on subclinical morbidity due to Schistosoma japonicum in China. Trans R Soc Trop Med Hyg. 2002;96(4):438–43.
35. Li YS, Sleigh AC, Ross AG, Li Y, Williams GM, Tanner M, et al. Two-year impact of praziquantel treatment for Schistosoma japonicum infection in China: re-infection, subclinical disease and fibrosis marker measurements. Trans R Soc Trop Med Hyg. ENGLAND; 2000;94(2):191–7.

36. Martins-Leite P, Gazzinelli G, Alves-Oliveira LF, Gazzinelli A, Malaquias LCC, Correa-Oliveira R, et al. Effect of chemotherapy with praziquantel on the production of cytokines and morbidity associated with schistosomiasis mansoni. Antimicrob Agents Chemother. 2008;52(8):2780–6.
37. McGarvey ST, Aligui G, Graham KK, Peters P, Olds GR, Olveda R. Schistosomiasis japonica and childhood nutritional status in northeastern Leyte, the Philippines: a randomized trial of praziquantel versus placebo. Am J Trop Med Hyg. 1996;54(5):498–502.
38. Mekonnen A, Legesse M, Belay M, Tadesse K, Torben W, Teklemariam Z, et al. Efficacy of Praziquantel against Schistosoma haematobium in Dulshatalo village, western Ethiopia. BMC Res Notes. 2013;6(1):392.
39. Mott KE, Dixon H, Osei-Tutu E, England EC, Davis A. Effect of praziquantel on hematuria and proteinuria in urinary schistosomiasis. Am Soc Trop Med Hyg. 1985;34(6):1119–26.
40. Mwanakasale V, Siziya S, Mwansa J, Koukounari A, Fenwick A. Impact of iron supplementation on schistosomiasis control in Zambian school children in a highly endemic area. Malawi Med J. 2009;21(1):12–8.
41. Ndamba J, Makaza N, Munjoma M, Gomo E, Kaondera KC. The physical fitness and work performance of agricultural workers infected with Scgistosoma mansoni in Zimbabwe. Ann Trop Med Parasitol. 1993;87(6):553-561.
42. Peixinho EL, André B, Bina JC. Sintomatologia intestinal na fase crônica da esquistossomose mansoni. Rev Soc Bras Med Trop. 1986;19(1):27–30.
43. Rahoud SA, Mergani A, Khamis AH, Saeed OK, Mohamed-Ali Q, Dessein AJ, et al. Factors controlling the effect of praziquantel on liver fibrosis in Schistosoma mansoni-infected patients. FEMS Immunol Med Microbiol. 2010;58(1):106–12.
44. Ramarakoto CE, Leutscher PDC, van Dam G, Christensen NO. Ultrasonographical findings in the urogenital organs in women and men infected with Schistosoma haematobium in northern Madagascar. Trans R Soc Trop Med Hyg. 2008;102(8):767–73.
45. Rasendramino MH, Rajaona HR, Ramarakoto VE, Leutscher P, Cordonnier D, Esterre P. Effect du praziquantel sur les retentissements uro-néphrologiques de la bilharziose urinaire. Nephrologie. 1998;19(6):347–51.
46. Reimert CM, Mshinda HM, Hatz CF, Kombe Y, Nkulila T, Poulsen LK, et al. Quantitative assessment of eosinophiluria in Schistosoma haematobium infections: A new marker of infection and bladder morbidity. Am J Trop Med Hyg. 2000;62(1):19–28.
47. Ruiz-Guevara R, de Noya BA, Valero SK, Lecuna P, Garassini M, Noya O. Clinical and ultrasound findings before and after praziquantel treatment among Venezuelan schistosomiasis patients. Rev Soc Bras Med Trop. 2007;40(5):505–11.
48. Sarda RK, Kihamia CM, Minjas JN, Mahikwano LF. Haematuria and proteinuria in urinary schistosomiasis: response to therapy with praziquantel in Tanzanian children. Trop Med Parasitol. 1987;38(1):31–3.
49. Sissoko MS, Dabo A, Traore H, Diallo M, Traore B, Konate D, et al. Efficacy of artesunate + sulfamethoxypyrazine/pyrimethamine versus praziquantel in the treatment of Schistosoma haematobium in children. PLoS One. United States; 2009;4(10):e6732.
50. Sleigh AC, Mott KE, Hoff R, Maguire JH, da Fran??a Silva JT. Manson's schistosomiasis in Brazil: 11-year evaluation of successful disease control with oxamniquine. Lancet. 1986;1:635–7.
51. Stephenson LS Kurz KM, Kinoti SN, Oduori ML, Crompton DW. Relationships of Schistosoma hematobium, hookworm and malarial infections and metrifonate treatment to hemoglobin level in Kenyan school children. Am J Trop Med Hyg. 1985;34(3):519–28.
52. Stephenson LS, Kinoti SN, Latham MC, Kurz KM, Kyobe J. Single dose metrifonate or praziquantel treatment in Kenyan children. I. Effects on Schistosoma haematobium, hookworm, hemoglobin levels, splenomegaly, and hepatomegaly. Am-J-Trop-Med-Hyg. 1989;41(4):436–44.
53. Stephenson LS, Latham MC, Kinoti SN, Oduori ML. Regression of splenomegaly and hepatomegaly in children treated for Schistosoma haematobium infection. Am J Trop Med Hyg. 1985;34(1):119–23.
54. Stephenson LS, Latham MC, Kinoti SN, Oduori ML. Sensitivity and specificity of reagent strips in screening of Kenyan children for Schistosoma haematobium infection. Am J Trop Med Hyg. 1984;33(5):862–71.

55. Stete K, Krauth SJ, Coulibaly JT, Knopp S, Hattendorf J, Müller I, et al. Dynamics of Schistosoma haematobium egg output and associated infection parameters following treatment with praziquantel in school-aged children. Parasit Vectors. 2012;5(1):298.
56. Sukwa TY, Bulsara MK, Wurapa FK. Reduction in prevalence, intensity of infection and morbidity due to schistosoma mansoni infection in a community following treatment with praziquantel. J Trop Med Hyg. 1987;90(4):205-11.
57. Sukwa TY. A community-based randomized trial of praziquantel to control schistosomiasis morbidity in schoolchildren in Zambia. Ann Trop Med Parasitol. 1993;87(2):185–94.
58. Sukwa, Ba B, Fk W, Sukwa TY, Boatin B a, Wurapa FK. A three year follow-up of chemotherapy with praziquantel in a rural Zambian community endemic for schistosomiasis mansoni. Trans R Soc Trop Med Hyg. 1988;82(2):258–60.
59. Tohon ZB, Mainassara HB, Garba A, Mahamane AE, Bosqué-Oliva E, Ibrahim ML, et al. Controlling schistosomiasis: Significant decrease of anaemia prevalence one year after a single dose of praziquantel in Nigerien schoolchildren. PLoS Negl Trop Dis. 2008;2(5):e241.
60. Traore M, Traore HA, Kardorff R, Diarra A, Landoure A, Vester U, et al. The public health significance of urinary schistosomiasis as a cause of morbidity in two districts in Mali. Am J Trop Med Hyg. 1998;59(3):407–13.
61. Vennervald BJ, Booth M, Butterworth AE, Kariuki HC, Kadzo H, Ireri E, et al. Regression of hepatosplenomegaly in Kenyan school-aged children after praziquantel treatment and three years of greatly reduced exposure to Schistosoma mansoni. Trans R Soc Trop Med Hyg. 2005;99(2):150–60.
62. Wagatsuma Y, Aryeetey ME, Sack DA, Morrow RH, Hatz C, Kojima S. Resolution and resurgence of schistosoma haematobium-induced pathology after community-based chemotherapy in Ghana, as detected by ultrasound. J Infect Dis. 1999;179(6):1515–22.
63. Wiest PM, Wu G, Zhong S, McGarvey ST, Yuan J, Olveda RM, et al. Impact of annual screening and chemotherapy with praziquantel on schistosomiasis japonica on Jishan Island, People's Republic of China. Am J Trop Med Hyg. 1994;51(2):162–9.
64. Zhao G, Jiang Q, Wasley A, Zhang S, Wu Z, Liu Z, Yuan H. Changes in prevalence, intensity of infection and morbidity due to Schistosoma japonicum infection in a community following a single treatment with praziquantel. Trop Geogr Med. 1995;47(6):289-292.
