# Supplementary material for: Decline in infection-related morbidities following drug-mediated reductions in the intensity of Schistosoma infection: A systematic review and meta-analysis
Source: PLoS Negl Trop Dis. 2017 Feb 17;11(2):e0005372. doi: 10.1371/journal.pntd.0005372 (PMC5333910; doi:10.1371/journal.pntd.0005372)
Supplement: S1 Fig — Individual plots indicate, by morbidity, the results for each individual study included for analysis, and the summary OR and confidence interval estimated across all included studies. ORs and their confidence intervals for individual studies are shown numerically in the statistics columns, and graphically by the corresponding black boxes and black lines. The summary OR and confidence interval is indicated by the red diamond at the bottom of each plot. (PDF) [file pntd.0005372.s006.pdf]

Hepatomegaly-Left

Study name	Time point	Statistics for each study			
		Odds ratio	Lower limit	Upper limit	p-Value
Carlton 2010	24	0,957	0,667	1,373	0,812
Doehring-Schwerdtf. 1992	7	0,601	0,344	1,050	0,074
Kabatereine 2007	12	1,255	0,826	1,907	0,287
Koukounari 2010	12	0,165	0,037	0,738	0,018
Li YS 2000	24	0,660	0,408	1,068	0,091
Sukwa 1987	16	0,538	0,404	0,718	0,000
Sukwa1 1993	12	0,189	0,103	0,344	0,000
Sukwa2 1993	12	0,199	0,112	0,356	0,000
Wiest 1994	12	0,288	0,217	0,381	0,000
Zhao 1995	12	0,476	0,377	0,601	0,000
		0,468	0,327	0,671	0,000

Odds ratio and 95% CI

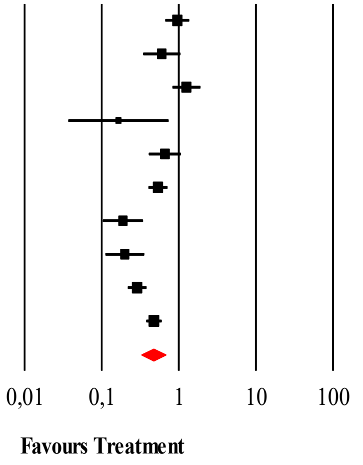

Effect estimation: Z = -4,134; p <0,001
Heterogeneity: Q = 69,92; p<0,001; I^2: 87,12%

Hepatomegaly-Right

Study name	Time point	Statistics for each study			
		Odds ratio	Lower limit	Upper limit	p-Value
Carlton 2010	24	0,299	0,201	0,444	0,000
Koukounari 2010	12	0,152	0,064	0,361	0,000
Stephnson1 1985	6	0,535	0,280	1,022	0,058
Stephnson1 1989	8	1,509	0,615	3,702	0,369
Stephnson2 1989	8	0,907	0,381	2,158	0,825
Sukwa 1987	16	0,581	0,444	0,759	0,000
Sukwa1 1993	12	0,108	0,024	0,473	0,003
Sukwa2 1993	12	0,120	0,015	0,968	0,047
Wiest 1994	12	0,646	0,511	0,817	0,000
Zhao 1995	12	1,670	1,242	2,245	0,001
		0,531	0,335	0,842	0,007

Odds ratio and 95% CI

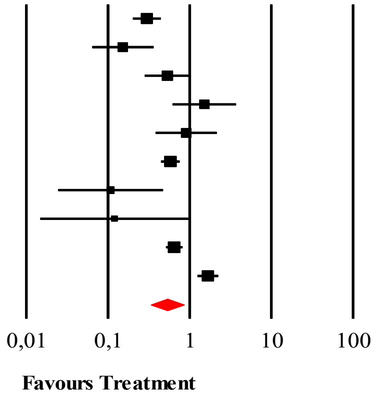

Effect estimation: Z: -2,688; p: 0,007
Heterogeneity: Q: 77,54; p<0,001; I^2: 88,39%

Hepatomegaly-no specific lobe

Study name	Time point	Odds ratio	Lower limit	Upper limit	p-Value
		Statistics for each study			
Butterworth1 1991	12	0,153	0,092	0,253	0,000
Butterworth2 1991	12	0,123	0,069	0,221	0,000
DeStigter 1989	84	0,133	0,060	0,294	0,000
Gryseels1 1994	12	1,399	1,112	1,761	0,004
Gryseels2 1994	12	1,265	1,004	1,595	0,046
Hadidjaja 1985	8	0,490	0,311	0,773	0,002
Homeida 1991	36	0,229	0,097	0,540	0,001
Ruiz-Guevara 2007	60	0,791	0,413	1,514	0,479
Sleigh 1986	12	0,223	0,123	0,406	0,000
		0,366	0,189	0,710	0,003

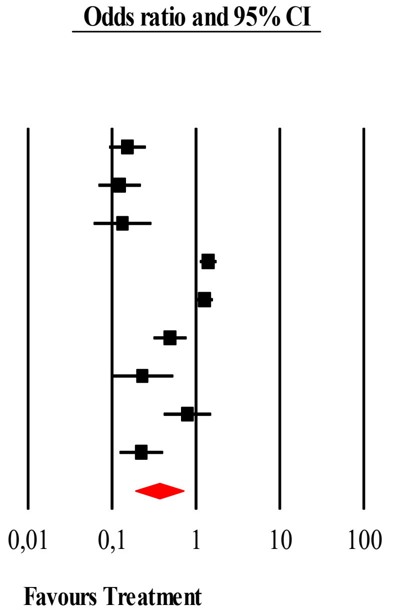

Effect estimation: Z: -4,104; p <0,001
Heterogeneity: Q: 167,27; p<0,001; I^2: 95,2%

Splenomegaly

Study name	Time point	Odds ratio	Lower limit	Upper limit	p-Value
		Statistics for each study			
Carlton 2010	24	0,809	0,397	1,647	0,559
Cook 1977	6	1,000	0,123	8,128	1,000
Cota 2006	48	0,608	0,227	1,630	0,323
DeStigter 1989	84	0,126	0,030	0,538	0,005
Doehring-Schwerdtf. 1992	23	1,187	0,863	1,632	0,292
Gryseels 1 1994	12	1,078	0,834	1,394	0,565
Gryseels 2 1994	12	1,138	0,917	1,413	0,240
Hadidjaja 1985	8	1,051	0,735	1,504	0,784
Homeida 1991	36	0,501	0,258	0,975	0,042
Koukounari A, 2010	12	0,264	0,184	0,379	0,000
Li YS 2000	24	0,365	0,095	1,398	0,141
Ruiz-Guevara 2007	60	0,153	0,008	3,015	0,217
Sleigh 1986	12	0,657	0,353	1,222	0,185
Stephenson 1 1989	8	2,015	1,160	3,502	0,013
Stephenson 1985	6	0,547	0,368	0,813	0,003
Stephenson 2 1989	8	1,322	0,759	2,301	0,324
Sukwa 1 1993	12	0,273	0,125	0,596	0,001
Sukwa 1987	16	0,570	0,422	0,769	0,000
Sukwa 2 1993	12	0,345	0,161	0,742	0,006
Wiest 1994	12	0,176	0,118	0,262	0,000
Zhao 1995	12	0,941	0,712	1,244	0,669
		0,631	0,470	0,846	0,002

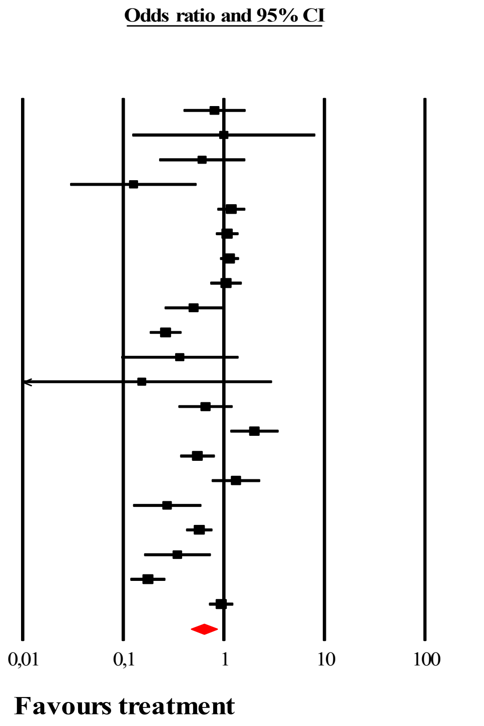

Effect estimation: Z: -3,079; p: 0,002
Heterogeneity: Q: 160,07; p<0,001; I^2: 87,50%

Periportal Fibrosis

Study name	Time point	Odds ratio	Lower limit	Upper limit	p-Value
		**Statistics for each study**			
Berhe 2008	26	0,005	0,000	0,077	0,000
Carlton 2010	24	1,186	0,629	2,234	0,598
Cota 2006	48	0,543	0,222	1,325	0,180
Doehring-Schwerd. 1992	7	0,922	0,668	1,273	0,622
Frenzel 1 1999	12	0,554	0,424	0,725	0,000
Frenzel 2 1999	31	0,375	0,246	0,573	0,000
Homeida 1991	12	0,134	0,007	2,667	0,188
Li YS 2000	24	0,684	0,457	1,024	0,065
Martins-Leite 2008	12	0,582	0,321	1,052	0,073
Rahoud 2010	39	0,007	0,000	0,120	0,001
Ruiz-Guevara 2007	60	0,129	0,042	0,395	0,000
Wiest 1994	12	0,458	0,359	0,585	0,000
		0,489	0,349	0,687	0,000

Odds ratio and 95% CI

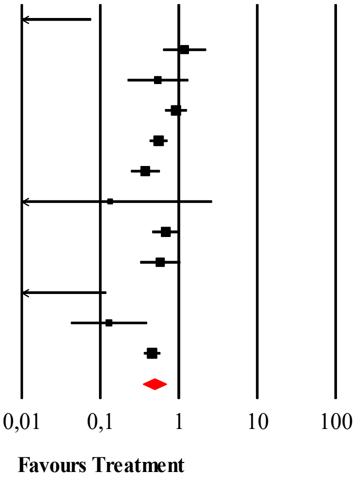

Effect estimation: Z = -4,136 (p = <0,001)
Heterogeneity: Q: 49,57; p<0,001; I^2: 77,81%

Main Portal Vein

Study name	Time point	Odds ratio	Lower limit	Upper limit	p-Value
		**Statistics for each study**			
Carlton 2010	24	1,379	0,911	2,088	0,129
Li YS 2000	24	0,672	0,374	1,209	0,185
Vennervald 2005	12	0,790	0,304	2,052	0,628
Kabatereine 2007	12	0,105	0,036	0,304	0,000
		0,575	0,232	1,425	0,232

Odds ratio and 95% CI

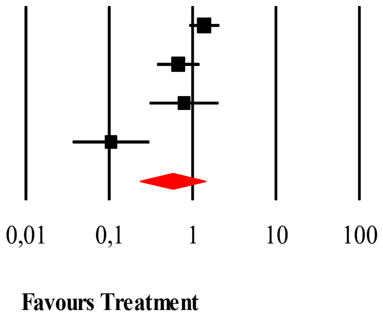

Effect estimation: Z: -1,195; p: 0.232
Heterogeneity: Q: 20,72; p<0,001; I^2: 85,52%

Diarrhea

Study name	Time point	Odds ratio	Lower limit	Upper limit	p-Value
Betson 2012	6	0,690	0,507	0,939	0,018
Cook 1977	6	0,111	0,019	0,659	0,016
Gryseels 1 1994	6	0,752	0,587	0,963	0,024
Gryseels 2 1994	6	0,657	0,497	0,869	0,003
Kongs 1996	12	0,337	0,237	0,478	0,000
Peixinho 1986	2	0,054	0,020	0,150	0,000
Sukwa 1987	16	0,372	0,274	0,505	0,000
Zhao 1995	12	1,884	1,406	2,523	0,000
		0,477	0,290	0,786	0,004

Statistics for each study

Odds ratio and 95% CI

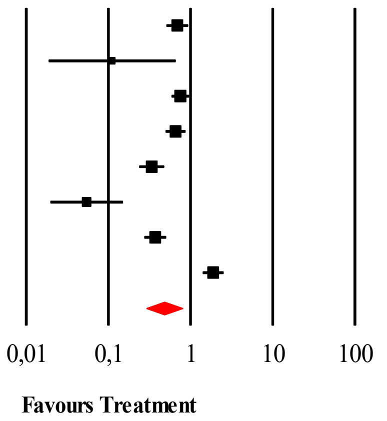

Effect estimation: Z = -2,908 (p: 0,004)
Heterogeneity: Q: 105,33; p<0,001; I^2: 93,35%

Blood in the Stool

Study name	Time point	Odds ratio	Lower limit	Upper limit	p-Value
Betson 2012	6	0,486	0,288	0,821	0,007
Boisier 1998	24	0,142	0,077	0,263	0,000
Sukwa 1987	16	0,137	0,070	0,270	0,000
Sukwa 1 1993	6	0,273	0,175	0,425	0,000
Sukwa 2 1993	6	0,356	0,228	0,556	0,000
Kongs 1996	12	0,159	0,102	0,247	0,000
Zhao 1995	12	0,684	0,521	0,897	0,006
Peixinho 1986	2	0,131	0,050	0,345	0,000
		0,257	0,156	0,421	0,000

Statistics for each study

Odds ratio and 95% CI

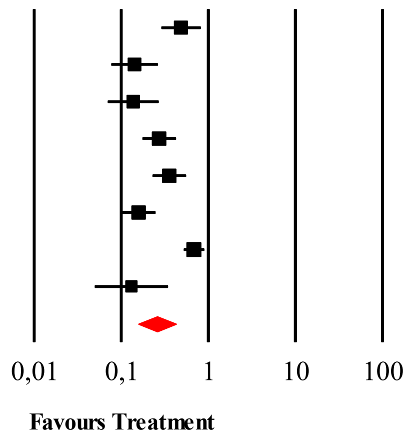

Effect estimation: Z: -5,378; p <0,001
Heterogeneity: Q: 57,90; p<0,001; I^2: 87,91%

Hematúria

Study name	Time point	Odds ratio	Lower limit	Upper limit	p-Value
		**Statistics for each study**			
Campagne 2001	5	0,046	0,023	0,094	0,000
Delegue 1998	4	0,204	0,116	0,359	0,000
Kahama 1999	6	0,027	0,012	0,061	0,000
Kiliku 1991	3	0,263	0,190	0,365	0,000
King 1 1990	12	0,072	0,056	0,092	0,000
King 2 1990	12	0,064	0,050	0,083	0,000
Kitange 1993	12	0,615	0,423	0,894	0,011
Koukounari 2007	12	0,120	0,096	0,150	0,000
Mekonnen 2013	2	0,002	0,000	0,037	0,000
Mott 1985	6	0,113	0,074	0,173	0,000
Resendramino 1998	12	0,175	0,131	0,234	0,000
Sarda 1987	6	0,005	0,001	0,026	0,000
Sissoko 2009	1	0,140	0,098	0,201	0,000
Stephenson 1984	6	0,037	0,021	0,063	0,000
Stete 2012	2	0,028	0,012	0,064	0,000
Tohon 2008	12	0,056	0,044	0,071	0,000
Wagatsuma 1999	6	0,049	0,038	0,063	0,000
		0,080	0,054	0,117	0,000

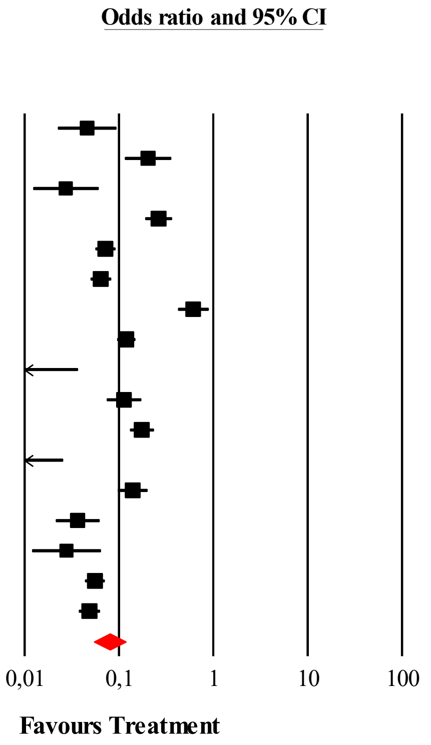

Effect estimation: Z: -13,233; p: <0,001

Heterogeneity: Q: 238,54; p<0,001; I^2: 93,29%

Proteinuria

Study name	Time point	Odds ratio	Lower limit	Upper limit	p-Value
		**Statistics for each study**			
Kiliku 1991	3	0,575	0,437	0,755	0,000
King 1 1990	12	0,158	0,127	0,197	0,000
King 2 1990	12	0,123	0,098	0,155	0,000
Kitange 1993	12	1,345	0,811	2,229	0,250
Mekonnen 2013	2	0,179	0,080	0,402	0,000
Mott 1985	6	0,075	0,044	0,125	0,000
Resendramino 1998	12	0,153	0,113	0,208	0,000
Sarda 1987	6	0,017	0,005	0,054	0,000
Stephenson 1984	6	0,052	0,032	0,082	0,000
Stete 2012	2	0,054	0,024	0,119	0,000
Wagatsuma 1999	6	0,005	0,002	0,009	0,000
		0,102	0,053	0,197	0,000

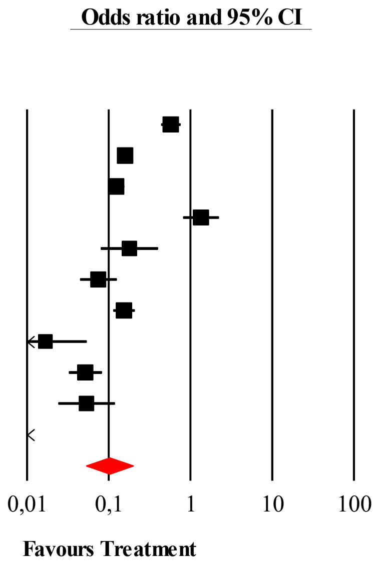

Effect estimation: Z: -6,780; p: <0,001

Heterogeneity: Q: 316,28; p<0,001; I^2: 96,83%

Urinary Bladder

Study name	Time point	Odds ratio	Lower limit	Upper limit	Z-Value	p-Value
		**Statistics for each study**				
Campagne 2001	2	0,110	0,054	0,221	-6,160	0,000
Delegue 1998	4	0,329	0,179	0,605	-3,577	0,000
Devidas 1989	10	0,120	0,071	0,203	-7,882	0,000
Doehring 1986	12	0,000	0,000	0,008	-5,302	0,000
Kahama 1999	4	0,158	0,087	0,287	-6,053	0,000
King 1988	12	0,315	0,194	0,511	-4,681	0,000
King 1 2002	9	0,240	0,092	0,623	-2,929	0,003
King 2 2002	9	0,178	0,058	0,544	-3,027	0,002
Ramarakoto 2008	6	0,126	0,072	0,221	-7,259	0,000
Resendramino 1998	12	0,192	0,142	0,260	-10,620	0,000
Reimert 2000	4	0,106	0,077	0,148	-13,311	0,000
Tohon 2008	12	0,241	0,201	0,289	-15,352	0,000
Traore 1998	12	0,515	0,390	0,681	-4,658	0,000
Hatz 1 1990	6	0,009	0,003	0,032	-7,548	0,000
Hatz 2 1990	6	0,005	0,001	0,038	-5,019	0,000
		0,140	0,095	0,208	-9,810	0,000

Odds ratio and 95% CI

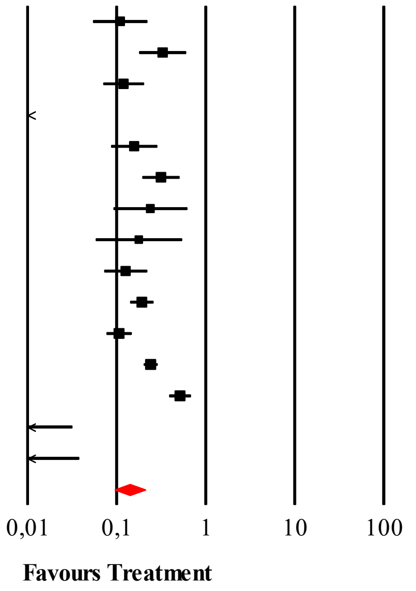

Effect estimation: Z: -9,810; p: <0,001

Heterogeneity: Q: 130,88; p<0,001; I^2: 89,30%

Upper urinary tract

Study name	Time point	Odds ratio	Lower limit	Upper limit	Z-Value	p-Value
		**Statistics for each study**				
Campagne 2001	34	0,129	0,044	0,382	-3,700	0,000
Delegue 1998	4	0,099	0,005	1,801	-1,563	0,118
Devidas 1989	10	0,220	0,129	0,374	-5,586	0,000
King 1988	12	1,163	0,774	1,749	0,727	0,467
King 1 2002	9	0,492	0,261	0,928	-2,191	0,028
King 2 2002	9	0,466	0,246	0,882	-2,347	0,019
Resendramino 1998	12	0,251	0,127	0,494	-3,996	0,000
Tohon 2008	12	0,069	0,025	0,190	-5,159	0,000
Traore 1998	12	0,254	0,169	0,383	-6,545	0,000
		0,286	0,160	0,510	-4,246	0,000

Odds ratio and 95% CI

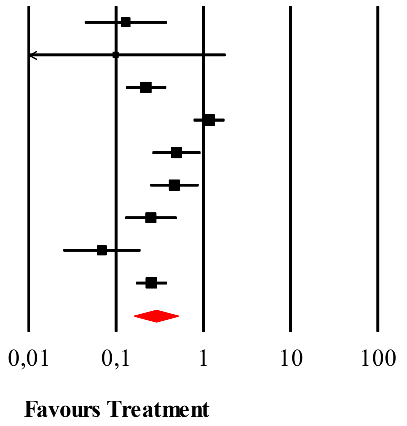

Effect estimation: Z: -4,246; p: <0,001

Heterogeneity: Q: 54,60; p<0,001; I^2: 85,35%
