# Supplementary material for: Decline in infection-related morbidities following drug-mediated reductions in the intensity of Schistosoma infection: A systematic review and meta-analysis
Source: PLoS Negl Trop Dis. 2017 Feb 17;11(2):e0005372. doi: 10.1371/journal.pntd.0005372 (PMC5333910; doi:10.1371/journal.pntd.0005372)
Supplement: S2 Fig — Forest plot showing sensitivity analysis, performed by removing one study at a time, for the effect of treatment on prevalence of left hepatic lobe hepatomegaly. (PDF) [file pntd.0005372.s007.pdf]

Study name	Time point	Point	Lower limit	Upper limit	p-Value
Carlton 2010	24	0,429	0,298	0,619	0,000
Doehring-Schwerdtf. 1992	7	0,454	0,308	0,671	0,000
Kabatereine 2007	12	0,420	0,299	0,590	0,000
Koukounari 2010	12	0,489	0,339	0,705	0,000
Li YS 2000	24	0,449	0,303	0,664	0,000
Sukwa 1987	16	0,455	0,299	0,694	0,000
Sukwa1 1993	12	0,516	0,360	0,740	0,000
Sukwa2 1993	12	0,514	0,357	0,738	0,000
Wiest 1994	12	0,501	0,346	0,726	0,000
Zhao 1995	12	0,461	0,297	0,715	0,001
		0,468	0,327	0,671	0,000

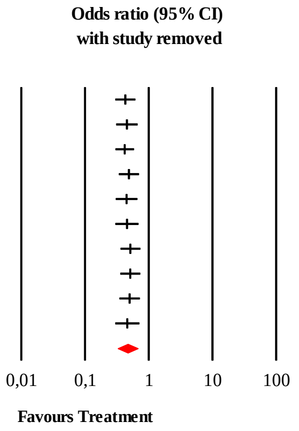

S2 Fig. Forest plot showing sensitivity analysis, performed by removing one study at a time, for the effect of treatment on prevalence of hepatomegaly (left hepatic lobe).
