# Supplementary material for: Decline in infection-related morbidities following drug-mediated reductions in the intensity of Schistosoma infection: A systematic review and meta-analysis
Source: PLoS Negl Trop Dis. 2017 Feb 17;11(2):e0005372. doi: 10.1371/journal.pntd.0005372 (PMC5333910; doi:10.1371/journal.pntd.0005372)
Supplement: S3 Fig — Forest plot showing sensitivity analysis, performed by removing one study at a time, for the effect of treatment on prevalence of right hepatic lobe hepatomegaly. (PDF) [file pntd.0005372.s008.pdf]

Study name	Time point	Statistics with study removed				Odds ratio (95% CI) with study removed
		Point	Lower limit	Upper limit	p-Value	
Carlton 2010	24	0,583	0,364	0,935	0,025	
Koukounari 2010	12	0,609	0,385	0,964	0,035	
Stephnson1 1985	6	0,527	0,319	0,872	0,013	
Stephnson1 1989	8	0,478	0,295	0,776	0,003	
Stephnson2 1989	8	0,500	0,305	0,820	0,006	
Sukwa 1987	16	0,508	0,287	0,897	0,020	
Sukwa1 1993	12	0,586	0,369	0,932	0,024	
Sukwa2 1993	12	0,562	0,352	0,896	0,015	
Wiest 1994	12	0,496	0,275	0,895	0,020	
Zhao 1995	12	0,466	0,319	0,680	0,000	
		0,531	0,335	0,842	0,007	

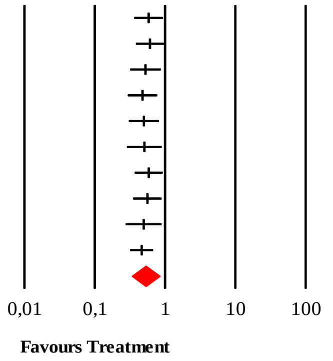

S3 Fig. Forest plot showing sensitivity analysis, performed by removing one study at a time, for the effect of treatment on prevalence of hepatomegaly (right hepatic lobe).
