# Supplementary material for: Decline in infection-related morbidities following drug-mediated reductions in the intensity of Schistosoma infection: A systematic review and meta-analysis
Source: PLoS Negl Trop Dis. 2017 Feb 17;11(2):e0005372. doi: 10.1371/journal.pntd.0005372 (PMC5333910; doi:10.1371/journal.pntd.0005372)
Supplement: S4 Fig — Forest plot showing sensitivity analysis, performed by removing one study at a time, for the effect of treatment on prevalence of hepatomegaly (lobe not specified). (PDF) [file pntd.0005372.s009.pdf]

Study name	Time point	Statistics with study removed			
		Point	Lower limit	Upper limit	p-Value
Butterworth1 1991	12	0,412	0,215	0,788	0,007
Butterworth2 1991	12	0,422	0,222	0,804	0,009
DeStigter 1989	84	0,412	0,210	0,810	0,010
Gryseels1 1994	12	0,304	0,142	0,653	0,002
Gryseels2 1994	12	0,308	0,138	0,685	0,004
Hadidjaja 1985	8	0,351	0,167	0,739	0,006
Homeida 1991	36	0,386	0,192	0,775	0,007
Ruiz-Guevara 2007	60	0,332	0,161	0,687	0,003
Sleigh 1986	12	0,389	0,194	0,780	0,008
		0,366	0,189	0,710	0,003

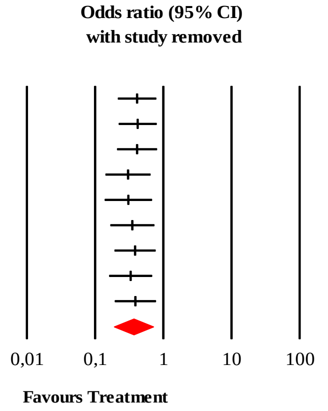

S4 Fig. Forest plot showing sensitivity analysis, performed by removing one study at a time, for the effect of treatment on prevalence of hepatomegaly (lobe not specified).
