# Supplementary material for: Decline in infection-related morbidities following drug-mediated reductions in the intensity of Schistosoma infection: A systematic review and meta-analysis
Source: PLoS Negl Trop Dis. 2017 Feb 17;11(2):e0005372. doi: 10.1371/journal.pntd.0005372 (PMC5333910; doi:10.1371/journal.pntd.0005372)
Supplement: S5 Fig — Forest plot showing sensitivity analysis, performed by removing one study at a time, for the effect of treatment on prevalence of splenomegaly. (PDF) [file pntd.0005372.s010.pdf]

Study name	Time point	Statistics with study removed: Point	Lower limit	Upper limit	p-Value
Carlton 2010	24	0,622	0,459	0,843	0,002
Cook 1977	6	0,626	0,465	0,842	0,002
Cota 2006	48	0,631	0,467	0,853	0,003
DeStigter 1989	84	0,658	0,491	0,882	0,005
Doehring-Schwerdtf. 1992	23	0,604	0,444	0,823	0,001
Gryseels 1 1994	12	0,606	0,443	0,831	0,002
Gryseels 2 1994	12	0,604	0,442	0,827	0,002
Hadidjaja 1985	8	0,609	0,447	0,831	0,002
Homeida 1991	36	0,638	0,471	0,864	0,004
Koukounari A, 2010	12	0,674	0,510	0,891	0,006
Li YS 2000	24	0,641	0,476	0,863	0,003
Ruiz-Guevara 2007	60	0,638	0,476	0,857	0,003
Sleigh 1986	12	0,629	0,463	0,853	0,003
Stephenson 1 1989	8	0,593	0,441	0,796	0,001
Stephenson 1985	6	0,635	0,466	0,864	0,004
Stephenson 2 1989	8	0,605	0,447	0,819	0,001
Sukwa 1 1993	12	0,657	0,488	0,884	0,006
Sukwa 1987	16	0,632	0,462	0,865	0,004
Sukwa 2 1993	12	0,649	0,481	0,876	0,005
Wiest 1994	12	0,698	0,540	0,903	0,006
Zhao 1995	12	0,611	0,445	0,840	0,002
		0,631	0,470	0,846	0,002

Odds ratio (95% CI) with study removed

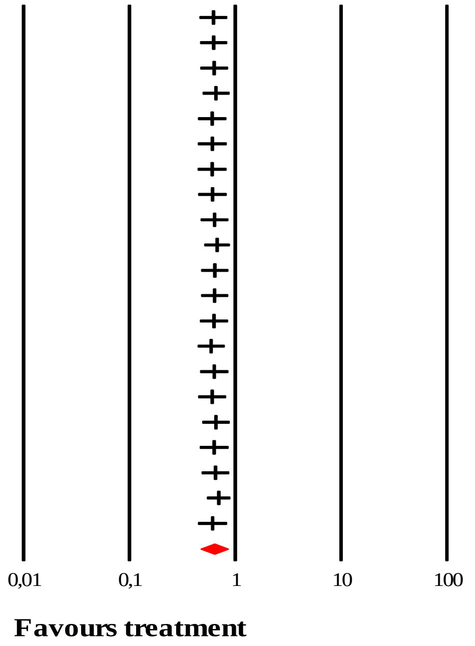

S5 Fig. Forest plot showing sensitivity analysis, performed by removing one study at a time, for the effect of treatment on prevalence of splenomegaly.
