# Supplementary material for: Decline in infection-related morbidities following drug-mediated reductions in the intensity of Schistosoma infection: A systematic review and meta-analysis
Source: PLoS Negl Trop Dis. 2017 Feb 17;11(2):e0005372. doi: 10.1371/journal.pntd.0005372 (PMC5333910; doi:10.1371/journal.pntd.0005372)
Supplement: S6 Fig — Forest plot showing sensitivity analysis, performed by removing one study at a time, for the effect of treatment on prevalence of periportal fibrosis (PDF) [file pntd.0005372.s011.pdf]

Study name	Time point	Point	Lower limit	Upper limit	p-Value
Berhe 2008	26	0,497	0,383	0,646	0,000
Carlton 2010	24	0,429	0,320	0,577	0,000
Cota 2006	48	0,455	0,330	0,628	0,000
Doehring-Schwerd. 1992	23	0,450	0,316	0,640	0,000
Frenzel 1 1999	12	0,435	0,301	0,627	0,000
Frenzel 2 1999	31	0,472	0,338	0,660	0,000
Homeida 1991	12	0,469	0,345	0,636	0,000
Li YS 2000	24	0,435	0,311	0,607	0,000
Martins-Leite 2008	12	0,448	0,322	0,623	0,000
Rahoud 2010	39	0,493	0,376	0,647	0,000
Ruiz-Guevara 2007	60	0,501	0,374	0,670	0,000
Wiest 1994	12	0,445	0,307	0,646	0,000
		0,464	0,343	0,627	0,000

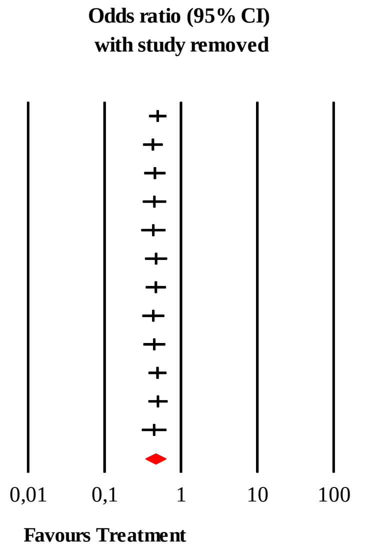

S6 Fig. Forest plot showing sensitivity analysis, performed by removing one study at a time, for the effect of treatment on prevalence of periportal fibrosis.
