# Supplementary material for: Decline in infection-related morbidities following drug-mediated reductions in the intensity of Schistosoma infection: A systematic review and meta-analysis
Source: PLoS Negl Trop Dis. 2017 Feb 17;11(2):e0005372. doi: 10.1371/journal.pntd.0005372 (PMC5333910; doi:10.1371/journal.pntd.0005372)
Supplement: S7 Fig — Forest plot showing sensitivity analysis, performed by removing one study at a time, for the effect of treatment on prevalence of portal vein dilation. (PDF) [file pntd.0005372.s012.pdf]

Study name	Time point	Statistics with study removed			
		Point	Lower limit	Upper limit	p-Value
Carlton 2010	24	0,403	0,132	1,227	0,110
Li YS 2000	24	0,516	0,122	2,181	0,368
Vennervald 2005	12	0,509	0,157	1,653	0,261
Kabatereine 2007	12	0,961	0,575	1,605	0,878
		0,575	0,232	1,425	0,232

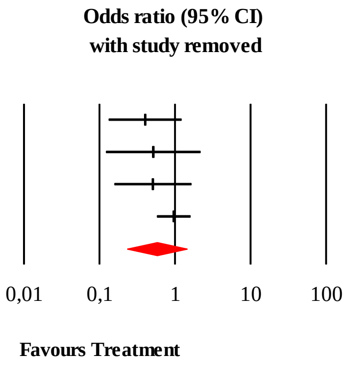

S7 Fig. Forest plot showing sensitivity analysis, performed by removing one study at a time, for the effect of treatment on prevalence of portal vein dilation.
