# Supplementary material for: Decline in infection-related morbidities following drug-mediated reductions in the intensity of Schistosoma infection: A systematic review and meta-analysis
Source: PLoS Negl Trop Dis. 2017 Feb 17;11(2):e0005372. doi: 10.1371/journal.pntd.0005372 (PMC5333910; doi:10.1371/journal.pntd.0005372)
Supplement: S8 Fig — Forest plot showing sensitivity analysis, performed by removing one study at a time, for the effect of treatment on prevalence of diarrhea after treatment. (PDF) [file pntd.0005372.s013.pdf]

Study name	Time point	Statistics with study removed			
		Point	Lower limit	Upper limit	p-Value
Betson 2012	6	0,437	0,242	0,789	0,006
Cook 1977	6	0,518	0,312	0,859	0,011
Gryseels 1 1994	6	0,427	0,231	0,787	0,006
Gryseels 2 1994	6	0,438	0,240	0,800	0,007
Kongs 1996	12	0,506	0,296	0,864	0,013
Peixinho 1986	2	0,604	0,378	0,966	0,035
Sukwa 1987	16	0,495	0,287	0,856	0,012
Zhao 1995	12	0,412	0,275	0,617	0,000
		0,477	0,290	0,786	0,004

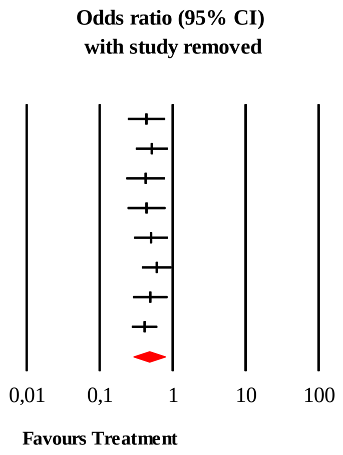

S8 Fig. Forest plot showing sensitivity analysis, performed by removing one study at a time, for the effect of treatment on prevalence of diarrhea.
