# Supplementary material for: Decline in infection-related morbidities following drug-mediated reductions in the intensity of Schistosoma infection: A systematic review and meta-analysis
Source: PLoS Negl Trop Dis. 2017 Feb 17;11(2):e0005372. doi: 10.1371/journal.pntd.0005372 (PMC5333910; doi:10.1371/journal.pntd.0005372)
Supplement: S9 Fig — Forest plot showing sensitivity analysis, performed by removing one study at a time, for the effect of treatment on prevalence of blood in stool. (PDF) [file pntd.0005372.s014.pdf]

Study name	Time point	Statistics with study removed			
		Point	Lower limit	Upper limit	p-Value
Betson 2012	12	0,211	0,170	0,262	0,000
Boisier 1998	24	0,298	0,242	0,367	0,000
Sukwa 1987	16	0,295	0,240	0,362	0,000
Sukwa 1 1993	6	0,277	0,223	0,345	0,000
Sukwa 2 1993	6	0,260	0,208	0,323	0,000
Kongs 1996	12	0,317	0,254	0,395	0,000
Peixinho 1986	2	0,285	0,233	0,349	0,000
		0,276	0,227	0,336	0,000

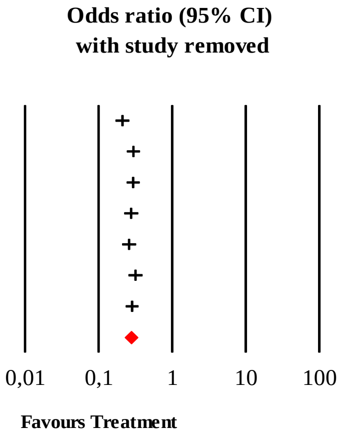

S9 Fig. Forest plot showing sensitivity analysis, performed by removing one study at a time, for the effect of treatment on prevalence of blood in stool.
