# Supplementary material for: Decline in infection-related morbidities following drug-mediated reductions in the intensity of Schistosoma infection: A systematic review and meta-analysis
Source: PLoS Negl Trop Dis. 2017 Feb 17;11(2):e0005372. doi: 10.1371/journal.pntd.0005372 (PMC5333910; doi:10.1371/journal.pntd.0005372)
Supplement: S10 Fig — Forest plot showing sensitivity analysis, performed by removing one study at a time, for the effect of treatment on prevalence of blood in urine. (PDF) [file pntd.0005372.s015.pdf]

Study name	Time point	Statistics with study removed			
		Point	Lower limit	Upper limit	p-Value
Campagne 2001	5	0,082	0,055	0,122	0,000
Delegue 1998	4	0,075	0,051	0,112	0,000
Kahama 1999	6	0,085	0,057	0,125	0,000
Kiliku 1991	3	0,074	0,050	0,108	0,000
King 1 1990	12	0,079	0,052	0,121	0,000
King 2 1990	12	0,080	0,053	0,121	0,000
Kitange 1993	12	0,072	0,052	0,100	0,000
Koukounari 2007	12	0,076	0,050	0,116	0,000
Mekonnen 2013	2	0,084	0,058	0,124	0,000
Mott 1985	6	0,077	0,052	0,116	0,000
Resendramino 1998	12	0,075	0,050	0,112	0,000
Sarda 1987	6	0,088	0,060	0,128	0,000
Sissoko 2009	1	0,076	0,051	0,114	0,000
Stephenson 1984	6	0,084	0,057	0,125	0,000
Stete 2012	2	0,085	0,057	0,125	0,000
Tohon 2008	12	0,081	0,054	0,122	0,000
Wagatsuma 1999	6	0,083	0,055	0,123	0,000
		0,080	0,054	0,117	0,000

Odds ratio (95% CI) with study removed

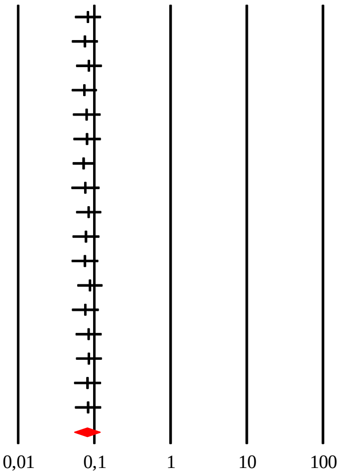

Favours Treatment

S10 Fig. Forest plot showing sensitivity analysis, performed by removing one study at a time, for the effect of treatment on prevalence of blood in urine.
