# Supplementary material for: Decline in infection-related morbidities following drug-mediated reductions in the intensity of Schistosoma infection: A systematic review and meta-analysis
Source: PLoS Negl Trop Dis. 2017 Feb 17;11(2):e0005372. doi: 10.1371/journal.pntd.0005372 (PMC5333910; doi:10.1371/journal.pntd.0005372)
Supplement: S11 Fig — Forest plot showing sensitivity analysis, performed by removing one study at a time, for the effect of treatment on prevalence of protein in urine. (PDF) [file pntd.0005372.s016.pdf]

Study name	Time point	Point	Lower limit	Upper limit	p-Value
Kiliku 1991	3	0,103	0,058	0,182	0,000
King 1 1990	12	0,115	0,055	0,242	0,000
King 2 1990	12	0,119	0,057	0,247	0,000
Kitange 1993	12	0,095	0,053	0,170	0,000
Mekonnen 2013	2	0,116	0,060	0,222	0,000
Mott 1985	6	0,126	0,065	0,243	0,000
Resendramino 1998	12	0,116	0,058	0,234	0,000
Sarda 1987	6	0,140	0,075	0,264	0,000
Stephenson 1984	6	0,131	0,068	0,251	0,000
Stete 2012	2	0,129	0,068	0,247	0,000
Wagatsuma 1999	18	0,140	0,079	0,249	0,000
		0,120	0,065	0,223	0,000

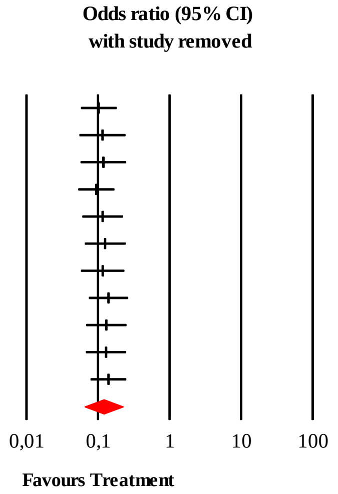

S11 Fig. Forest plot showing sensitivity analysis, performed by removing one study at a time, for the effect of treatment on prevalence of protein in urine.
