# Supplementary material for: Decline in infection-related morbidities following drug-mediated reductions in the intensity of Schistosoma infection: A systematic review and meta-analysis
Source: PLoS Negl Trop Dis. 2017 Feb 17;11(2):e0005372. doi: 10.1371/journal.pntd.0005372 (PMC5333910; doi:10.1371/journal.pntd.0005372)
Supplement: S12 Fig — Forest plot showing sensitivity analysis, performed by removing one study at a time, for the effect of treatment on prevalence of ultrasound abnormalities of the urinary bladder. (PDF) [file pntd.0005372.s017.pdf]

Study name	Time point	Statistics with study removed				
		Point	Lower limit	Upper limit	Z-Value	p-Value
Campagne 2001	2	0,158	0,107	0,232	-9,360	0,000
Delegue 1998	4	0,144	0,097	0,213	-9,666	0,000
Devidas 1989	10	0,157	0,106	0,232	-9,308	0,000
Doehring 1986	12	0,171	0,120	0,242	-9,912	0,000
Kahama 1999	12	0,144	0,097	0,215	-9,568	0,000
King 1988	12	0,143	0,096	0,213	-9,576	0,000
King 1 2002	9	0,149	0,101	0,220	-9,618	0,000
King 2 2002	9	0,152	0,103	0,224	-9,574	0,000
Ramarakoto 2008	6	0,156	0,105	0,230	-9,318	0,000
Resendramino 1998	12	0,146	0,096	0,222	-9,018	0,000
Reimert 2000	12	0,153	0,102	0,229	-9,127	0,000
Tohon 2008	12	0,138	0,087	0,217	-8,528	0,000
Traore 1998	12	0,142	0,100	0,203	-10,793	0,000
Hatz 1 1990	6	0,182	0,129	0,257	-9,727	0,000
Hatz 2 1990	6	0,170	0,118	0,243	-9,674	0,000
		0,154	0,106	0,223	-9,850	0,000

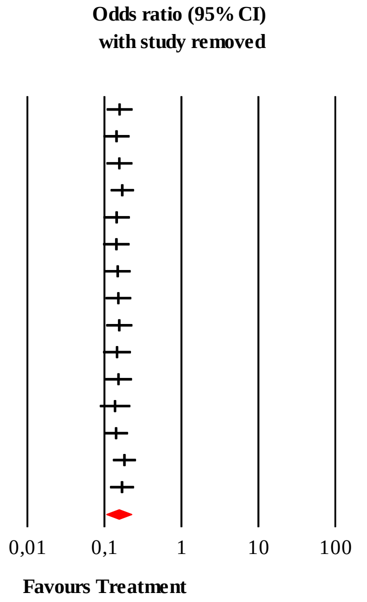

S12 Fig. Forest plot showing sensitivity analysis, performed by removing one study at a time, for the effect of treatment on prevalence of ultrasound abnormalities of the urinary bladder.
