# Supplementary material for: Decline in infection-related morbidities following drug-mediated reductions in the intensity of Schistosoma infection: A systematic review and meta-analysis
Source: PLoS Negl Trop Dis. 2017 Feb 17;11(2):e0005372. doi: 10.1371/journal.pntd.0005372 (PMC5333910; doi:10.1371/journal.pntd.0005372)
Supplement: S13 Fig — Forest plot showing sensitivity analysis, performed by removing one study at a time, for the effect of treatment on prevalence of ultrasound abnormalities in the upper urinary tract. (PDF) [file pntd.0005372.s018.pdf]

Study name	Time point	Statistics with study removed				
		Point	Lower limit	Upper limit	Z-Value	p-Value
Campagne 2001	34	0,312	0,170	0,570	-3,784	0,000
Delegue 1998	4	0,296	0,164	0,533	-4,049	0,000
Devidas 1989	10	0,294	0,154	0,564	-3,690	0,000
King 1988	12	0,246	0,167	0,364	-7,055	0,000
King 1 2002	9	0,262	0,136	0,504	-4,005	0,000
King 2 2002	9	0,264	0,137	0,509	-3,975	0,000
Resendramino 1998	12	0,288	0,151	0,550	-3,773	0,000
Tohon 2008	12	0,339	0,193	0,595	-3,768	0,000
Traore 1998	12	0,286	0,145	0,565	-3,605	0,000
		0,286	0,160	0,510	-4,246	0,000

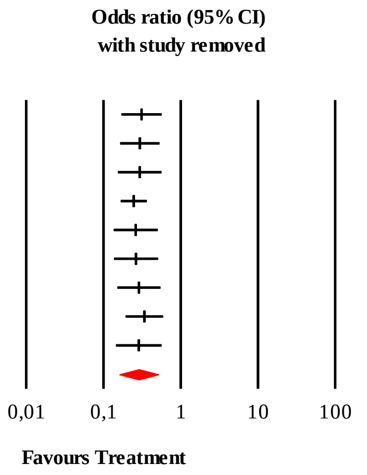

S13 Fig. Forest plot showing sensitivity analysis, performed by removing one study at a time, for the effect of treatment on prevalence of ultrasound abnormalities in the upper urinary tract.
