# Supplementary material for: Decline in infection-related morbidities following drug-mediated reductions in the intensity of Schistosoma infection: A systematic review and meta-analysis
Source: PLoS Negl Trop Dis. 2017 Feb 17;11(2):e0005372. doi: 10.1371/journal.pntd.0005372 (PMC5333910; doi:10.1371/journal.pntd.0005372)
Supplement: S14 Fig — Forest plot showing sensitivity analysis, performed by removing one study at a time, for the effect of treatment on blood hemoglobin. (PDF) [file pntd.0005372.s019.pdf]

Study name	Time point	Statistics with study removed			
		Point	Lower limit	Upper limit	p-Value
Ayoya 2009	3	0,618	-0,257	1,494	0,166
Beasley 1999	4	0,769	-0,079	1,617	0,075
Bhargava 1 2003	3	0,662	-0,207	1,532	0,135
Bhargava 2 2003	3	0,640	-0,243	1,523	0,155
Koukounari 2007	12	0,323	-0,052	0,699	0,092
McGarvey 1996	6	0,657	-0,208	1,522	0,137
Sissoko 2009	1	0,648	-0,290	1,585	0,176
Stephenson 1 1989	8	0,662	-0,212	1,537	0,138
Stephenson 1985	6	0,455	-0,397	1,308	0,295
Stephenson 2 1989	8	0,590	-0,286	1,465	0,187
Mwanakasale 2009	9	0,613	-0,279	1,505	0,178
Kabatereine 2007	12	0,650	-0,358	1,657	0,206
		0,607	-0,211	1,426	0,146

Std diff in means (95% CI) with study removed

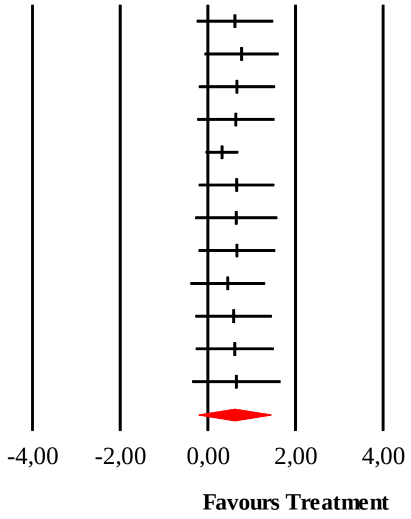

S14 Fig. Forest plot showing sensitivity analysis, performed by removing one study at a time, for the effect of treatment on blood hemoglobin.
